# Shedding Light on the Phytochemical and Biological Fingerprints of *Fibigia clypeata* (L.) Medik Essential Oil as a Pharmacotherapeutic Agent

**DOI:** 10.1002/fsn3.70493

**Published:** 2025-07-04

**Authors:** Tuba Unver, Murat Bingul, Harun Uslu, Ismet Gurhan, Bunyamin Goktas, Hasan Sahin, Mehmet Boga

**Affiliations:** ^1^ Department of Pharmaceutical Microbiology, Faculty of Pharmacy Inonu University Malatya Turkiye; ^2^ Department of Basic Pharmaceutical Sciences, Faculty of Pharmacy Dicle University Diyarbakır Turkiye; ^3^ Department of Pharmaceutical Chemistry, Faculty of Pharmacy Fırat University Elazığ Turkiye; ^4^ Department of Pharmaceutical Botany, Faculty of Pharmacy Inonu University Malatya Turkiye; ^5^ Department of Pharmacognosy, Faculty of Pharmacy Dicle University Diyarbakır Turkiye; ^6^ Department of Analytical Chemistry, Faculty of Pharmacy Dicle University Diyarbakır Turkiye

**Keywords:** ADME predictions, anticholinesterase activity, antimicrobial agents, antioxidants, dimethyl disulfide, dimethyl trisulfide, *Fibigia clypeata*, molecular docking

## Abstract

Since plant essential oil contains medicinally valuable compounds, its usability as a pharmacotherapeutic agent has been the focus of attention within the century's needs. The lack of sufficient studies on the medical and pharmacological evaluation of *Fibigia clypeata* (L.) Medik has made this plant the target of our study. This study analyzes the phytochemical composition and biological activity of *F. clypeata* essential oil. As a result, dimethyl disulfide and dimethyl trisulfide were found to be the main compounds of the plant essential oil, with rates of 73.13% and 19.87%, respectively. The antifungal property of plant essential oil is more effective than its antibacterial property, with MIC values ranging between 0.039 and 0.312 μL/mL for fungal species and up to 3.750 μL/mL for bacterial species. The enzyme inhibition profiles were investigated towards two enzymes, namely, anticholinesterase and α‐glucosidase, targeted for anti‐diabetic studies. Anticholinesterase activity was proved with the IC_50_ values of 17.31 and 4.78 μg/mL for Acetylcholinesterase (AChE) and Butyrylcholinesterase (BChE) enzymes, respectively. DPPH and CUPRAC activities were the most promising antioxidant studies, with values of 1.54 and 3.72 μg/mL. It was observed that α‐Terpineol made a hydrogen bond with ASN80, and 1‐(2,6,6‐Trimethyl‐1,3‐cyclohexadien‐1‐yl)ethanol made a hydrogen bond with SER82. Although molecular dock scores were better for antifungal activity, it was determined that no interactions, such as hydrogen bonding or pi interaction, were observed. This preliminary study showed that *F. clypeata* essential oil is a natural source with promising in vitro antimicrobial, antioxidant, and anticholinesterase activities that warrants further investigation, including safety assessments, due to the high concentration of sulfur‐containing compounds. Molecular docking and ADME prediction results showed that α‐Terpineol and 1‐(2,6,6‐Trimethyl‐1,3‐cyclohexadien‐1‐yl)ethanol were more prone to antimicrobial activity.

## Graphical abstract

This study aims to analyze the phytochemical composition and in silico and in vitro biological activity of *Fibigia clypeata* (L.) Medik essential oil is a natural pharmaceutical source for the first time in the literature. GC–MS Analysis showed that dimethyl disulfide and dimethyl trisulfide were the main compounds of the plant essential oil. The antifungal properties of plant essential oil are more effective than its antibacterial properties. Anticholinesterase activity was proved and free radical scavenging activity and Cupric ion‐reducing capacity were the most promising antioxidant studies.

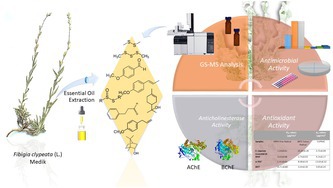

## 1 Introduction

All over the world, bioactive compounds obtained from natural products, including plants, are widespread in the production of medicines. The use of secondary metabolites obtained from plants in the medical, pharmaceutical, and cosmetic fields, as well as food preservatives, is widely accepted. Many studies have reported that phenolic substances obtained from plants play an essential role in plant biological activity (Mekinić et al. 2019; Hussain et al. 2021; Abdalla and Zidorn 2020). Eighty per cent of the world's population utilizes traditional medicines, especially medicinal plants, in primary health care (Alqahtani et al. 2019). The biological activities of extracts and essential oils obtained from aromatic and medicinal plants have been proven, and these plants have attracted particular attention due to their radical scavenging effects (de Sousa et al. 2015). Various pathologies such as cancer, neurodegenerative diseases, and deterioration of food quality are attributed to free radicals (Hale et al. 2008). The intensive use of antibiotics has led to serious side effects in public health and the emergence of antibiotic resistance, which is another problem impacting public health (Chouhan et al. 2017; De Billerbeck 2007). For all these reasons, scientists have turned to investigating the existence of natural agents with reduced side effects, no economic burden, and high effectiveness. This has led to a fascinating exploration of plants as natural sources that can supply a wide range of complicated and structurally diverse compounds easily found in nature. With their proven antimicrobial properties, plant extracts and essential oils have emerged as potential sources of new antimicrobial compounds. This global exploration of plants as alternative products in food preservation and treatment of infectious diseases is a testament to the exciting potential of these natural agents (Safaei‐Ghomi and Ahd 2010; Astani et al. 2010). Essential oils obtained from various plants have been proven to have powerful antioxidant, antiseptic, antimicrobial, antiviral, insecticidal and antiparasitic properties (Unver and Gurhan 2024a; Chouhan et al. 2017; Kaloustian et al. 2008; Burt 2004; Benjilali and Ayadi 1986). For this reason, essential oils have the potential to be used as an effective agent to reduce bacterial resistance in public health and the food industry (Stefanakis et al. 2013). Essential oils are obtained from the underground or aboveground parts of the plant. They are substances whose density is generally lower than water and are soluble in oil and organic solvents.

The *Fibigia* genus, one of the Brassicaceae family genera, is represented by approximately 16 taxa worldwide (Rechinger 1968; Cullen 1965; Tutin and Heywood 1964; Bouloumoy 1930; Bush 1939). This genus is localized in the Eastern Mediterranean and Iran–Turan phytogeographic regions (Bush 1939). In Turkiye, this genus has four naturally occurring species (Cullen 1965). The geographical distribution of *Fibigia clypeata* (L.) Medik, a perennial herbaceous, woody plant at the base, is determined in Turkiye, Russia, Europe, Iraq, Iran, Egypt, Palestine, Lebanon, and Syria (Tuştaş 2008). Although morphological and phylogenetic studies of the *F. clypeata* species have been carried out so far, there are few studies on the medical and pharmacological evaluation of the *F. clypeata* . Almost the only study that contributed to the literature in this sense was Zengin et al. (2020). In that study, the antioxidant and inhibitory activities of aqueous, ethyl acetate, and methanol extracts of *F. clypeata* were determined against necessary enzymes (α‐amylase, tyrosinase, α‐glucosidase, acetylcholinesterase, and butyrylcholinesterase) targeted in the treatment of type II diabetes, skin hyperpigmentation, and Alzheimer's disease. As a result, it has been determined that extracts obtained from *F. clypeata* may be a novel pharmacotherapeutic agent for Alzheimer's disease, skin hyperpigmentation problems, type II diabetes, and cancer diseases (Zengin et al. 2020).

This study is the first and guiding study showing that the essential oil of *F. clypeata* can be used as a valuable natural therapeutic agent in the pharmacological and medical fields. In this study, firstly, the essential oil constituents of *F. clypeata* were revealed by Gas Chromatography–Mass Spectrometry (GC–MS) analysis, and then its antibacterial and antifungal properties were examined by the broth microdilution method. Based on the reported biological properties of different extracts of *F. clypeata* towards the anticholinesterase and α‐glucosidase enzymes, it was of interest to investigate the enzyme inhibition potentials of the essential oil of *F. clypeata* in order to generate a complementary explanation for the pharmaceutical potency. In addition, free and cationic radical scavenging potencies, as well as the reductive behaviors towards the Cu metal of the targeted oil, were also examined by the abovementioned assays. The results were detailed with molecular docking and ADME prediction studies.

## 2 Materials and Methods

### 2.1 Plant Material and Isolation of Essential Oil

**FIGURE 1:**
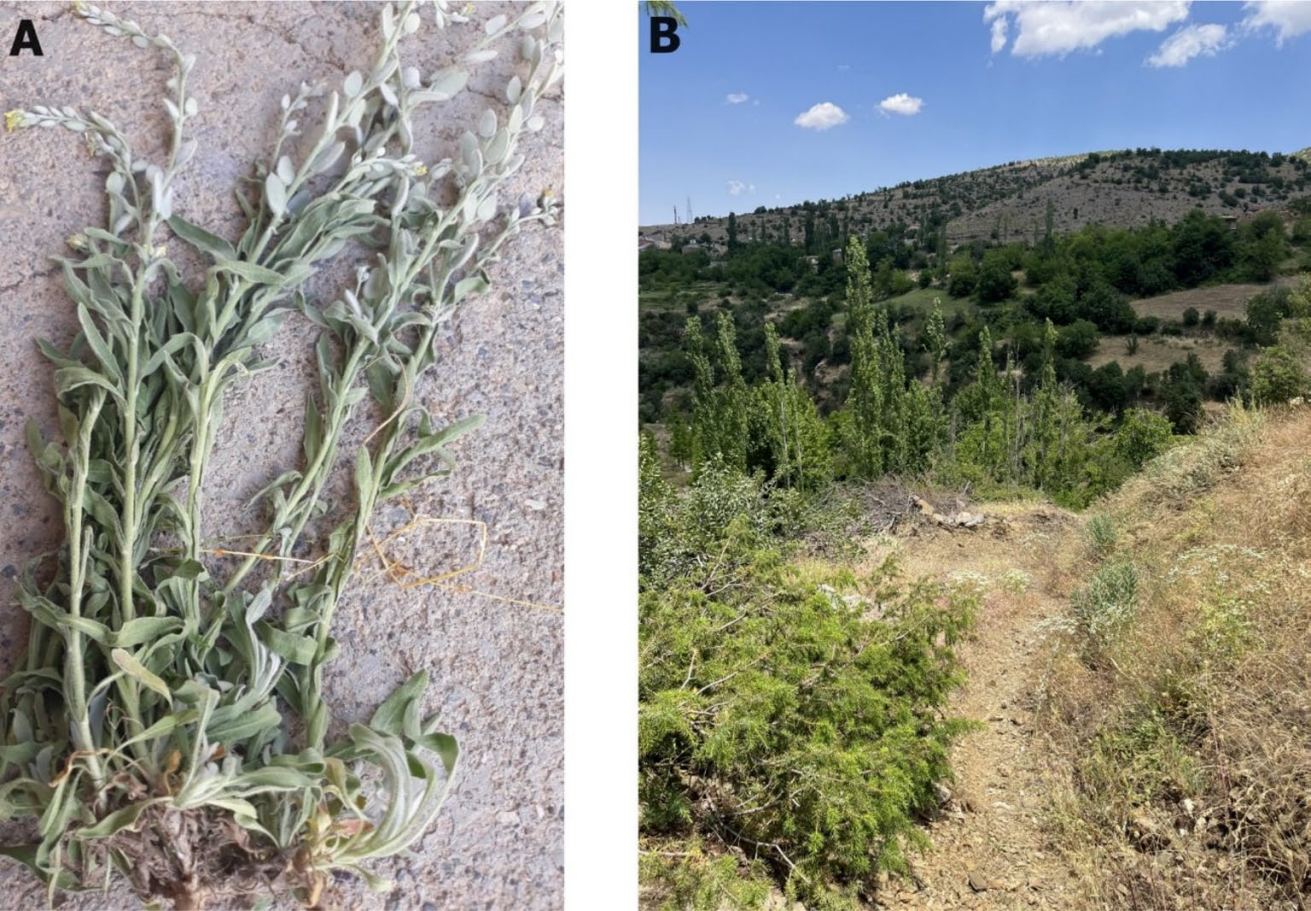
(A) The plant *Fibigia clypeata* (L.) Medik (B) The nature where *F. clypeata* grows and is collected (Merap mountain slopes and Kurucay, Puturge, Malatya, Turkiye).

The plant *F. clypeata* (L.) Medik was collected from the Merap mountain slopes and Puturge‐Kurucay in Malatya (Turkiye) in May 2023 (Figure 1). The plant was identified at Inonu University, Faculty of Pharmacy, Pharmaceutical Botany Laboratory (Voucher specimen codes: TU1002) (Matthews et al. 1975). As a first step, above‐ground parts of the plant (stem, leaf, seed, and flower) were dried and crushed with a grinder. Essential oil was acquired by water distillation using a Clevenger apparatus, using the dried above‐ground parts of the plant. For this purpose, 100 g of the dry herb was roughly chopped and placed in a glass flask. Then, approximately 10 times more distilled water was added and distilled for about 3.5 h. The amount of essential oil formed at the end of the period was determined.

### 2.2 Phytochemical Composition of Essential Oil (GC–MS)

0.5 g of *F. clypeata* aboveground parts were taken and sealed in an airtight vial. For the determination of volatile components of the aerial parts of the *F. clypeata* plant, Agilent Technologies 7000 GC/MS Triple Quad with 7890 GC, 7693 Autosampler, and 7697A Headspace Sampler, HP‐5 ms (30 m × 0.25 mm × 0.25 μm) was used. The temperature program for the separation process in the column was to be 60°C (4 min waiting), heating up to 220°C at a rate of 4°C/min, and holding at this temperature for 10 min. The total analysis time was 44 min. Library searching was performed using NIST MS SEARCH 2.0 software.

### 2.3 Determination of Antimicrobial Activity

#### 2.3.1 Strains and Media

Microorganisms, including four bacteria [ *Enterobacter aerogenes* (ATCC 51697), *Staphylococcus aureus* (ATCC 12600), *Escherichia coli* (C2987) and *Klebsiella pneumoniae* (ATCC 13883) and four yeast [ *Candida albicans* (ATCC 14053), *Candida parapsilosis* (ATCC 22019), *Candida krusei* (ATCC 14243), and *Candida tropicalis* (ATCC 13803)] were used to determine the antimicrobial activity of *F. clypeata* essential oil. *E. coli* was obtained from New England Biolabs (NEB, MA, US). Other microorganisms were acquired from the American Type Culture Collection (ATCC, VA, US). The tested bacterial microorganisms were subcultured on Muller Hinton agar medium, and the fungal cells were subcultured on Sabouraud 4% glucose agar by incubating overnight at 36°C. Subsequently, Muller Hinton broth and Sabouraud broth were used for antibacterial and antifungal assays, respectively.

#### 2.3.2 Antibacterial and Antifungal Activity

The minimum inhibitory concentration (MIC) of *F. clypeata* essential oil was determined using the broth and agar dilutions methods as applied before with minor modification (Unver, Uzuner, et al. 2024; Unver, Uslu, et al. 2024; Unver and Gurhan 2024b; CLSI, 2018).

##### 2.3.2.1 Broth Dilution Assays

In the broth dilution method, 12 μL of essential oil was dissolved in 200 μL of dimethylsulfoxide (DMSO) (Honeywell, Germany). Then, 50 μL of this solution was added to the first wells in the first row of the microplate. 150 μL of Muller Hinton broth was added to each of the first wells, and twofold dilutions were made from the 1st to the 10th well. *F. clypeata* essential oil concentration in the wells ranged from 15 to 0.029 μL/mL. Samples were prepared from each bacterial species, including *S. aureus* , *E. aerogenes* , *K. pneumoniae* , and *E. coli* , at a density of 0.5 McFarland (1–1.5 × 10^8^ cfu/mL). Then, 1 μL of these samples was inoculated into wells 1 to 10 of A, B, C, and D rows on the microplate. The 11th well is a positive control that confirms the viability of the microorganism and does not contain essential oil. The 12th well is the negative control, showing no contamination during the analysis. The microplate was left for incubation overnight at 36°C. After overnight, 10 μL of resazurin sodium salt (0.15% w/v) (Sigma‐Aldrich, USA, CAS No: 62758‐13‐8) was added to each well and waited 3–4 h to observe the color change that indicates the growth of the microorganisms.

The same protocol applied for bacterial cells was also carried out for *Candida* strains. However, a two‐fold dilution was made using a lower concentration of *F. clypeata* essential oil, and sabouraud broth was used instead of Muller hinton broth throughout the study. Four microlitre of essential oil was dissolved in 200 μL of DMSO. Fifty microlitre of this solution was added to 150 μL of sabouraud broth in the first wells in the first row of the microplate, and two‐fold dilutions were made from the 1st to the 10th well. *F. clypeata* essential oil concentration in the wells ranged from 5 to 0.009 μL/mL. Samples were prepared from each yeast strain, including *C. albicans* , *C. krusei* , *C. parapsilosis* , and *C. tropicalis* , at a density of 0.5 McFarland (1–1.5 × 10^6^ cfu/mL). Subsequently, 1 μL of these samples was inoculated into wells 1 to 10 of A, B, C, and D rows on the microplate. The whole experiment was performed in triplicate, with zero standard deviation.

##### 2.3.2.2 Agar Dilution Assays

In the agar dilution methods, 90 μL of essential oil was dissolved in 1.2 mL of DMSO. This solution was added to 4.8 Muller hinton agar, and two‐fold dilutions were made from the 1st to the 11th plate. *F. clypeata* essential oil concentration in the plates ranged from 15 to 0.015 μL/mL. Bacteria standard inoculums were inoculated in the areas indicated on the agar. The same method was also used for antifungal activity at different concentrations. 24 μL of essential oil was dissolved in 1.2 mL of DMSO. This mixture was added to 4.8 sabouraud agar, and two‐fold dilutions were made from the 1st to the 11th plate. *F. clypeata* essential oil concentration in the plates ranged between 15 to 0.015 μL/mL. The 12th plate demonstrates the viability of microorganisms and contains a pure agar medium. Afterward, standard inoculums of *Candida* species were inoculated separately in regions A, B, C and D. All plates were incubated at 36°C overnight and observed for growth.

### 2.4 Anticholinesterase Activity Determination Method

The original assay reported by Ellman was slightly modified and applied to the current study (Ellman et al. 1961). The acetylthiocholine iodide (Sigma‐B3253) and butyrylthiocholine iodide (Sigma‐A7000) substrates were used for the inhibition of AChE (Sigma‐C3389) and BChE (Sigma‐C4290) enzymes. The potential of essential oil was compared with standard Galanthamine. The buffer (Na_3_PO_4_ pH 8.0), 4 mM essential oil, and corresponding enzyme AChE (or BChE) were incubated for 15 min at 25°C. The addition of DTNB and the corresponding substrate initiated the reaction. The microplate ELISA reader XS was used to monitor the hydrolysis of the substrates. The wavelength of 412 nm was selected to measure the absorbance values. DMSO was used as a solvent to dissolve the samples and controls.

$$\%\text{Inhibition}=\left(A_{0}–A_{1}\right)/A_{0}\times 100$$

where *A*_0_ is the absorbance of the control, and *A*_1_ is the absorbance in the presence of the sample.

### 2.5 Statistical Analysis

The results were mean ± SD of three parallel measurements. All statistical comparisons were carried out using Student's *t*‐test; *p* values < 0.05 were considered significant.

### 2.6 α‐Glucosidase Inhibition Assay

The α‐glucosidase inhibitory activity was performed with the described method by Schmidt et al. with minor changes (Schmidt et al. 2012). The mixture of buffer (phosphate, pH 7.5, 0.02% NaN_3_), essential oil in DMSO, and the enzyme solution was prepared in each well and incubated at 28°C for 10 min before adding substrate (PNPG) to a final volume of 200 μL. The wavelength of 405 nm was chosen to measure absorbance every 40 s for 35 min. The α‐glucosidase inhibitory activity (%) was calculated using the following formula.

$$\%\text{inhibition}=\left({\text{Slope}}_{\text{blank}}–{\text{Slope}}_{\text{sample}}\right)/{\text{Slope}}_{\text{blank}}\times 100$$

Three replicates were performed for all measurements (Student's *t*‐test *p* < 0.05). IC_50_ calculations were performed with GraphPad Prism 8.0.1. Acarbose was used positive control.

### 2.7 Antioxidant Activity

#### 2.7.1 DPPH Free Radical Scavenging Activity Assay

The DPPH radical scavenging activity assay has been performed according to the method of Blois et al. with minor modifications (Blois 1958). Butylated Hydroxy Toluene (BHT), Butylated Hydroxy Anisole (BHA), and α‐TOC (Tocopherol) were also assayed as standards for comparison.

#### 2.7.2 ABTS Cation Radical Decolorization Activity

The procedure was followed from the literature (Miller et al. 1993) and the measurements at 734 nm were read for each concentration relative to a blank absorbance (DMSO). BHA, BHT, and ‐TOC were used as standards. The obtained values were compared to the standards.

#### 2.7.3 CUPRAC Cupric Ion‐Reducing Antioxidant Capacity

The antioxidant capacity assay to identify the reductive efficiencies of the essential oil for Cu metal was performed according to the method reported in the literature (Apak et al. 2005). The absorbances were measured at 450 nm after 30 min. BHA, BHT, and α‐TOC were used as standards.

### 2.8 Molecular Docking Analysis

The compounds detected in the essential oil of *F. clypeata* were plotted to get SMILES from PubChem (https://pubchem.ncbi.nlm.nih.gov). Drawings and energy minimization of these ligands were performed with the ChemOffice program. To evaluate 1HSK‐antimicrobial activity and to evaluate 1EA1‐antifungal activity, molecular docking studies were performed using the standard procedure to determine the binding modes of the compounds detected in the essential oil of *F. clypeata* at the active sites of the macromolecules (Benson et al. 2001; Podust et al. 2001). The macromolecules crystal structures were retrieved from the Protein Data Bank server (https://www.rcsb.org/, accessed 5 May 2025) and optimized with Schrödinger Maestro. Molecular docking was performed with both Autodock (Sanner 1999) and Vina (Trott and Olson 2010) programs. For 1HSK, since we had worked with this macromolecule before, FAD was re‐docked into the target site of the macromolecule to verify the docking program, and the RMSD value was found to be less than two (< 2). For 1EA1, TPF (Fluconazole) was re‐docked into the target site of the macromolecule to verify the docking program, and the RMSD value was found to be less than one (< 1). The results were visualized using the Maestro program (Maestro, Schrödinger, LLC 2024).

### 2.9 ADME Predictions

The SwissADME online page was used to calculate the physicochemical and pharmacokinetic properties of compounds detected in the essential oil of *F. clypeata* obtained (**1–19**) and to compare the compounds among themselves based on the results seen (http://www.swissadme.ch/, access date: 08.03.2025) (Daina et al. 2014, 2017; Daina and Zoete 2016).

## 3 Results

### 3.1 Results of GS‐MS Analysis

According to the results of the applied Gas Chromatography‐Mass Spectrometry (GC‐MS) analysis, 19 different compounds were determined, constituting a total of 99.76% of the plant essential oil (Table 1). Five of these compounds constitute between 73.13% and 0.50% of the total content. Nine compounds comprise between 0.50% and 0.10% of the content. The remaining six compounds are below 0.10% of the total content. The most dominant compounds in the content are dimethyl disulfide, with 73.13%, and dimethyl trisulfide, with 19.87%. These are followed by 2,3,5‐trithiahexane 5‐oxide, 2,4,5‐trithiahexane 2,2‐dioxide, and 2‐methyl‐3‐phenylpropanal, with rates of 2.75%, 1.60%, and 0.69%, respectively.

**TABLE 1:** Chemical compositions of the essential oil of *F. clypeata* .

Compounds		Rt (min)	%
**1**	Dimethyl disulfide	4.48	73.13
**2**	Dimethyl trisulfide	10.27	19.87
**3**	Methyl methanethiosulfinate	10.76	0.25
**4**	2,6‐dimethyl‐3‐ethylpyrazine	14.22	0.13
**5**	1,2,4‐trithiolane	14.70	0.11
**6**	Linalool	14.92	0.03
**7**	2,3,5‐trithiahexane	15.92	0.22
**8**	Endo‐borneol	17.37	0.08
**9**	α‐terpineol	18.24	0.01
**10**	Estragole	18.49	0.15
**11**	Dimethyl tetrasulfide	19.13	0.11
**12**	2‐methyl‐3‐phenylpropanal	19.96	0.69
**13**	Anisaldehyde	20.46	0.16
**14**	Cis‐anethole	21.49	0.38
**15**	1‐(2,6,6‐trimethyl‐1,3‐cyclohexadien‐1‐yl) ethanol	21.68	0.02
**16**	2,6,11‐trimethyldodecane	21.87	0.05
**17**	2,3,5‐trithiahexane 5‐oxide	25.21	2.75
**18**	2,4,5‐trithiahexane 2,2‐dioxide	26.58	1.60
**19**	2,4,5,7‐tetrathiaoctane 2‐oxide	36.88	0.02
	Total		99.76

Abbreviation: Rt, retention time.

### 3.2 Antimicrobial Activity Results

#### 3.2.1 Antibacterial Activity

**FIGURE 2:**
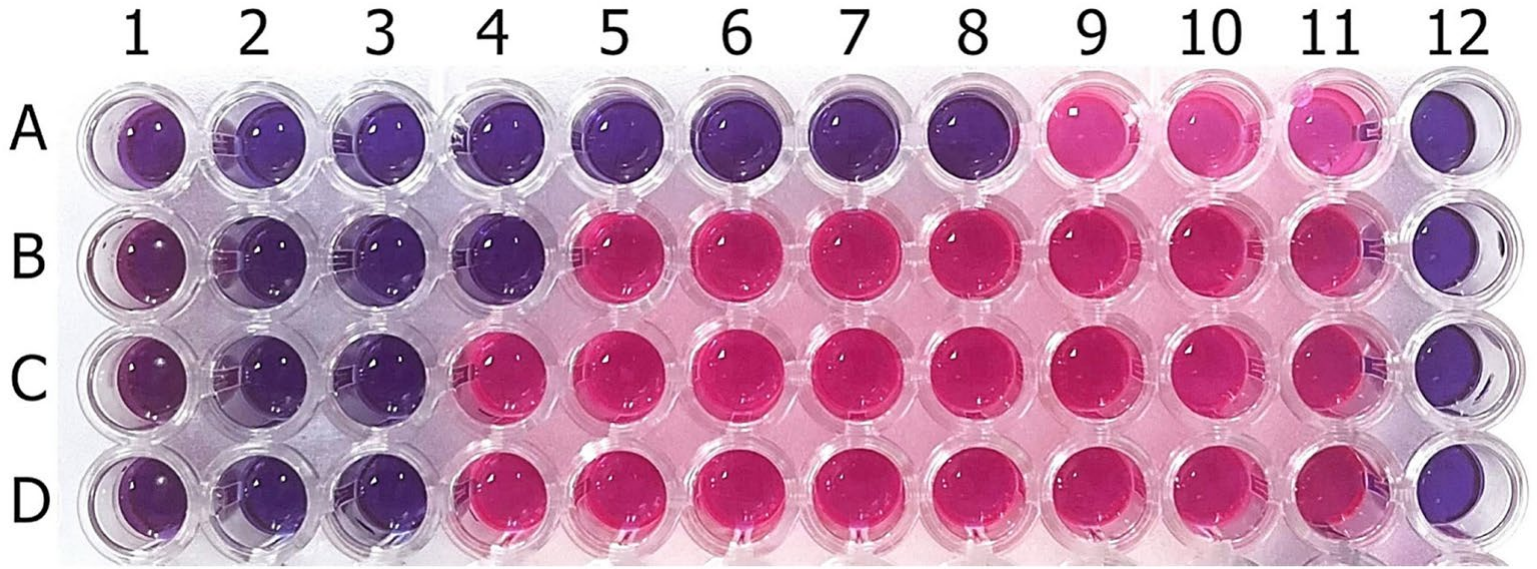
The microplate picture shows the antibacterial test results of *F. clypeata* essential oil against *S. aureus* (A) (MIC: 0.117 μL/mL), *E. aerogenes* (B) (MIC: 1.875 μL/mL), *E. coli* (C) (MIC: 3.75 μL/mL), and *K. pneumoniae* (D) (MIC: 3.75 μL/mL). 1st to 10th wells include the different concentrations of *F. clypeata* essential oil: 15, 7.5, 3.75, 1.875, 0.938, 0.469, 0.234, 0.117, 0.059 and 0.029 μL/mL, respectively. 11th and 12th wells are positive and negative controls, respectively.

**FIGURE 3:**
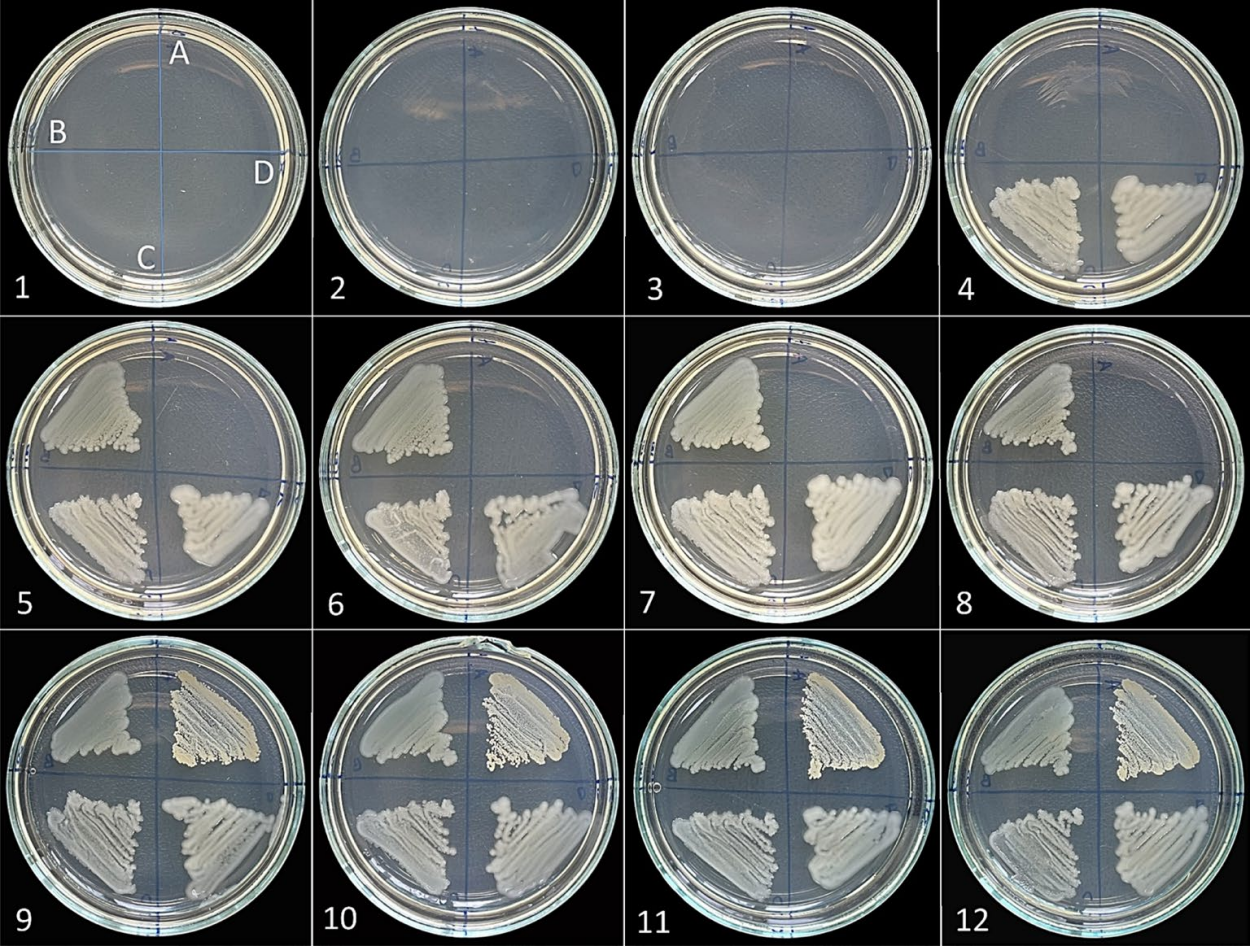
The plates show pictures of antibacterial test results of *F. clypeata* essential oil against *S. aureus* (A), *E. aerogenes* (B), *E. coli* (C), and *K. pneumoniae* (D). 1st to 11th plates include the different concentrations of *F. clypeata* essential oil: 15, 7.5, 3.75, 1.875, 0.938, 0.469, 0.234, 0.117, 0.059, 0.029 and 0.015 μL/mL, respectively. The 12th plate is the positive control.

The antibacterial activity test results demonstrated that *F. clypeata essential oil* has a strong inhibitory effect on the growth of bacteria (Figures 2 and 3). In the broth dilution method, among the bacteria used in the activity tests, it was determined that the highest inhibition was against *S. aureus* , with the lowest MIC value. The MIC value in the eighth well (0.117 μL/mL), where the lowest concentration of *F. clypeata* essential oil was used and no growth occurred (no color change), is the MIC value of *F. clypeata essential oil* against *S. aureus* . The MIC values of *the essential oil* against *E. aerogenes* , *E. coli* , and *K. pneumoniae* were found to be 1.875, 3.75, and 3.75 μL/mL, respectively.

The agar dilution results were the same as the broth dilution results, and the MIC values of the essential oil used against bacterial strains were determined to be the same. Therefore, *S. aureus* inoculated into region A on the plate started to grow from the 9th plate, and *E. aerogenes* , *E. coli* , and *K. pneumoniae* started to grow from the 5th, 4th and 4th plates, respectively (Figure 3).

#### 3.2.2 Antifungal Activity

**FIGURE 4:**
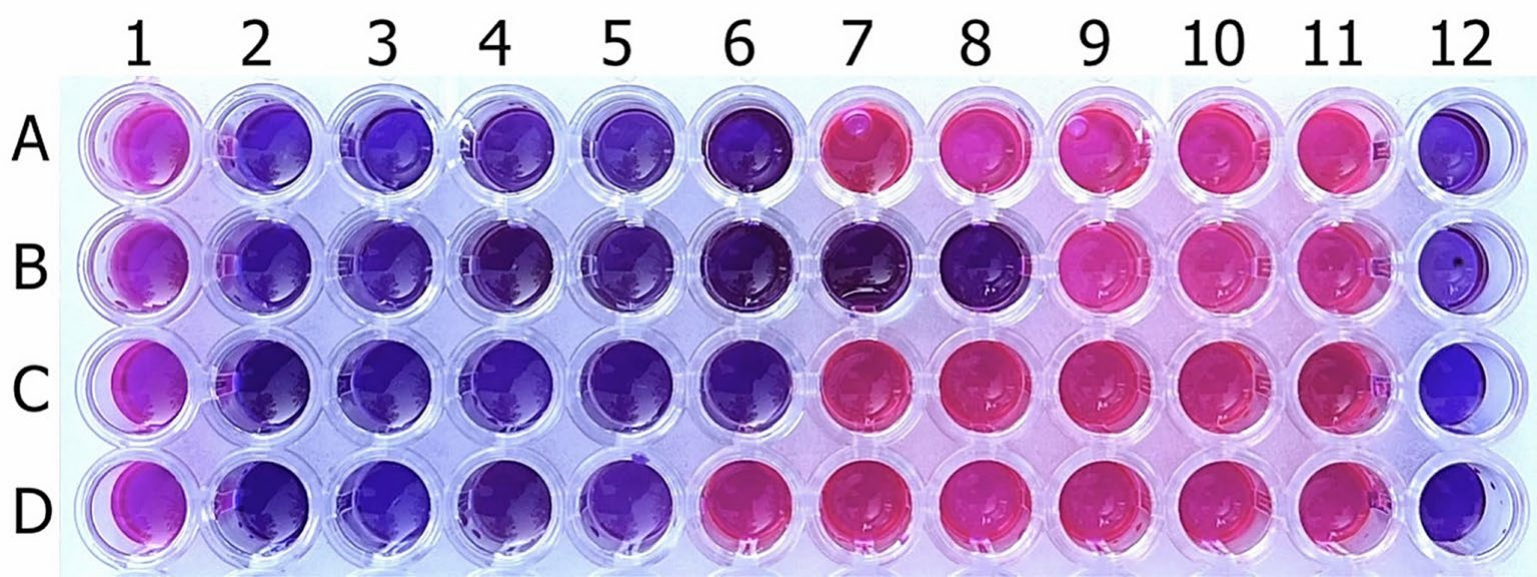
The microplate picture shows antifungal test results of *F. clypeata essential oil* against *C. albicans* (A) (MIC: 0.156 μL/mL), *C. parapsilosis* (B) (MIC: 0.039 μL/mL), *C. krusei* (C) (MIC: 0.156 μL/mL), and *C. tropicalis* (D) (MIC: 0.312 μL/mL). 1st to 10th wells include the different concentrations of *F. clypeata* essential oil: 5, 2.5, 1.25, 0.625, 0.312, 0.156, 0.078, 0.039, 0.019 and 0.009 μL/mL, respectively. 11th and 12th wells are positive and negative controls, respectively.

*F. clypeata essential oil* concentrations used in the antibacterial test were studied in the antifungal assay. However, since the inhibitory effect of *the essential oil* was observed to be higher against yeast species than that of bacterial strains, the antifungal activity test was carried out with lower *F. clypeata essential oil* concentrations. Figure 4 shows the antifungal test results using the microdilution method. According to these results, the MIC values of plant essential oil against *C. albicans* , *C. parapsilosis* , *C. krusei* , and *C. tropicalis* were 0.156, 0.039, 0.156, 0.312 μL/mL in the 6th, 8th, 6th and 5th wells, respectively, where there was no color change and the lowest *F. clypeata essential oil* concentrations were used.

**FIGURE 5:**
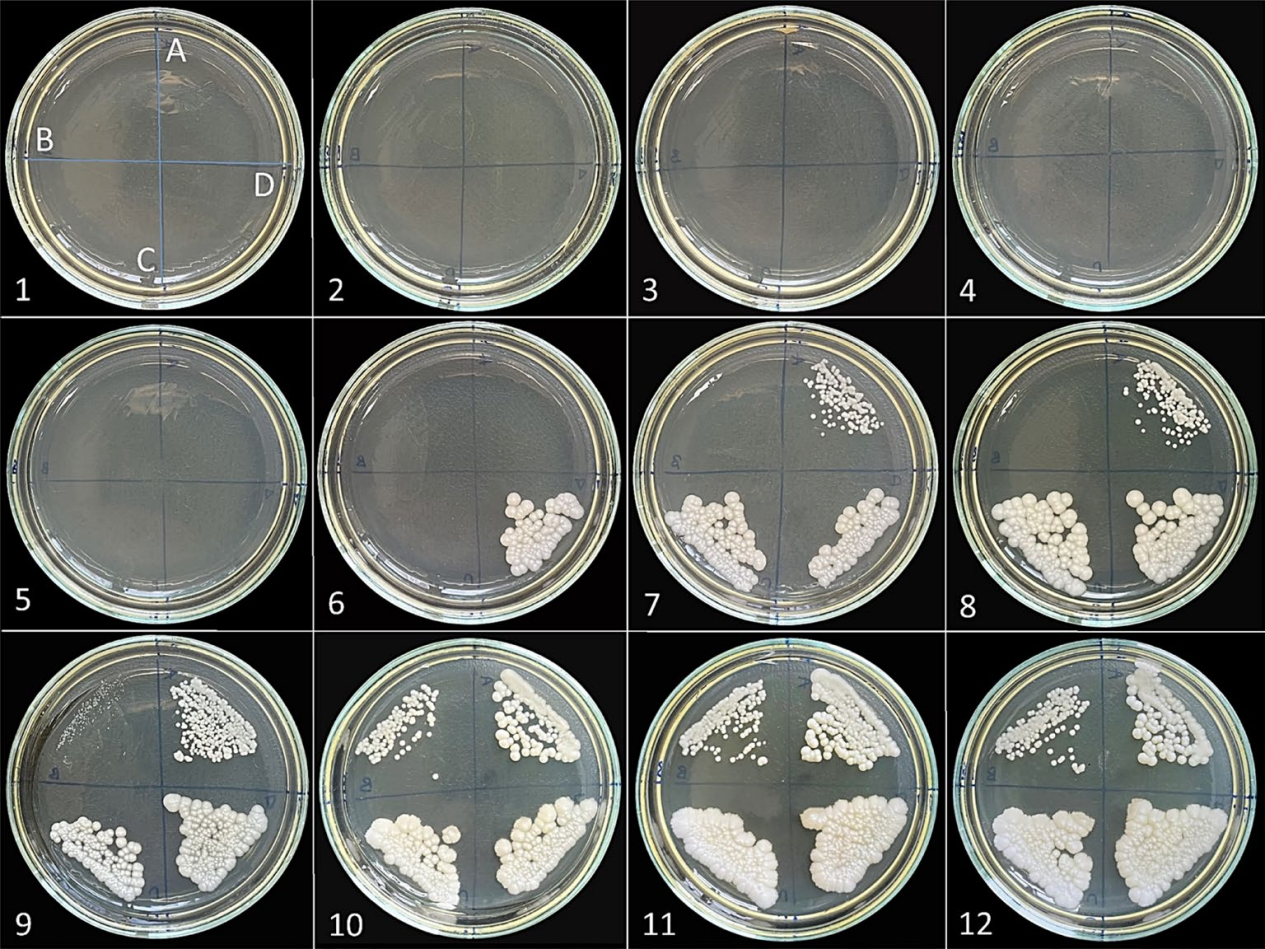
The plates show pictures of antifungal test results of *F. clypeata essential oil* against *C. albicans* (A), *C. parapsilosis* (B), *C. krusei* (C), and *C. tropicalis* (D). 1st to 11th plates include the different concentrations of *F. clypeata* essential oil: 15, 7.5, 3.75, 1.875, 0.938, 0.469, 0.234, 0.117, 0.059, 0.029, and 0.015 μL/mL, respectively. The 12th plate is the positive control.

**FIGURE 6:**
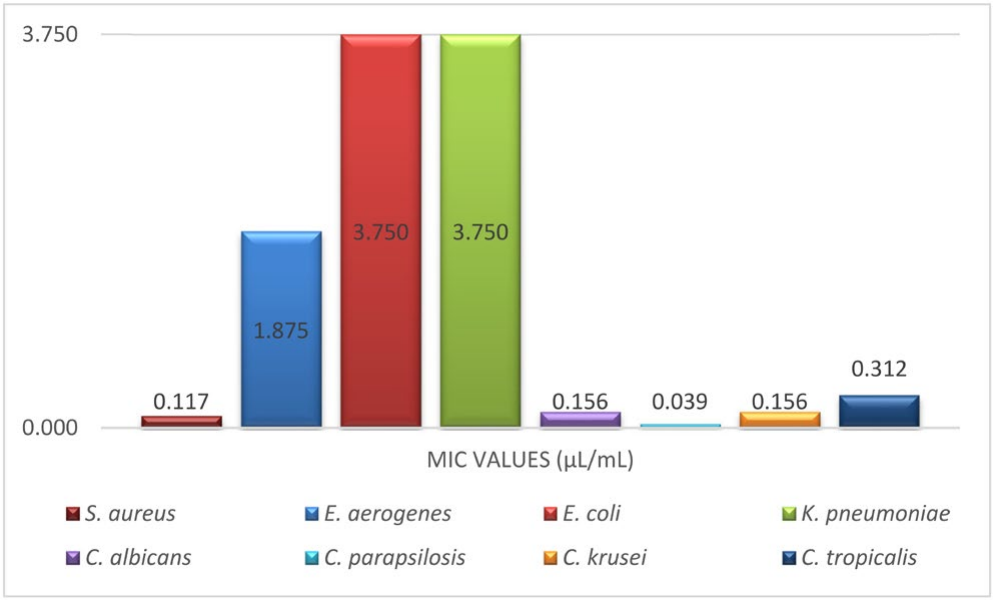
MIC values of *F. clypeata* essential oil against different bacterial and fungal species.

The agar dilution results are shown in Figure 5. The results obtained were the same as those obtained using the broth dilution method. *C. albicans* , *C. parapsilosis* , *C. krusei* , and *C. tropicalis* started to grow after the 7th, 9th, 7th, and 6th plates, respectively. Therefore, MIC values against the tested yeast species, including *C. albicans* , *C. parapsilosis* , *C. krusei* , and *C. tropicalis* , were found to be 0.156, 0.039, 0.156, and 0.312 μL/mL, respectively. All MIC values are given comparatively in Figure 6.

### 3.3 Determination of Enzyme Inhibition Potential of Essential Oil

#### 3.3.1 Anticholinesterase Activity

The result for the inhibition potential of the essential oil has been given as an IC_50_ value and compared with Galanthamine as the standard. The potency of the sample for anticholinesterase activity was determined with the lower inhibition IC_50_ value obtained from the assay. The positive control, Galanthamine, provided 3.12 μg/mL and 6.42 μg/mL IC_50_ concentrations for both AChE and BChE inhibitions, respectively. The data demonstrated that the essential oil was sensitive to BChE and AChE enzyme inhibitions (Table 2). More importantly, selective inhibition in favor of the BChE enzyme was detected with a value of 4.78 μg/mL IC_50_ value and found to be comparable to the standard. The 50% AChE inhibition was detected at a concentration of 17.31 μg/mL.

**TABLE 2:** Enzyme inhibition profiles of *F. clypeata essential oil*.

Compounds	AChE	BChE	α‐glucosidase
	IC_50_ μg/mL		% inhibition (20 μg/mL)
*F. clypeata essential oil*	17.31 ± 0.02	4.78 ± 65	5.01 ± 3.08
Galanthamin^a^	3.12 ± 0.01	6.42 ± 0.14	—
Acarbose^b^	—	—	1033.8 ± 3.4^c^

^a^ Positive control for anticholinesterase activity.

^b^ Positive for anti‐diabetes activity.

^c^ (IC_50_ μM).

#### 3.3.2 Anti‐Diabetes Activity

The determination of anti‐diabetes activity was investigated by the treatment of essential oil with the α‐glucosidase enzyme. The inhibition potential was compared with Acarbose as the standard. The potent drug molecule acarbose, used as a positive control, provided an IC_50_ value of 1033.8 μM for α‐glucosidase enzyme inhibition. The assay was carried out at (20 μg/mL) concentration; however, the obtained data was found to be non‐active with a 5.01% inhibition value (Table 2).

### 3.4 Antioxidant Results

The DPPH antioxidant assay consists of the reduction process of DPPH to DPPH2 due to the donation of a hydrogen atom (or one‐electron) by the scavenging molecule to DPPH, and the measurements provide the free radical potency of the targeted molecule. The cationic radical‐reducing ability of the essential oil was investigated by the ABTS^•+^ assay. The reductive efficiency of the targeted oil for Cu metal was evaluated by the Cupric Ion‐Reducing Antioxidant Capacity method and based on the reaction between the investigated compound and CuCl_2_, neocuproine, as well as ammonium acetate at pH 7. Three replicates were carried out to calculate the mean and standard deviation (SD) values. The DPPH results were given in Table 3 as IC_50_ concentration values of the essential oil *F. clypeata* and the positive controls, BHT, BHA, and α‐TOC. The results indicated that the candidate was more efficient for DPPH activity than the standards BHA and BHT, and comparable efficiency was detected with the standard α‐TOC. The 1.54 (μg/mL) concentration was found to be promising for the free radical scavenging activity. However, the ABTS Cationic Radical inhibition was detected as the least potent for the biological aspects of the essential oil (Table 3). The compound reflected the 50% inhibition at 10.32 (μg/mL) concentration, and the value was found to be comparable with the α‐TOC standard. More importantly, the reduction of copper metal, CUPRAC assay, with the essential oil was the most promising antioxidant assay (Table 3). The IC_50_ value revealed that the efficiency was better than all the standards tested in the assay.

**TABLE 3:** Antioxidant activity results of *F. clypeata* essential oil.*

Samples	IC_50_ values (μg/mL)^a^	*A*_0.5_ values (μg/mL)^b^	
	DPPH free radical	ABTS cation radical	CUPRAC
*F. clypeata* essential oil	1.54 ± 0.01	10.32 ± 0.28	3.72 ± 0.09
BHA^c^	3.22 ± 0.08	2.74 ± 0.03	4.14 ± 0.17
α‐TOC^c^	1.41 ± 0.04	8.48 ± 0.43	13.64 ± 0.32
BHT^c^	16.71 ± 0.80	4.44 ± 0.30	3.93 ± 0.24

^a^ Values were given as IC_50_ for DPPH free and ABTS cation radical scavenging activities.

^b^ Values were given as *A*_0.5_ for CUPRAC activity.

^c^ Standard compounds.

* Values expressed are means ± standard deviation of three parallel measurements (*p* < 0.05).

### 3.5 Molecular Docking Studies

**FIGURE 7:**
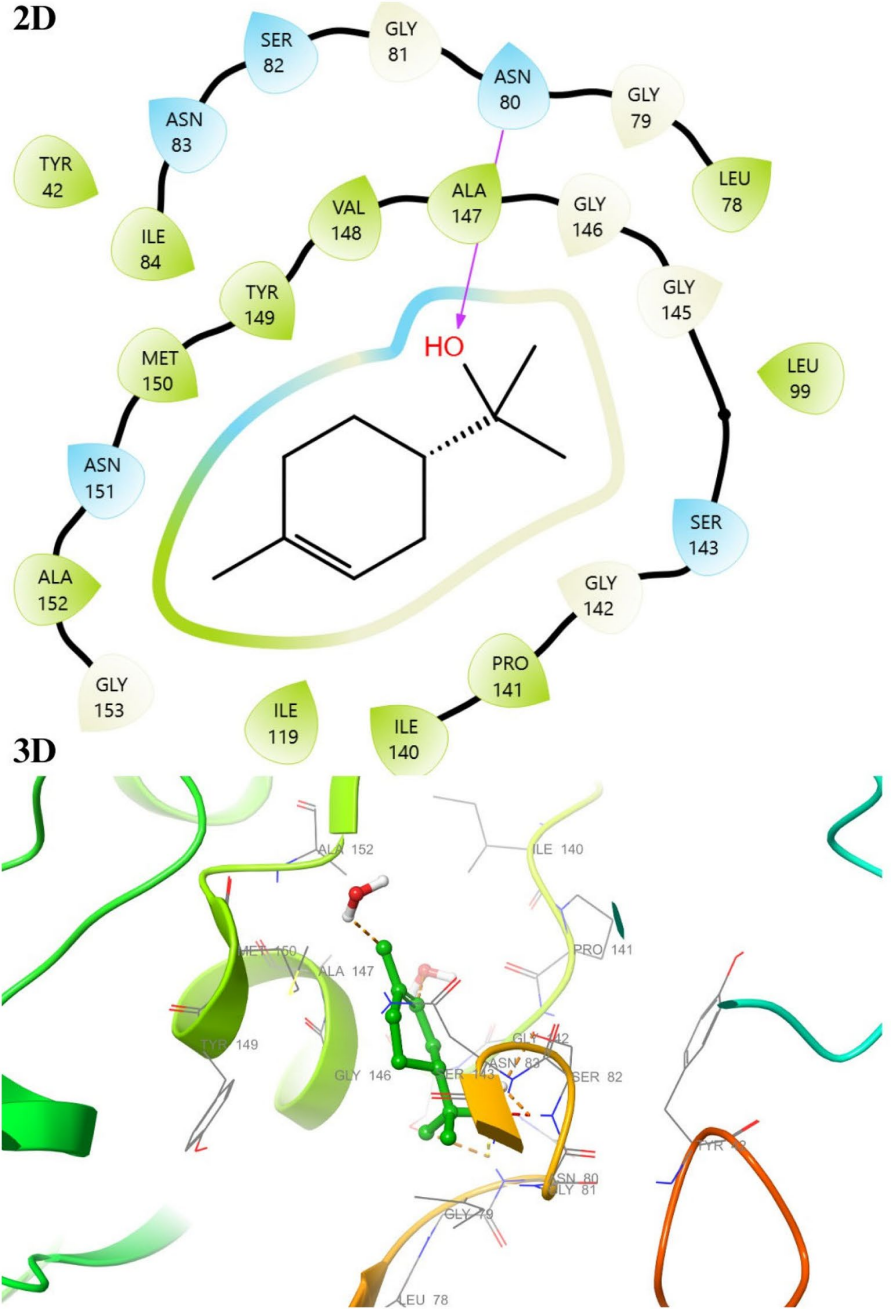
2D and 3D interaction diagram with 1HSK for α‐Terpineol (**9**).

**FIGURE 8:**
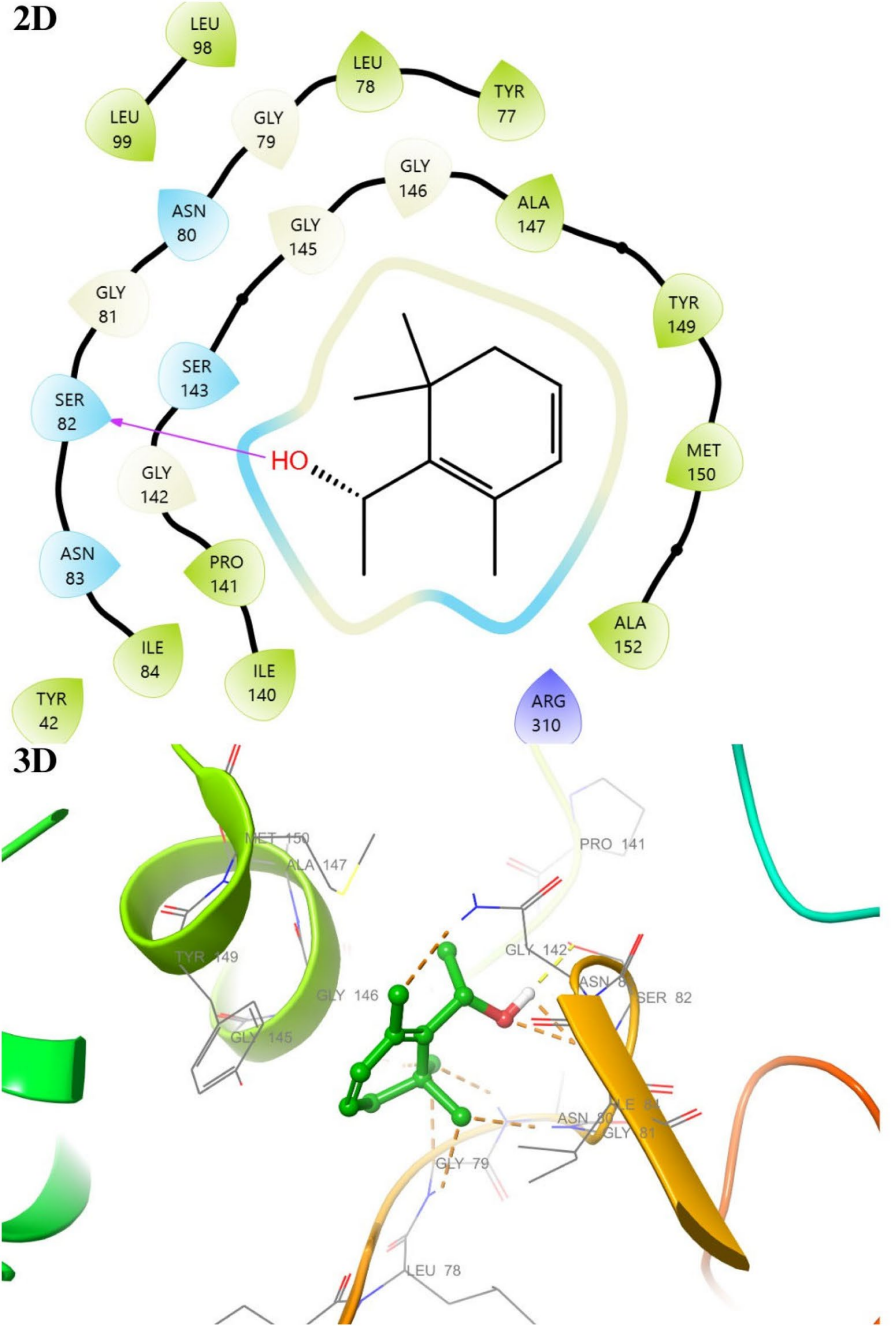
2D and 3D interaction diagram with 1HSK for 1‐(2,6,6‐Trimethyl‐1,3‐cyclohexadien‐1‐yl)ethanol (**15**).

The active binding sites of the receptor 1HSK have been previously calculated or determined in the protein database (Benson et al. 2001). In light of this data, docking studies were performed to see the interaction modes of all compounds constituting the essential oil of *F. clypeata* with the active site of the macromolecule. Binding types and associated residues were generated in detail by Maestro Software (Tables 4 and 5, Figures 7 and 8, File S1–S46). Some residues previously identified as important for the interaction between *S. aureus* Murb and its cofactor FAD were described in detail in our previous study (Unver, Uslu, et al. 2024). The interaction modes with 1HSK for compounds **8**, **9**, **15**, and **16** were visualized in 2D and 3D with the Maestro program.

**TABLE 4:** Chemical contents of *F. clypeata* essential oil and 1HSK with molecular docking scores and estimated inhibition constants.

Comp.	Autodock results		Vina
	Estimated inhibition constant, Ki	Best docking score	Best docking score
**1**	9.56 mM	−2.76	−2.4
**2**	5.08 mM	−3.13	−2.7
**3**	236.23 μM	−4.95	−4.2
**4**	185.78 μM	−5.09	−5.8
**5**	2.94 mM	−3.45	−2.9
**6**	157.33 μM	−5.19	−6.3
**7**	5.06 mM	−3.13	−3.0
**8**	48.7 μM	−5.89	−6.3
**9**	39.84 μM	−6.00	−6.6
**10**	227.50 μM	−4.97	−6.4
**11**	2.62 mM	−3.52	−3.0
**12**	79.27 μM	−5.59	−6.7
**13**	157.53 μM	−5.19	−6.2
**14**	146.20 μM	−5.23	−6.3
**15**	42.03 μM	−5.97	−6.3
**16**	57.10 μM	−5.79	−6.5
**17**	467.37 μM	−4.54	−3.9
**18**	128.32 μM	−5.31	−4.7
**19**	222.66 μM	−4.98	−4.3

Abbreviations: μM, micromolar; docking score, estimated free energy of binding (kcal/mol); mM, milimolar.

**TABLE 5:** Residues and bond types all components of *F. clypeata* essential oil with 1HSK.

Comp.	Autodock results	
	Interacting residues	Interaction types
**1**	—	—
**2**	—	—
**3**	SER82	Hydrogen bond
	SER143	Hydrogen bond
**4**	HOH684	Hydrogen bond
**5**	—	—
**6**	ASN80	Hydrogen bond
**7**	—	—
**8**	VAL199	Hydrogen bond
**9**	ASN80	Hydrogen bond
**10**	GLY81	Hydrogen bond
**11**	—	—
**12**	ASN80	Hydrogen bond
	SER143	Hydrogen bond
	TYR149	Pi‐pi stacking
**13**	ASN80	Hydrogen bond
	ASN83	Hydrogen bond
	SER143	Hydrogen bond
**14**	GLY81	Hydrogen bond
**15**	SER82	Hydrogen bond
**16**	—	—
**17**	ASN80	Hydrogen bond
	SER143	Hydrogen bond
**18**	ASN80	Hydrogen bond
	ASN82	Hydrogen bond
	SER143	Hydrogen bond
**19**	ASN80	Hydrogen bond
	SER143	Hydrogen bond

**FIGURE 9:**
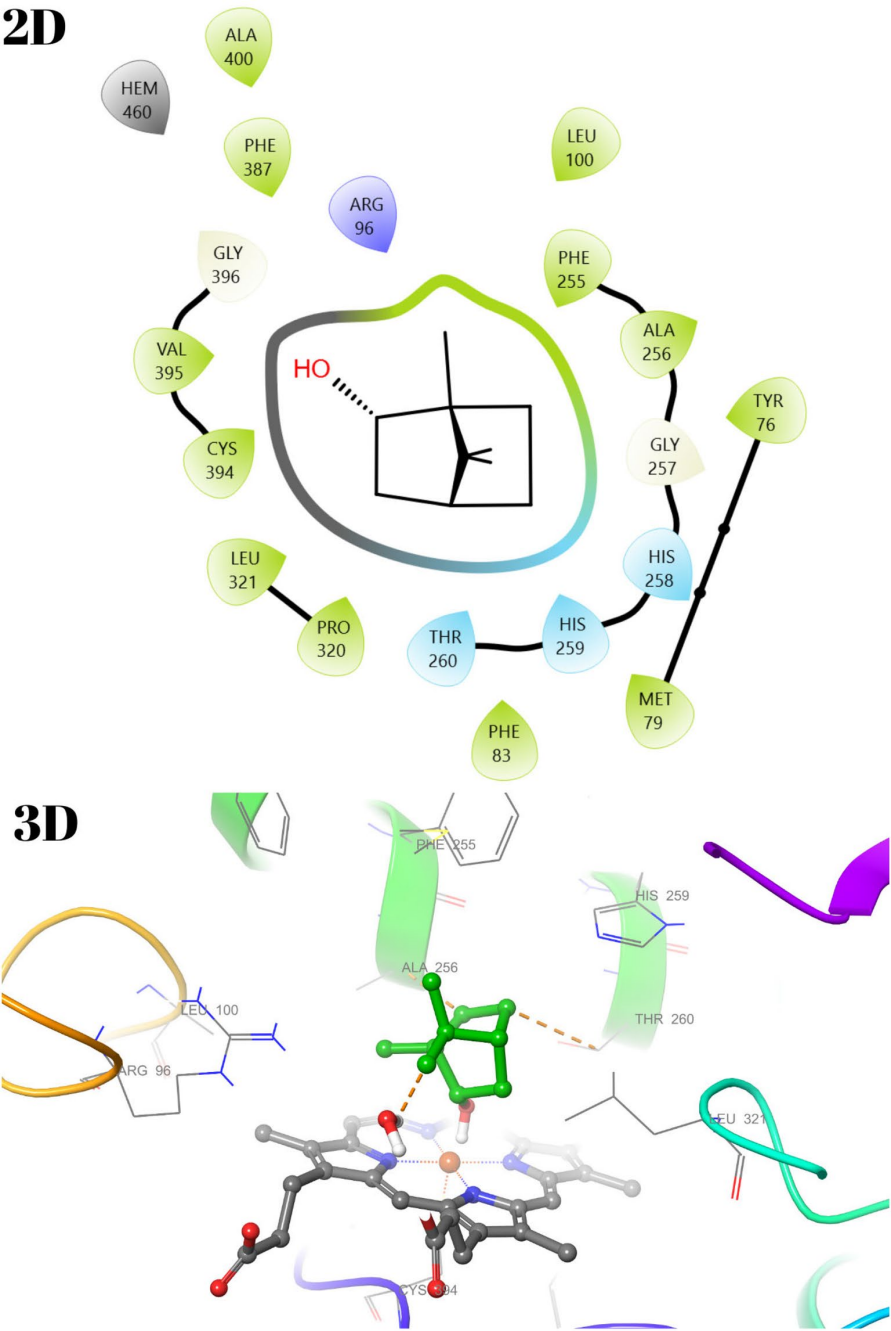
2D and 3D interaction diagram with 1EA1 for Endo‐Borneol (**8**).

**FIGURE 10:**
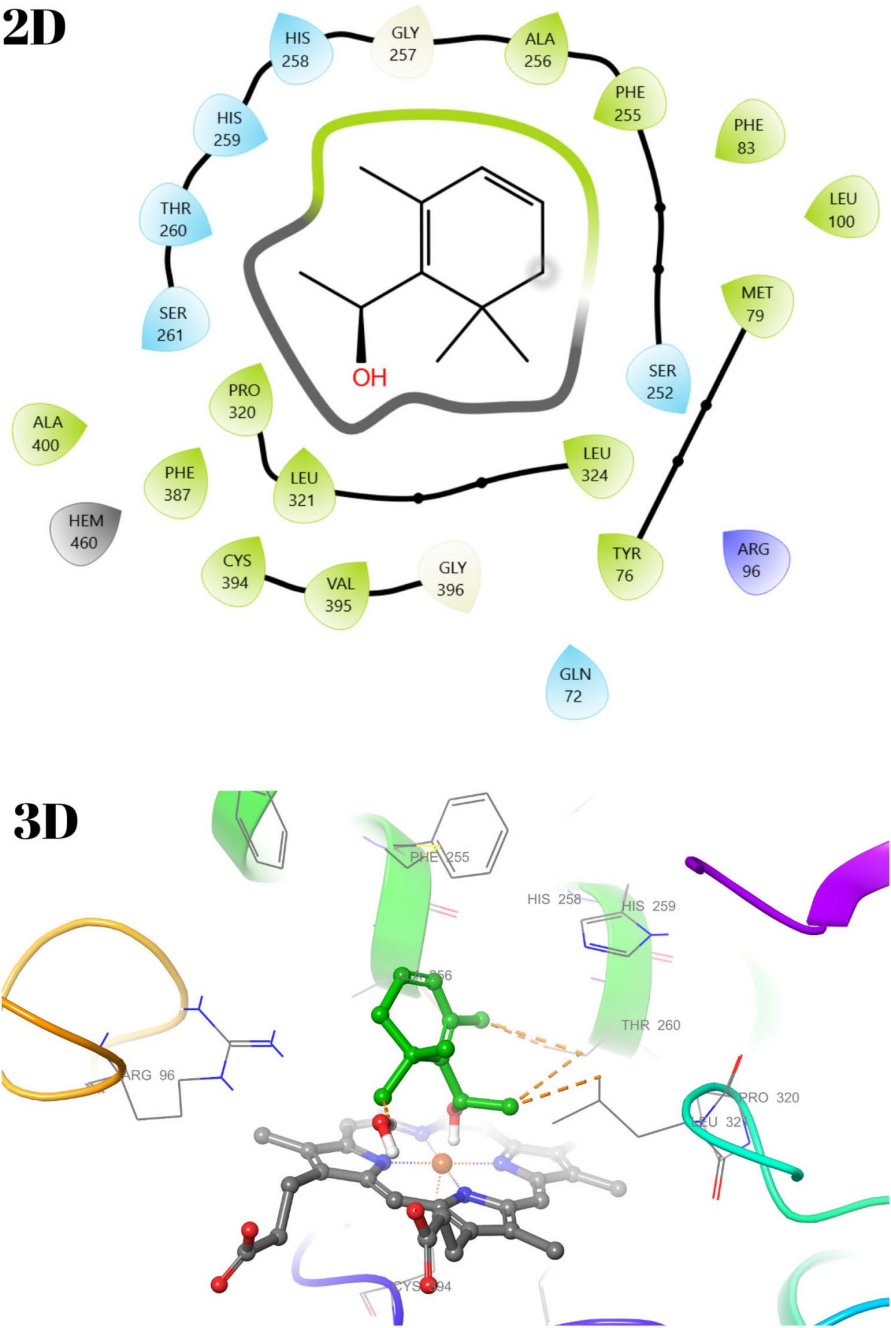
2D and 3D interaction diagram with 1EA1 for 1‐(2,6,6‐Trimethyl‐1,3‐cyclohexadien‐1‐yl)ethanol (**15**).

The active binding sites of the receptor 1EA1 have been previously calculated or determined in the protein database (Benson et al. 2001). In light of this data, docking studies were performed to see the interaction modes of all compounds constituting the essential oil of *F. clypeata* with the active site of the macromolecule. Binding types and associated residues were generated in detail by Maestro Software (Table 6, Figures 9 and 10). Some residues previously identified as important for the interaction between *Mycobacterium tuberculosis* and its cofactor HEM were described in detail in our previous study (Unver, Uslu, et al. 2024). The interaction modes with 1EA1 for compounds **8**, **9**, **15**, and **16** were visualized in 2D and 3D with the Maestro program. The residues and bond types that the components with high docking scores of *F. clypeata* essential oil interact with 1EA1 were examined, and no strong interaction, such as hydrogen bonding, was found in any of them (Figures 9 and 10).

**TABLE 6:** Molecular docking scores and estimated inhibition constants of some chemical constituents of *F. clypeata* essential oil.

Comp.	Autodock results		Vina
	Estimated inhibition constant, Ki	Best docking score	Best docking score
**8**	1.63 μM	−7.89	−6.7
**9**	26.42 μM	−6.25	−6.7
**15**	3.55 μM	−7.43	−6.5
**16**	12.51 μM	−6.69	−6.8

Abbreviations: μM, micromolar; docking score, estimated free energy of binding (kcal/mol).

### 3.6 ADME Predictions

SMILES data of all compounds constituting the essential oil of *F. clypeata* obtained were used in the ADME program. When the LogS values, which are an indicator of how much chemical compounds are soluble in water, were examined, it was seen that the solubilities of compounds **1**–**19** were between −0.58 and −5.27, and their dissolution rates were between slightly soluble and soluble. cLogP values, which express the fat solubility criteria of the compounds, vary between 0.19 and 5.75. The observation that the F value showing the oral bioavailability score is 0.55 for all compounds shows that the compounds are positive regarding bioavailability (Tables 7 and 8).

**TABLE 7:** Druglikeness, water solubility, and pharmacokinetic properties of all compounds constituting the essential oil of *F. clypeata* .

Comp.	Druglikeness					Water solubility		Pharmacokinetics	
	Lipinski	Ghose	Veber	Egan	Muegge	LogS	Class	GI abs.	*F*
**1**	+	−	+	+	−	−1.47	Very soluble	High	0.55
**2**	+	−	+	+	−	−1.34	Very soluble	High	0.55
**3**	+	−	+	+	−	−0.58	Very soluble	High	0.55
**4**	+	−	+	+	−	−2.02	Soluble	High	0.55
**5**	+	−	+	+	−	−1.57	Soluble	High	0.55
**6**	+	−	+	+	−	−2.40	Soluble	High	0.55
**7**	+	−	+	+	−	−1.56	Very soluble	High	0.55
**8**	+	−	+	+	−	−2.51	Soluble	High	0.55
**9**	+	−	+	+	−	−2.87	Soluble	High	0.55
**10**	+	−	+	+	−	−3.09	Soluble	High	0.55
**11**	+	−	+	+	−	−1.75	Very soluble	High	0.55
**12**	+	−	+	+	−	−2.34	Soluble	High	0.55
**13**	+	−	+	+	−	−2.10	Soluble	High	0.55
**14**	+	−	+	+	−	−3.11	Soluble	High	0.55
**15**	+	+	+	+	−	−2.08	Soluble	High	0.55
**16**	+	−	+	+	−	−5.27	Moderately	High	0.55
**17**	+	−	+	+	−	−0.67	Very soluble	High	0.55
**18**	+	−	+	+	−	−0.95	Very soluble	High	0.55
**19**	+	−	+	+	−	−1.30	Very soluble	High	0.55

*Note:* LogS: ESOL. Class: −6 < Moderately < −4.

Abbreviations: Comp, compounds; *F*, bioavailability score; GI abs, gastrointestinal absorption.

**TABLE 8:** The physicochemical and lipophilicity properties of all compounds constituting the essential oil of *F. clypeata* .

Comp.	Physicochemical properties							Lipophilicity
	MW	Fsp3	RB	HBA	HBD	MR	TPSA	cLogP
**1**	94.20	1.00	1	0	0	26.91	50.60	1.34
**2**	126.26	1.00	2	0	0	34.50	75.90	1.51
**3**	126.20	1.00	1	2	0	28.28	67.82	0.19
**4**	136.19	0.50	1	2	0	41.74	25.78	1.64
**5**	124.25	1.00	0	0	0	32.39	75.90	1.69
**6**	154.25	0.60	4	1	1	50.44	20.23	2.66
**7**	140.29	1.00	3	0	0	39.31	75.90	1.72
**8**	154.25	1.00	0	1	1	46.60	20.23	2.38
**9**	154.25	0.80	1	1	1	48.80	20.23	2.58
**10**	148.20	0.20	3	1	0	47.04	9.23	2.78
**11**	158.33	1.00	3	0	0	42.09	101.20	1.85
**12**	148.20	0.30	3	1	0	46.03	17.07	2.26
**13**	136.15	0.12	2	2	0	38.32	26.30	1.61
**14**	148.20	0.20	2	1	0	47.83	9.23	2.78
**15**	166.26	0.64	1	1	1	52.83	20.23	2.46
**16**	212.41	1.00	9	0	0	74.22	0.00	5.75
**17**	156.29	1.00	3	1	0	39.99	86.88	0.82
**18**	172.29	1.00	3	2	0	40.68	93.12	0.79
**19**	202.38	1.00	5	1	0	52.39	112.18	1.40

Abbreviations: Fsp3, fraction Csp3; HBA, number of hydrogen bond acceptors; HBD, number of hydrogen bond donors; MR, molar refractivity; MW, molecular weight; RB, number of rotatable bonds; TPSA, total polar surface area.

**FIGURE 11:**
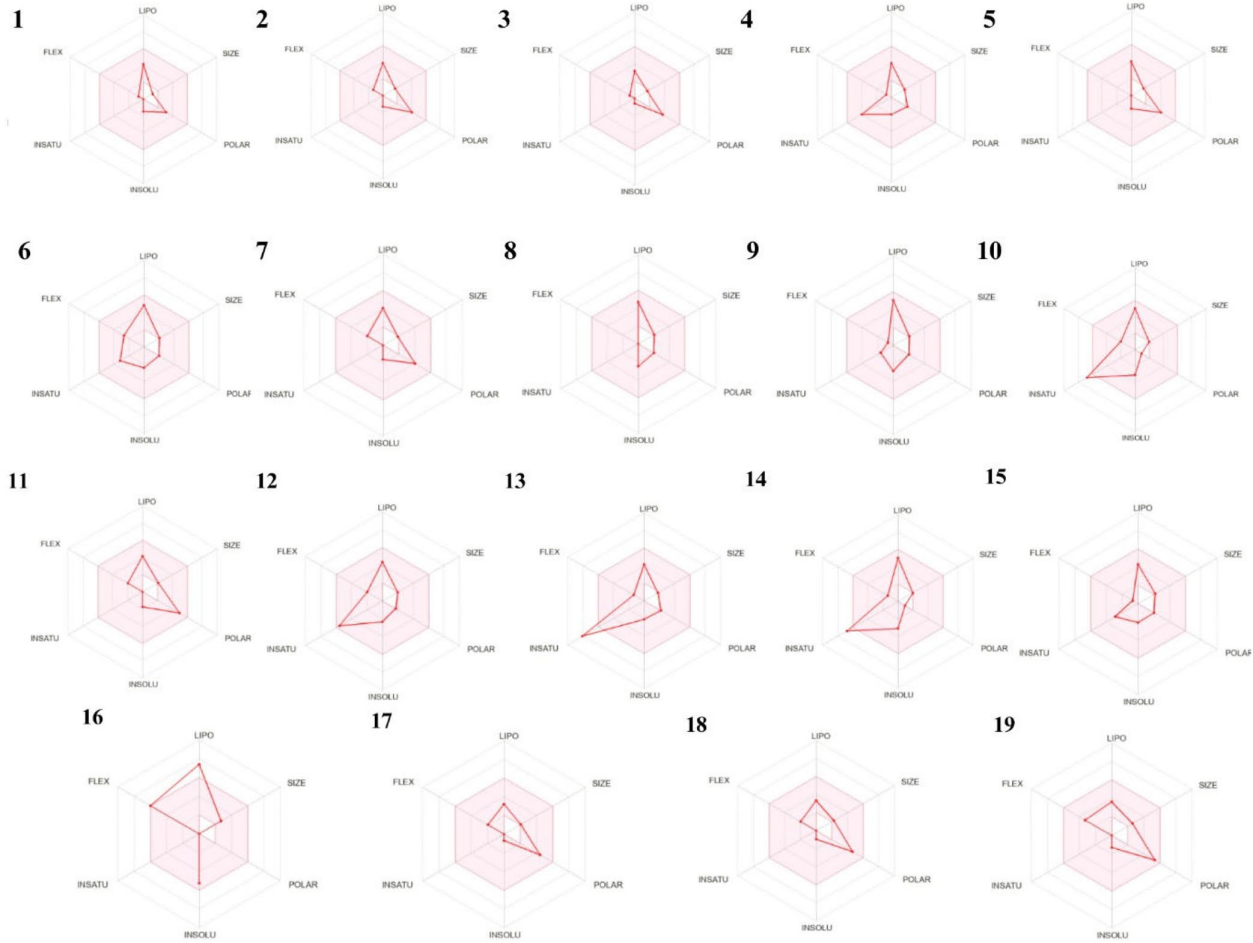
Physicochemical space for oral bioavailability of all compounds constituting the essential oil of *F. clypeata* .

Figure 11 is a study showing the physicochemical properties of compounds suitable for oral bioavailability. The presence of compounds in a very high ratio in the colored area means that they also comply with the physicochemical parameters. When the image is examined, it is determined that all our compounds obtained, especially compound 9 and compound 15, are more prevalent in the colored area.

## 4 Discussion

Essential oils consist of two or three main components, present in very elevated concentrations of 20%–70%, with other components in trace amounts (Chouhan et al. 2017). They are chemically derived from terpenoids, esters of aromatic and aliphatic acids, and phenolic compounds, and vary between plant parts and plant species. Essential oils are hydrophobic, break down with lipids found in the cell membrane of mitochondria and bacteria, and disrupt cell configurations, making them more permeable. As a result, critical molecules and ions largely leak out of the bacterial cell, leading to cell death (Chouhan et al. 2017). The primary function of the essential oils in the plant is to protect the plant by acting as antimicrobials, antivirals, and insecticidal agents.

In this study, according to the GS‐MS analysis results of *F. clypeata* essential oil, the most dominant compounds among 20 different compounds that make up a total of 99.76% of the plant essential oils are dimethyl disulfide with 73.13% and dimethyl trisulfide with 19.87%. Dimethyl disulfide and dimethyl trisulfide are alk(en)yl sulfides that occur as essential oil components of species belonging to the garlic ( *Allium sativum* ) and onion ( *Allium cepa* ) (Li et al. 2022; Kim, Huh, et al. 2004; Kim, Kim, et al. 2004). In a study, it was reported that the antimicrobial activity of the *Allium* genus is due to the sulfur compounds obtained from the amino acid S‐allyl‐L‐cysteine sulfoxide in its content with the assistance of the cysteine sulfoxide lyase enzyme (Cavallito and Bailey 1944; Stoll and Seebeck 1951). Alliin (S‐allyl‐Lcysteine sulfoxide), which is the main S‐alkenyl‐L‐cysteine sulfoxide in garlic, is broken down into allicin (allyl 2‐propenethiosulfinate), and this compound has been stated to be the antimicrobial agent of garlic (Small et al. 1947). Another study stated that *E. coli* treated with allyl methyl disulfide and dimethyl trisulfide damaged the bacterial cell surface and membranes and was reported to cause changes in β‐galactosidases and lead to cellular nucleic leakage (Wu et al. 2023). It has been demonstrated that allyl methyl disulfide and dimethyl trisulfide damage bacterial cell nucleic acids and proteins and reduce the adenosine triphosphatase activities of the cells (Wu et al. 2023). Sulfides were found in garlic oil as diallyl tetrasulfide, diallyl trisulfide, diallyl pentasulfide, and diallyl hexasulfide, which have more sulfur atoms. As the number of sulfur atoms they contain increases, compounds show more potent antimicrobial activity than those with fewer sulfur atoms (Ross et al. 2001). The inhibitory effect of dimethyl trisulfide against four yeast strains was studied, and its MIC value was found to be 20 ppm. However, this value was determined to be 200 ppm against various bacterial strains (Kyung and Fleming 1997). Some sulfur compounds from plants, including dimethyl trisulfide, inhibit the growth of yeasts more than the growth of bacteria (Kyung and Fleming 1997). This study we conducted also supports the result here. In this study, considering the MIC values, the antifungal activity of *F. clypeata essential oil* is significantly more pronounced than its antibacterial activity. While the antifungal MIC value of *F. clypeata essential oil* is between 0.039 and 0.312 μL/mL, its antibacterial MIC value goes up to 3.750 μL/mL. However, it should be emphasized that *F. clypeata essential oil* generally has a strong inhibitory effect against all tested microorganism types.

The number of sulfur atoms present determines the strength of antimicrobial activity (Kyung and Fleming 1997; Kim, Huh, et al. 2004; Kim, Kim, et al. 2004; O'Gara et al. 2000). The formation of trisulfide by adding a single sulfur atom to disulfides significantly increases the antimicrobial activity. The strength of the antimicrobial activity of garlic oil is expected to be determined by different sulfur concentrations. Various garlic oil samples showed wide variations in sulfur contents (Kim, Huh, et al. 2004; Kim, Kim, et al. 2004; O'Gara et al. 2000; Lawson et al. 1991). Sulfides with more than six sulfur atoms should have more potent antimicrobial activity at minimal concentrations than those with fewer sulfur atoms. In a comparative analysis of dimethyl disulfide and trimethyl disulfide, they had more muscular activities against *Candida utilis* than *S. aureus* (Kim, Huh, et al. 2004; Kim, Kim, et al. 2004). Several studies have shown how sulfites, including dimethyl disulfide, affect cellular activities. One group suggested that dimethyl disulfide inactivates active papain by forming an inactive papain‐methyl sulfide complex (Steven et al. 1981). Another group reported that dimethyl disulfide can damage red blood cells by reacting with the SH groups of red blood cell membranes (Smith 1980). Therefore, sulfites are expected to react via the same mechanism as microbial cells. Sulfides are also predicted to react with the sulfhydryl groups of proteins to inhibit the growth of microorganisms. It has been reported that allicin (or thiosulfinates) and sulfite, found in high amounts in onion and garlic oil, inhibit the growth of yeast cells (Connor and Beuchat 1984). The fact that the sulfur compounds dimethyl disulfide and dimethyl trisulfide dominate the majority of the essential oil in this study explains its antimicrobial activity and effectiveness on fungal species, which is more significant than its effectiveness on bacterial cells.

The third and fourth most abundant compounds in the plant essential oil we obtained are 2,3,5‐trithiahexane 5‐oxide and 2,4,5‐trithiahexane 2,2‐dioxide compounds with 2.75% and 1.60% ratios, respectively. 2,3,5‐trithiahexane 5‐oxide, a compound found extensively in onions and garlic, has been mentioned for its biological benefits (Mbadiko et al. 2023; Singh et al. 2001; Block 1985, 1990). Although these two compounds are found in many plant extracts, there is no study yet that has contributed to the literature regarding their antimicrobial properties and this mechanism (Mbadiko et al. 2023; Xi et al. 2023; Kouokam et al. 2002; Lim et al. 1999). However, in this study, although these compounds were found in small amounts in the essential oil content, they are among the dominant compounds. Thus, they are thought to contribute to our experimental results. The fifth most abundant compound in plant essential oil is the 2‐methyl‐3‐phenylpropanal compound, which is in the 0.69% ratio. In the antimicrobial activity study conducted by Vukovic et al. with the essential oil obtained from *Ballota nigra* , the 2‐methyl‐3‐phenylpropanal compound was detected as one of the dominant compounds with a rate of 4.32%, and the antimicrobial activity was attributed to this compound (Vukovic et al. 2009). Although there are few studies in the literature on the antimicrobial properties of the 2‐methyl‐3‐phenylpropanal, the health benefits of this compound have been mentioned (Ben‐Khalifa et al. 2018; Garner et al. 2007).

The selective inhibition of essential oil towards the BChE enzyme is valuable for the anticholinesterase activity due to the different roles of designated enzymes. Although AChE and BChE enzymes are responsible for the hydrolysis of the neurotransmitter acetylcholine, the main difference in the roles of these enzymes emerges in which stage of disease they would be active. Both enzymes are present in the brain, but AChE predominates (80%) in healthy brains, and BChE also takes responsibility for the hydrolysis of the acetylcholine process. However, BChE is considered to have a minor function in ACh levels. The level of AChE remains unchanged or decreases with the progression of AD, and BChE gains importance due to the dramatic increase. This explains the critical role of BChE in the late stages of AD. This fact makes researchers think selective BChE inhibitors may benefit AD treatment (Khudina et al. 2024; Tobuse et al. 2024; Lane et al. 2006). The related outcome for the anticholinesterase potency of essential oil was found to be valuable due to the selective inhibition towards the BChE enzyme, and the oil could be considered a potential target for the anticholinesterase study.

Free radicals, cationic radicals, and non‐radicals, namely hydrogen peroxide, superoxide, and nitric oxide, are classified as reactive oxygen species (ROS) and are responsible for oxidative stress in living systems (Schmidt et al. 2012). Oxidative stress can be defined as the disruption of the balance between antioxidants and oxidants in favor of the oxidant side (Jenner 2003; Lyras et al. 1997). They have an essential role in various diseases: diabetes, cancer, cardiovascular, and auto‐immune diseases (Jenner 2003; Lyras et al. 1997; Süzen 2007; Ratnam et al. 2006). The oxidation process could be blocked or slowed down by molecules called antioxidant substances (Jenner 2003). Due to the abovementioned circumstances, the proven free radical antioxidant (DPPH) ability of essential oil is valuable evidence for detecting the antioxidant potential of the essential oil of *F. clypeata* . The obtained value represents the promising potency of the oil and is found to be comparable with the antioxidant potentials of different extracts of *F. clypeata* (Zengin et al. 2020). Although the cationic radical scavenging inhibition value is found to be lower compared to the standards due to the higher IC_50_ concentration, the CUPRAC assay also detected valuable targets for antioxidant studies. The obtained value was found to be better IC_50_ concentrations compared to the standards.

To the best of our knowledge, no specific studies have exclusively focused on the minor constituents identified in the essential oil analyzed in our work. Nevertheless, several of these compounds have been reported to play important roles in inhibiting anticholinesterase enzymes and exhibiting antioxidant activities. Existing literature highlights that naturally occurring compounds such as linalool, α‐terpineol, and endo‐borneol contribute significantly to these bioactivities. A few relevant studies are summarized below to support this observation.

For instance, Ryan et al. demonstrated that linalool may act as a potential inhibitor of butyrylcholinesterase (BChE), although it showed no significant inhibition against acetylcholinesterase (AChE) (Ryan and Bryan 1988). In a separate study, it was reported that linalool, found in the essential oil of *Thymus vulgaris* L., contributed to relatively low AChE inhibition activity compared to other components (Jukic et al. 2006). Similarly, α‐terpineol, which constitutes a substantial portion (5.93%) of the essential oil derived from *Xylopia aethiopica* seeds, has been associated with both anticholinesterase activity (IC_50_ for AChE inhibition = 1.21 ± 0.06 mg/mL) and antioxidant capacity (IC_50_ for DPPH radical scavenging = 2.19 ± 0.09 mg/mL), likely due to its synergistic interaction with other compounds in the oil (Sulaimon et al. 2020). The known antioxidant and neuroprotective effects of α‐terpineol further support its role in combating oxidative stress and cholinesterase‐related dysfunction, with implications for managing neurodegenerative disorders such as Alzheimer's disease (Trinh et al. 2003; Savelev et al. 2003).

Additionally, a recent study by Lal et al. investigated the pharmacological properties of anethole‐rich essential oil (ARCHEO) extracted from *Clausena heptaphylla*. The findings revealed that ARCHEO displayed greater AChE inhibitory activity than galantamine, a standard therapeutic agent. This pronounced activity was attributed not only to the high content of cis‐anethole but also to the potential synergistic interactions with minor compounds present in the oil. Moreover, the antioxidant effects of anethole were linked to its ability to scavenge free radicals, inhibit lipid peroxidation, chelate metal ions, and enhance the activity of antioxidant enzymes (Lal et al. 2022).

These findings collectively underline the functional significance of both major and minor constituents in essential oils, particularly in contributing to their anticholinesterase and antioxidant activities.

Molecular docking results (docking scores) showed that especially Endo‐Borneol (**8**), α‐Terpineol (**9**), 1‐(2,6,6‐Trimethyl‐1,3‐cyclohexadien‐1‐yl)ethanol (**15**), and 2,6,11‐Trimethyldodecane (**16**) were more prone to antibacterial activity, and it was thought that the size difference (big‐small) between these compounds might have caused this (Table 4). For this reason, a table showing the interaction residues and bond type was prepared only for these 4 compounds with high dock scores. It was observed that compound **8** formed hydrogen bonds with VAL 199, compound **9** with ASN 80, compound **15** with SER 82, and compound **16** did not form hydrogen bonds (Table 5).

## 5 Conclusion

This study reveals the antimicrobial, antioxidant, and enzymatic inhibition potentials towards the anticholinesterase and anti‐diabetes enzymes of *F. clypeata* essential oil for the first time. The GS‐MS analysis determined the plant essential oil content, and dimethyl disulfide and dimethyl trisulfide were found to dominate most of the plant essential oil. This study determined that *F. clypeata* essential oil had a strong antimicrobial activity attributed to dimethyl disulfide and dimethyl trisulfide, and fungal cells were more sensitive to plant essential oil than bacteria. Scientific proof from this study supports further examination of *F. clypeata* essential oil to create and design a novel pharmacotherapeutic agent for the treatment of diseases caused by pathogenic or opportunistic infections. The enzyme inhibition profile was found to be favorable for the anticholinesterase enzymes. AChE and BChE were both inhibited by the essential oil of *F. clypeata* with reasonable IC_50_ values; more importantly, the BChE value was found to be better than the standard Galanthamine and to present a selective inhibition for anticholinesterase study. In the case of α‐glucosidase enzyme inhibition, the essential was found to be inactive and determined as not a suitable target for anti‐diabetic studies. Previously, promising α‐glycosidase inhibitory activity of a methanol extract of the plant was reported. However, current results indicate low potential against the same enzyme for the essential oil of the plant. Possible hypoglycemic effects of the plant might be due to non‐volatile compounds such as various flavonoids reported from the methanol extract of the plant (Zengin et al. 2020). The antioxidant potentials were also promising for the essential oil of *F. clypeata* . Specifically, free radical scavenging activity and Cupric Ion‐Reducing capacity were highly potent and more effective than the compared standards. Molecular docking study showed that dock scores and interacting residues, especially Endo‐Borneol (**8**), α‐Terpineol (**9**), 1‐(2,6,6‐Trimethyl‐1,3‐cyclohexadien‐1‐yl)ethanol (**15**), and 2,6,11‐Trimethyldodecane (**16**), were more prone to antibacterial activity, and the physicochemical parameters of these compounds were found to be suitable. Although the molecular dock scores were better with 1EA1, which was selected for the antifungal activity study, it was determined that no interaction, such as hydrogen bonding pi interaction was observed when the relevant images were examined.

## Author Contributions

**Tuba Unver:** conceptualization (lead), data curation (supporting), formal analysis (equal), investigation (equal), methodology (equal), project administration (lead), supervision (lead), validation (equal), visualization (equal), writing – original draft (lead), writing – review and editing (lead). **Murat Bingul:** data curation (equal), formal analysis (equal), investigation (equal), methodology (equal), project administration (supporting), supervision (supporting), validation (equal), writing – original draft (equal), writing – review and editing (equal). **Harun Uslu:** data curation (equal), formal analysis (equal), investigation (equal), methodology (equal), software (lead), validation (equal), visualization (equal), writing – original draft (supporting). **Ismet Gurhan:** data curation (equal), formal analysis (equal), investigation (equal), methodology (equal), validation (equal). **Bunyamin Goktas:** formal analysis (supporting), investigation (equal), methodology (equal), software (supporting), validation (supporting), visualization (supporting). **Hasan Sahin:** data curation (supporting), formal analysis (supporting), investigation (supporting), methodology (supporting), validation (supporting). **Mehmet Boga:** formal analysis (supporting), investigation (supporting), methodology (supporting), validation (supporting).

## Conflicts of Interest

The authors declare no conflicts of interest.

## Supporting information


Data S1.

## Data Availability

The data that support the findings of this study are available on request from the corresponding author.
