# Supplementary material for: Shedding Light on the Phytochemical and Biological Fingerprints of Fibigia clypeata (L.) Medik Essential Oil as a Pharmacotherapeutic Agent
Source: Food Sci Nutr. 2025 Jul 4;13(7):e70493. doi: 10.1002/fsn3.70493 (PMC12227661; doi:10.1002/fsn3.70493)
Supplement: Supplementary file 1 — Data S1. [file FSN3-13-e70493-s001.docx]

**Shedding Light on the Phytochemical and Biological Fingerprints of *Fibigia clypeata* (L.) Medik Essential Oil as a Pharmacotherapeutic Agent**

Biological Activity of *Fibigia clypeata*

Tuba Unver^1,*^, Murat Bingul^2^, Harun USLU^3^, Ismet Gurhan^4^, Bünyamin GÖKTAŞ^3, 5^

Hasan Sahin^6^, Mehmet Boga^7^

^1^Department of Pharmaceutical Microbiology, Faculty of Pharmacy, Inonu University, 44280, Malatya, Turkiye

^2^Department of Basic Pharmaceutical Sciences, Faculty of Pharmacy, Dicle University, Diyarbakır 21280, Turkiye

^3^Department of Pharmaceutical Chemistry, Faculty of Pharmacy, Fırat University, Elazığ, Turkey

^4^Department of Pharmaceutical Botany, Faculty of Pharmacy, Inonu University, 44280, Malatya, Turkiye

^5^Department of Pharmaceutical Chemistry, Graduate School, Anadolu University, Eskisehir, Turkiye

^6^Department of Pharmacognosy, Faculty of Pharmacy, Dicle University, Diyarbakır 21280, Turkiye

^7^Department of Analytical Chemistry, Faculty of Pharmacy, Dicle University, Diyarbakır 21280, Turkiye

* Correspondence to: Tuba UNVER

Inonu University,

Faculty of Pharmacy

Department of Pharmaceutical Microbiology

44280, Malatya, TURKEY

E: [tuba.unver@inonu.edu.tr](mailto:tuba.unver@inonu.edu.tr)

T: +90 507 071 8788

ORCIDs and E-mails:

Tuba UNVER: https://orcid.org/0000-0002-8655-2716, [tuba.unver@inonu.edu.tr](mailto:tuba.unver@inonu.edu.tr)

Murat BINGUL: <https://orcid.org/0000-0002-3909-0694>, [murat.bingul@dicle.edu.tr](mailto:murat.bingul@dicle.edu.tr)

Harun USLU: <https://orcid.org/0000-0001-8827-8557>, [huslu@firat.edu.tr](mailto:huslu@firat.edu.tr)

Ismet GURHAN: <https://orcid.org/0000-0001-7017-2447>, [ismet.gurhan@inonu.edu.tr](mailto:ismet.gurhan@inonu.edu.tr)

Bunyamin GOKTAS <https://orcid.org/0000-0003-2345-7313>, [bgoktas@firat.edu.tr](mailto:bgoktas@firat.edu.tr)

Hasan SAHIN: https://orcid.org/0000-0002-8325-8116, [eczsahin@gmail.com](mailto:eczsahin@gmail.com)

Mehmet BOGA: <https://orcid.org/0000-0003-4163-9962>, [mehmetboga1980@gmail.com](mailto:mehmetboga1980@gmail.com)

**
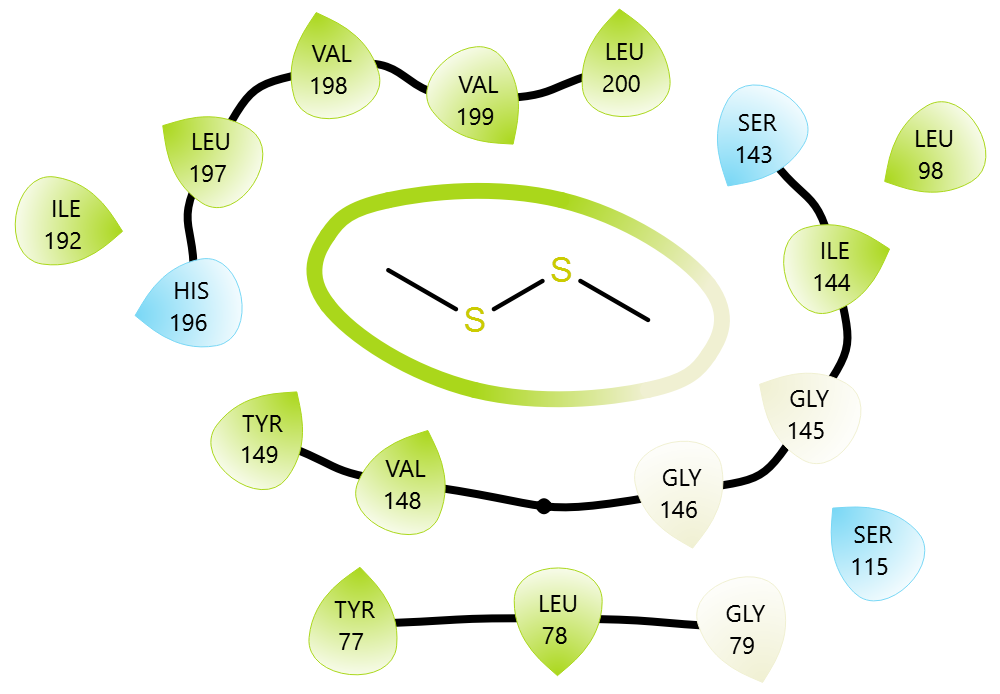
**

**Figure 1S.** 2D interaction diagram with 1HSK for **1**.


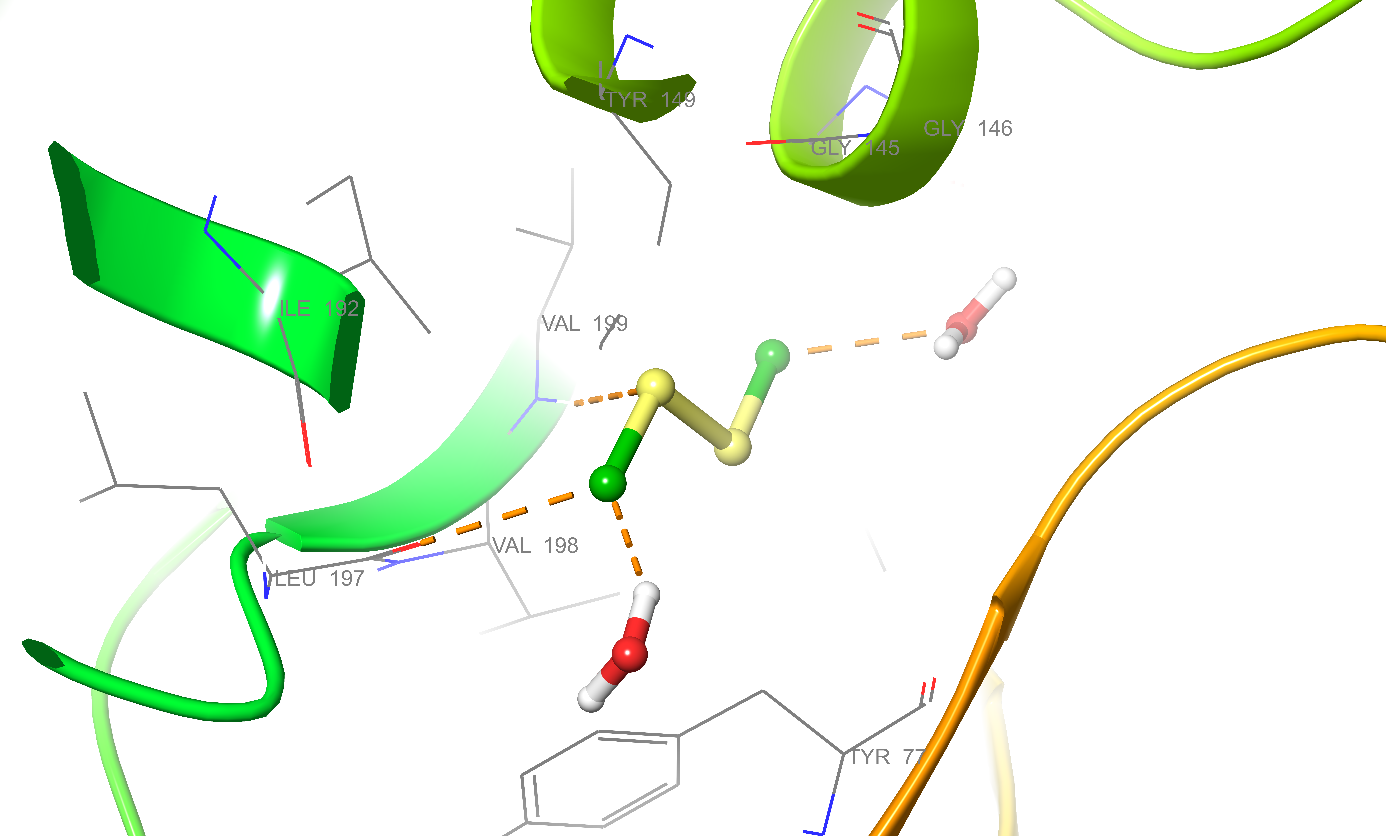


**Figure 2S.** 3D interaction diagram with 1HSK for **1**.

**
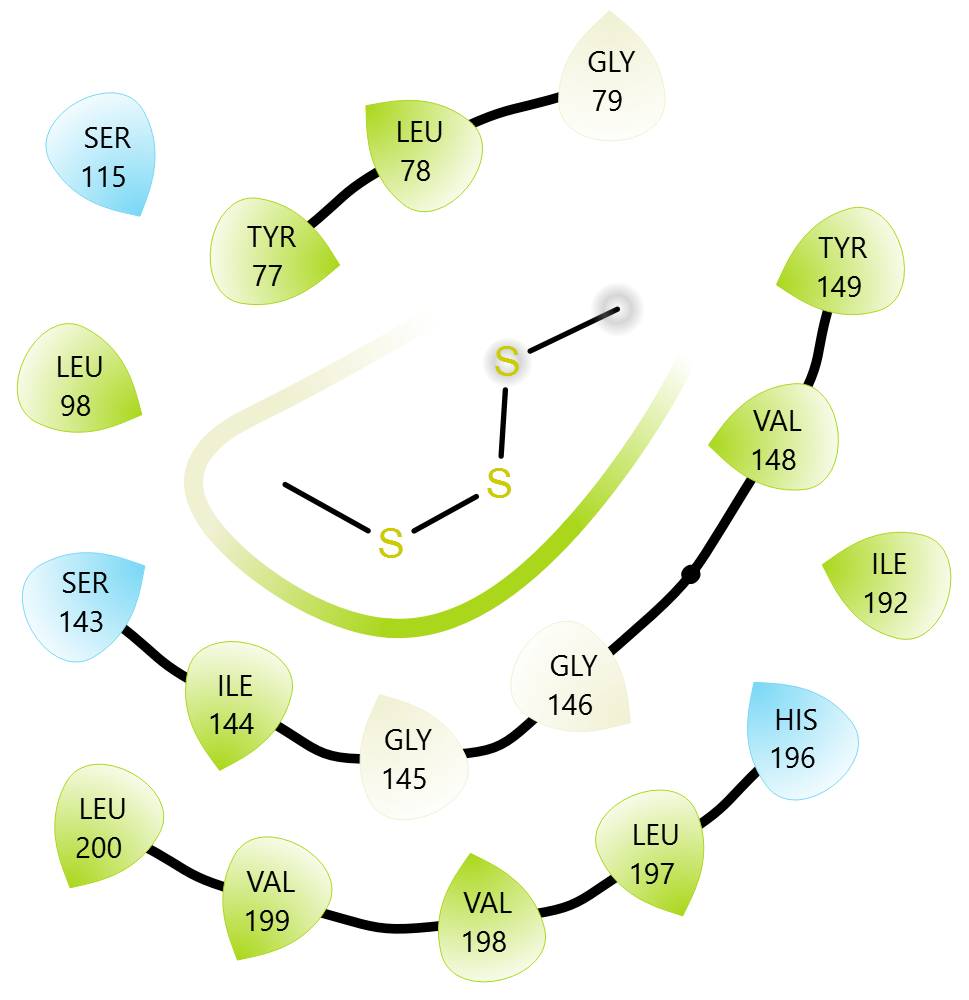
**

**Figure 3S.** 2D interaction diagram with 1HSK for **2**.


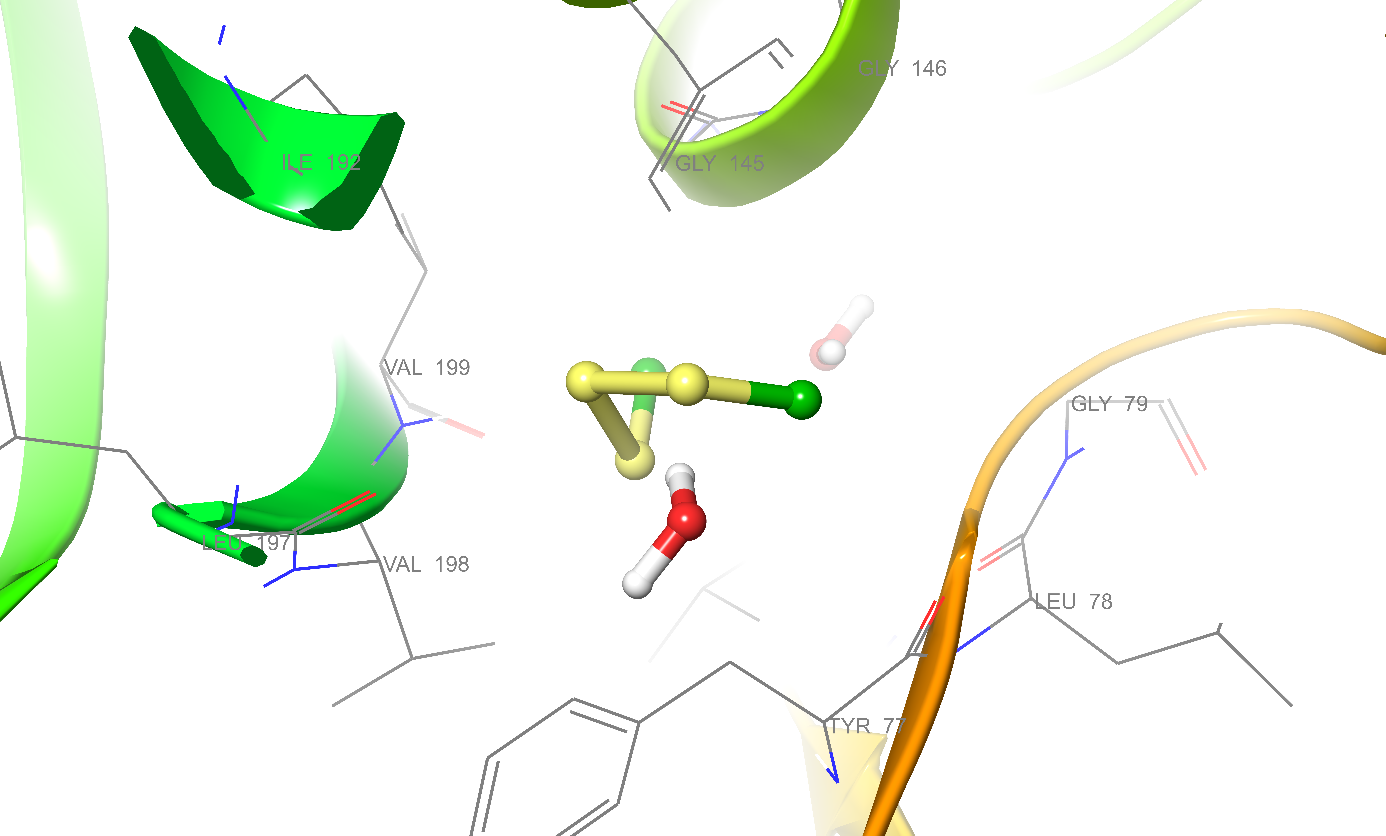


**Figure 4S.** 3D interaction diagram with 1HSK for **2**.

**
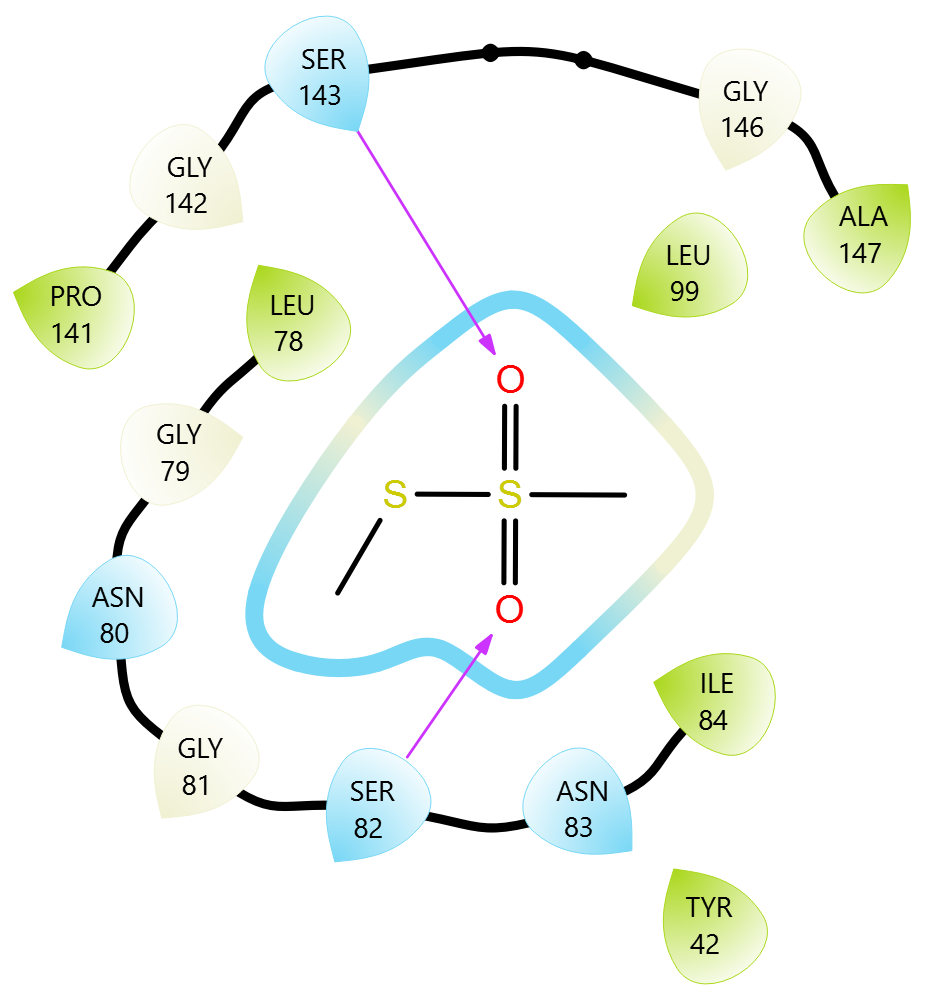
**

**Figure 5S.** 2D interaction diagram with 1HSK for **3**.


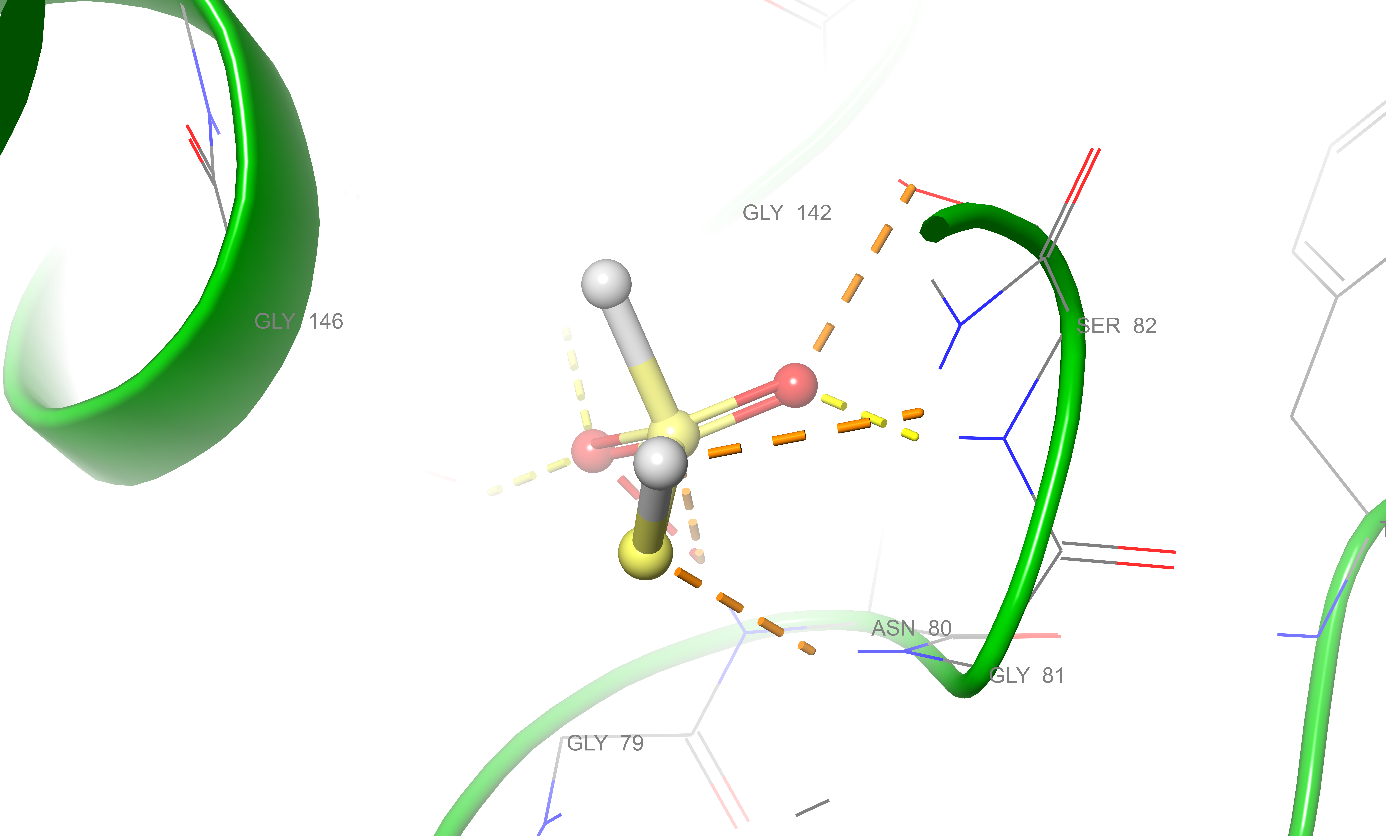


**Figure 6S.** 3D interaction diagram with 1HSK for **3**.

**
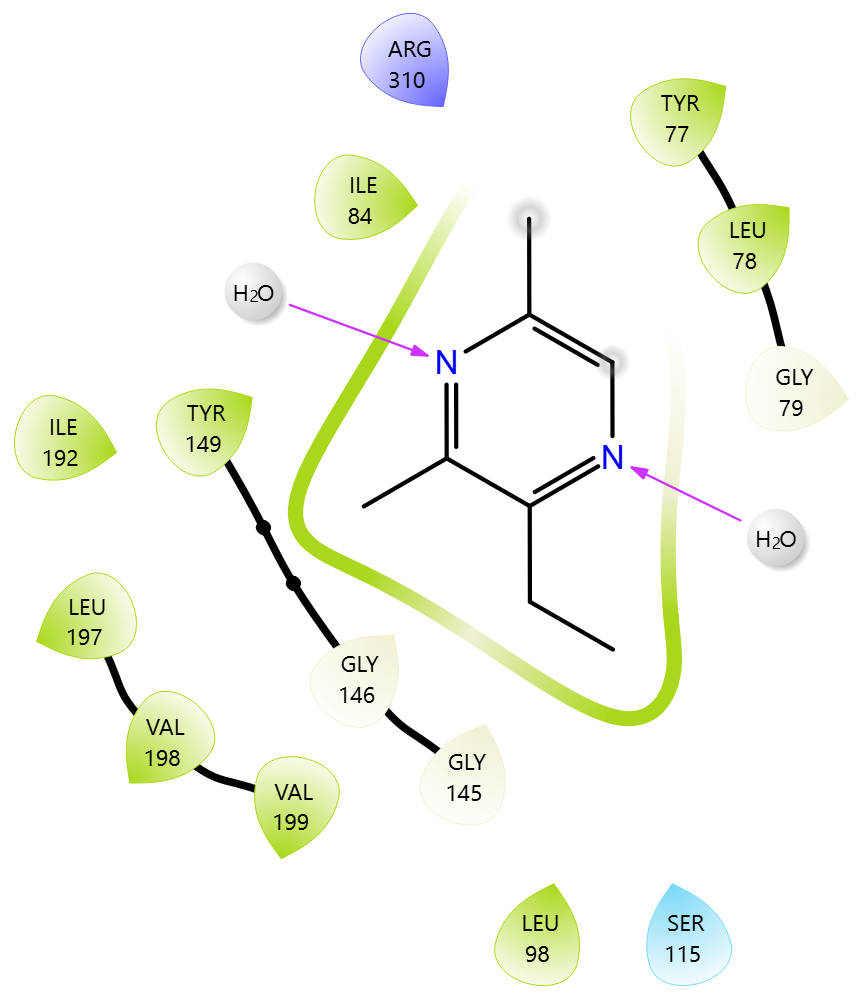
**

**Figure 7S.** 2D interaction diagram with 1HSK for **4**.


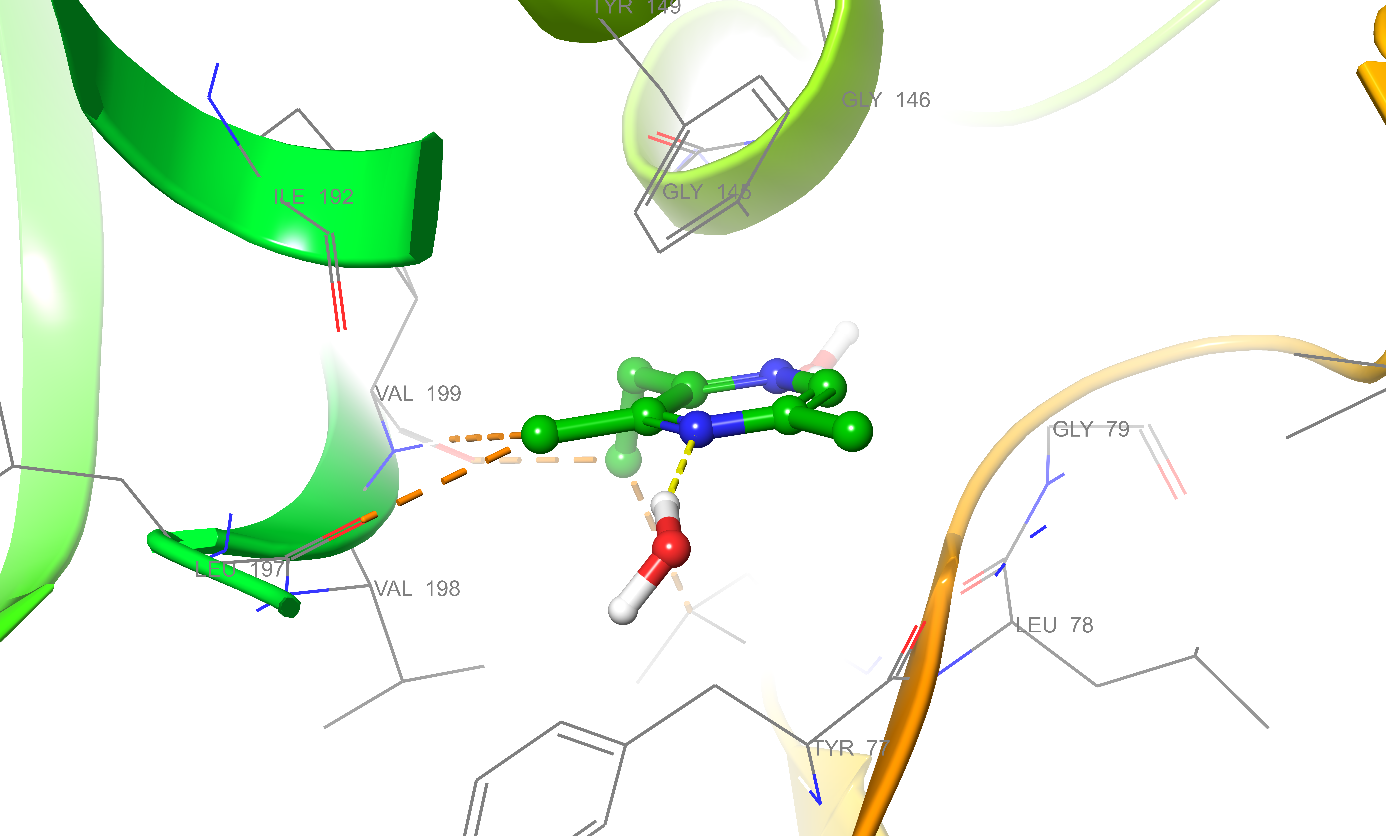


**Figure 8S.** 3D interaction diagram with 1HSK for **4**.

**
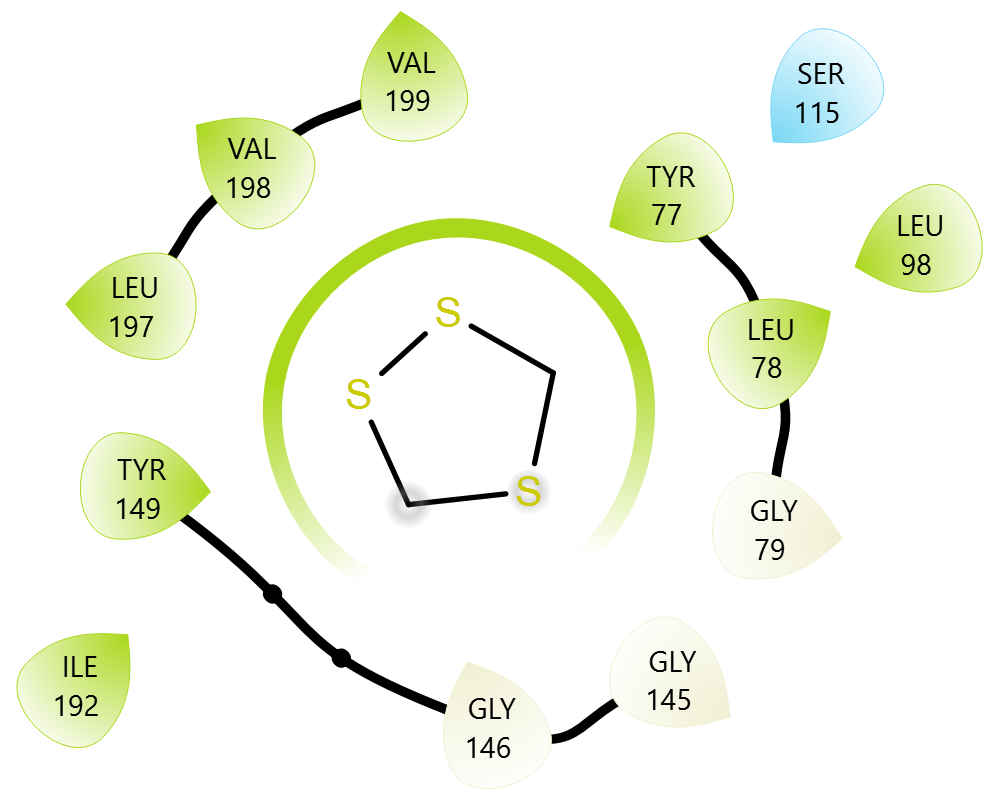
**

**Figure 9S.** 2D interaction diagram with 1HSK for **5**.


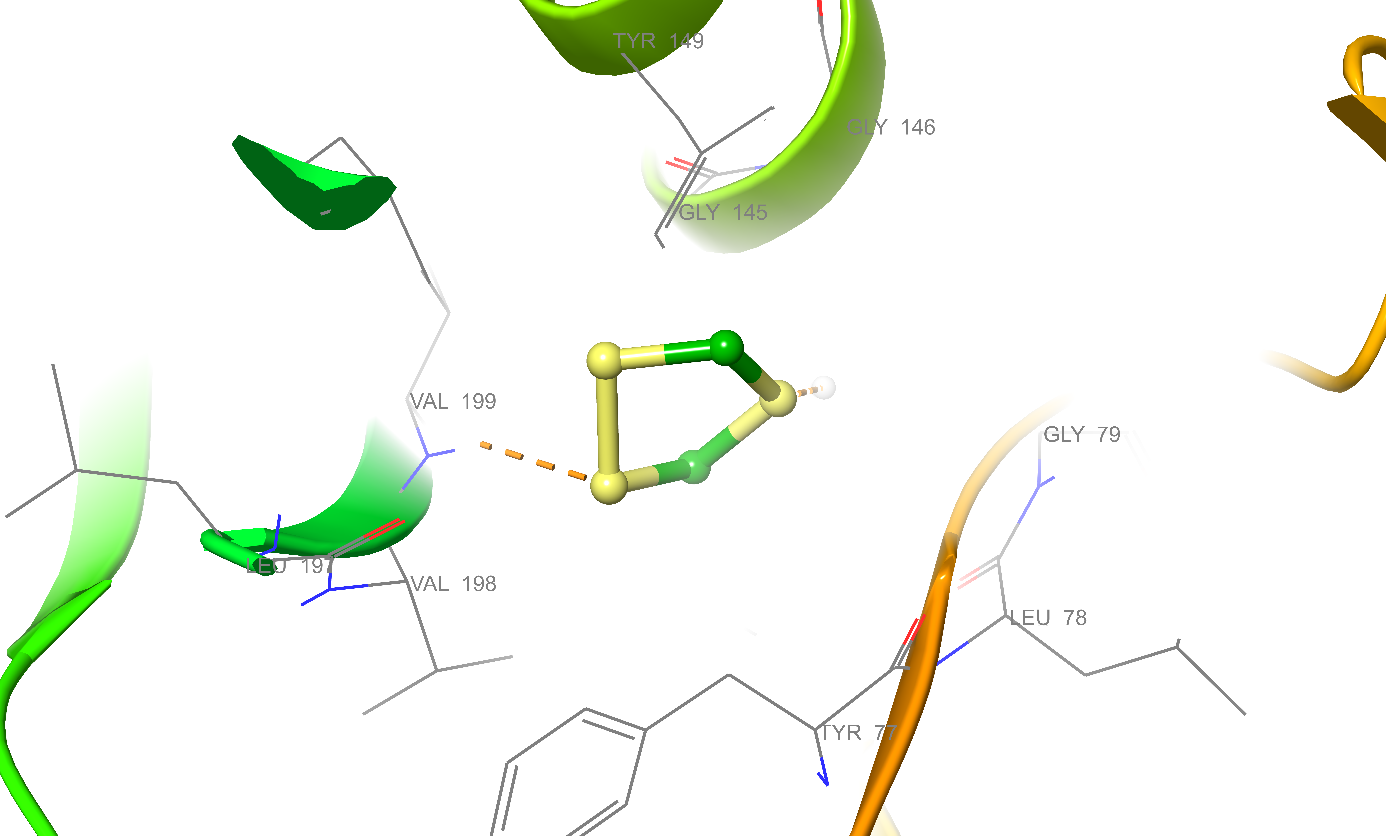


**Figure 10S.** 3D interaction diagram with 1HSK for **5**.

**
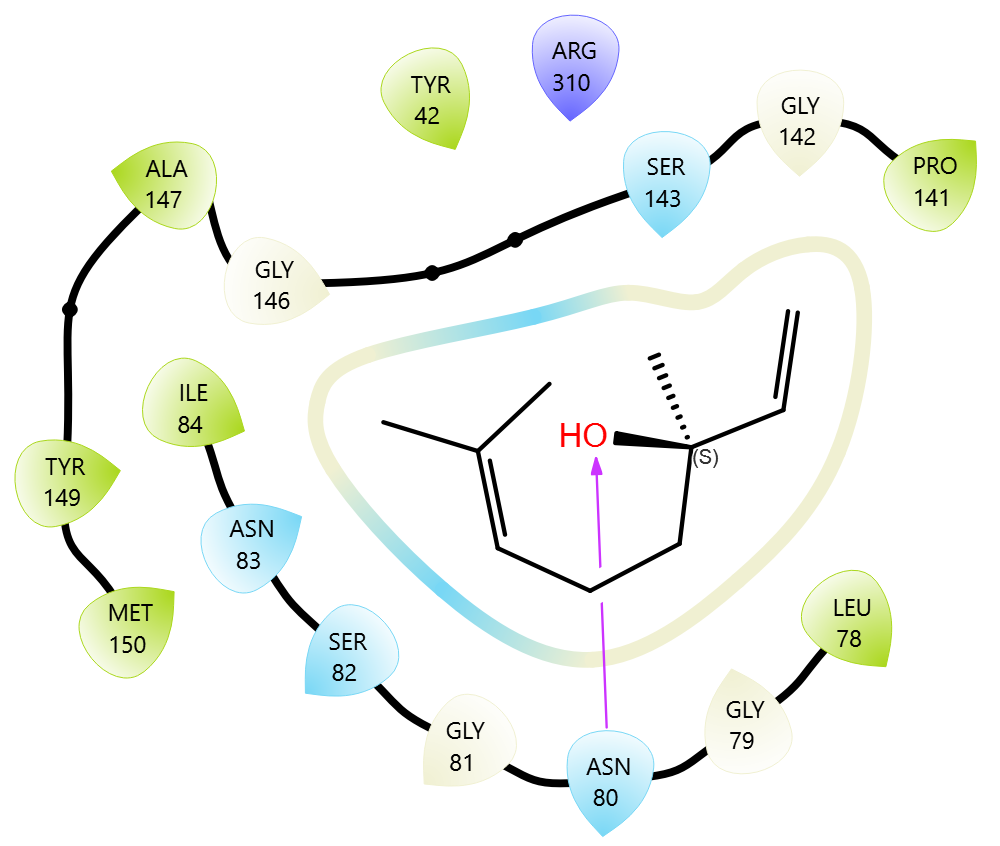
**

**Figure 11S.** 2D interaction diagram with 1HSK for **6**.


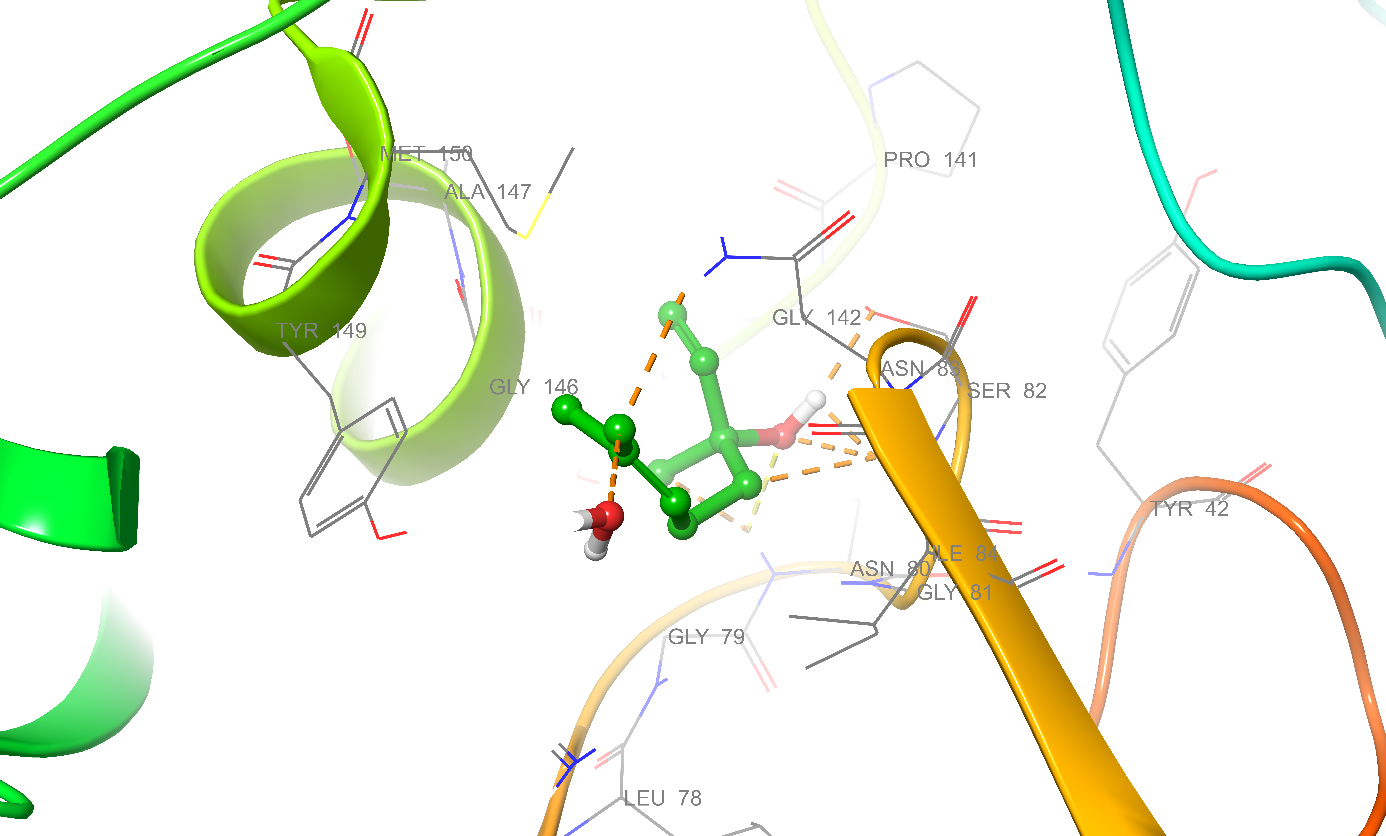


**Figure 12S.** 3D interaction diagram with 1HSK for **6**.


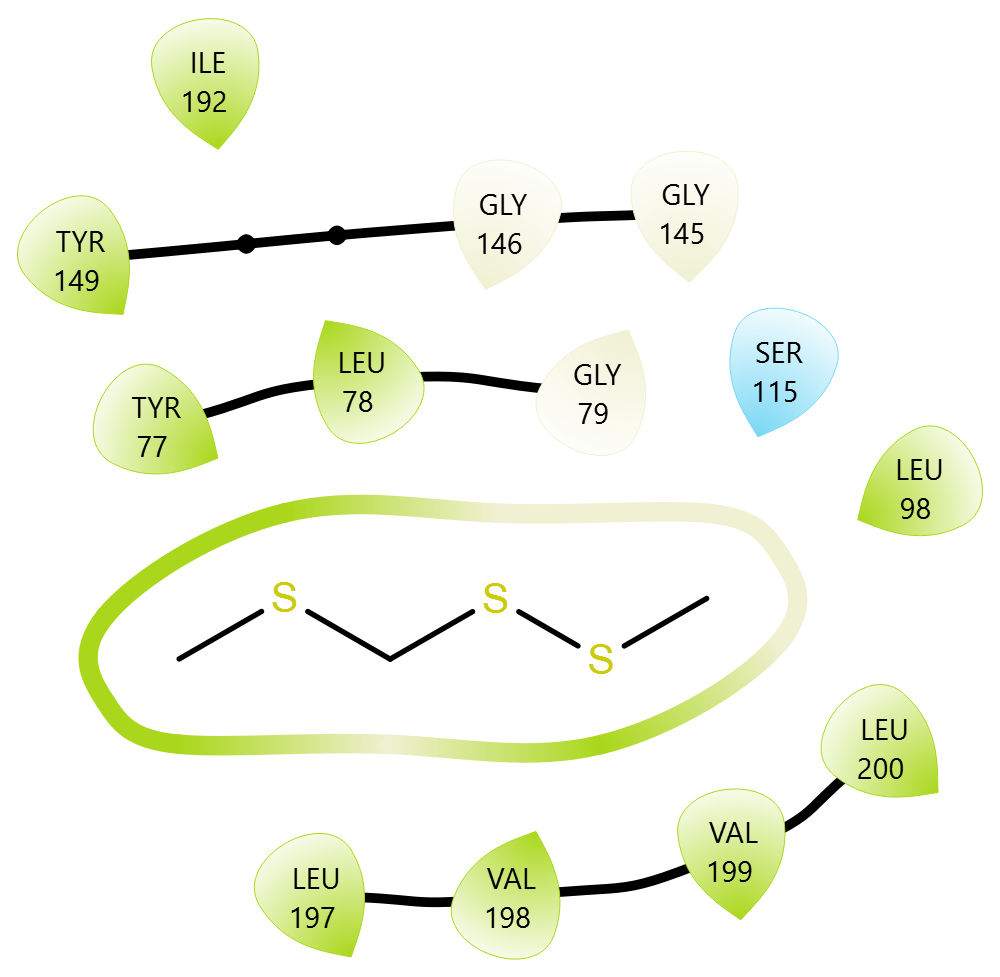


**Figure 13S.** 2D interaction diagram with 1HSK for **7**.


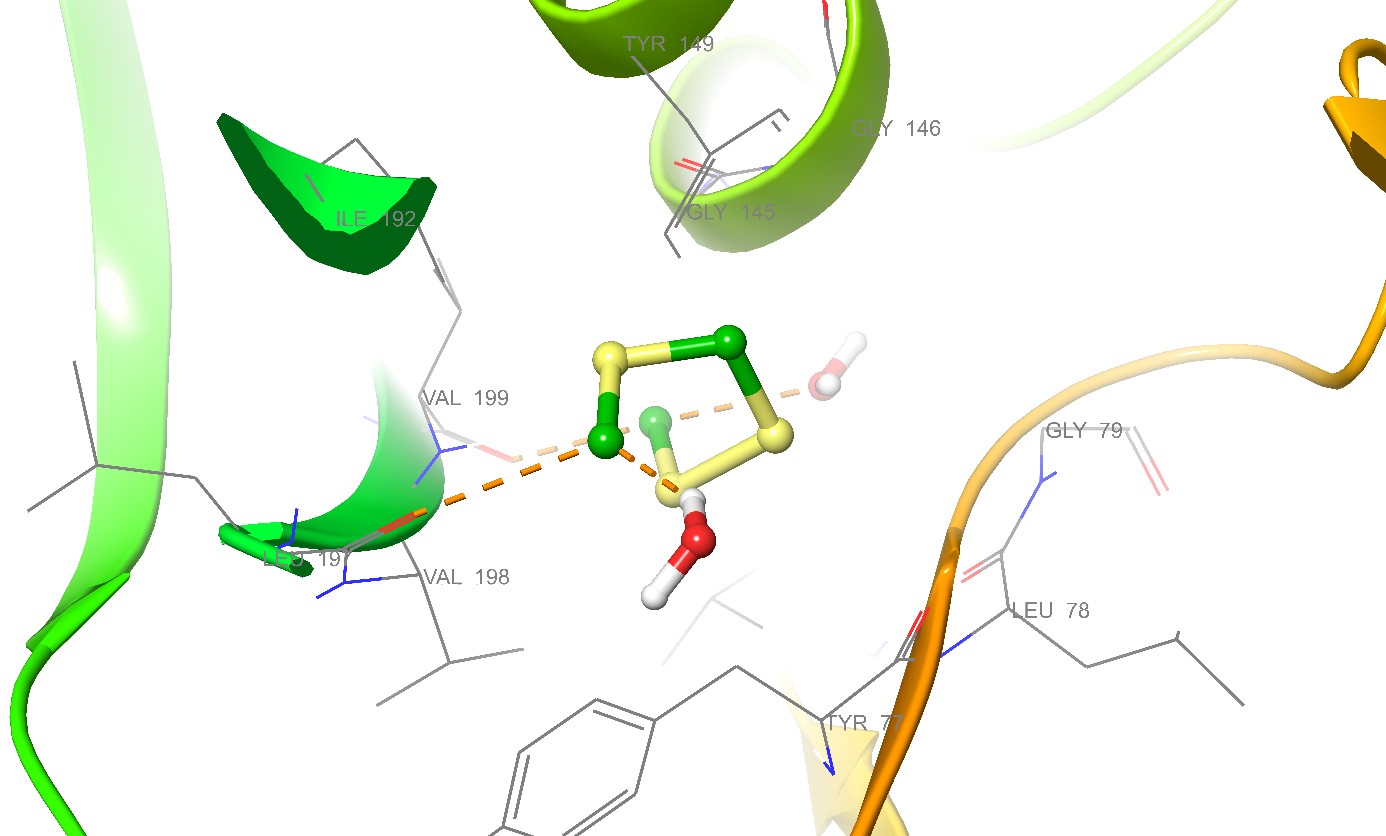


**Figure 14S.** 3D interaction diagram with 1HSK for **7**.

**
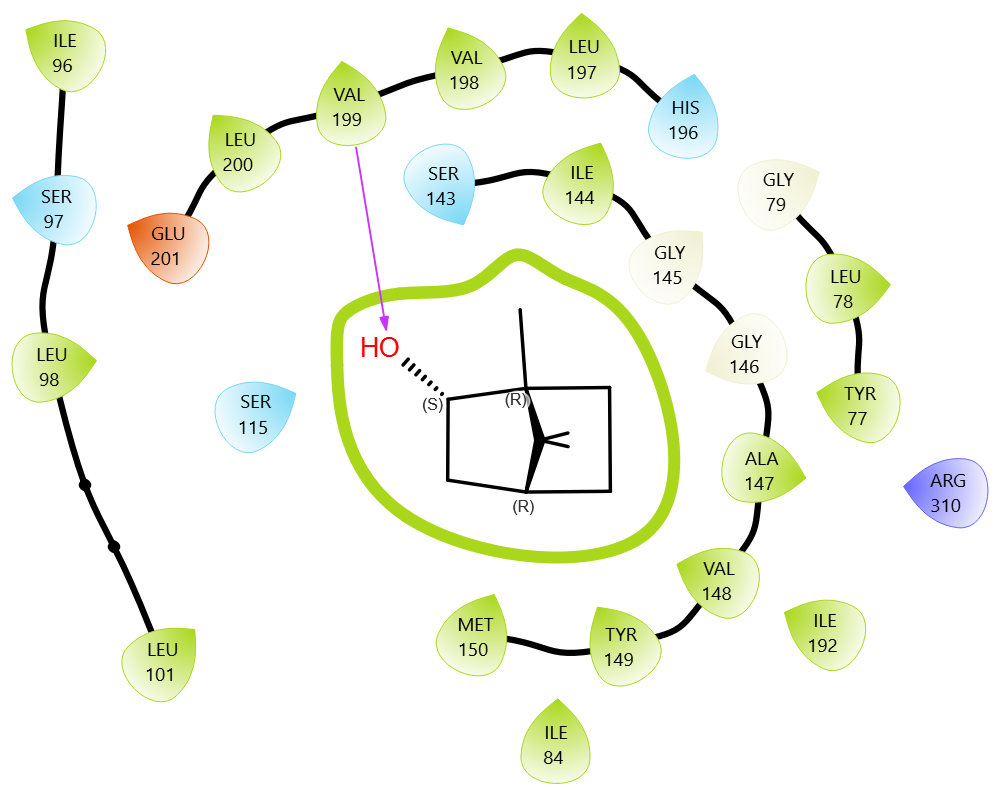
**

**Figure 15S.** 2D interaction diagram with 1HSK for **8**.


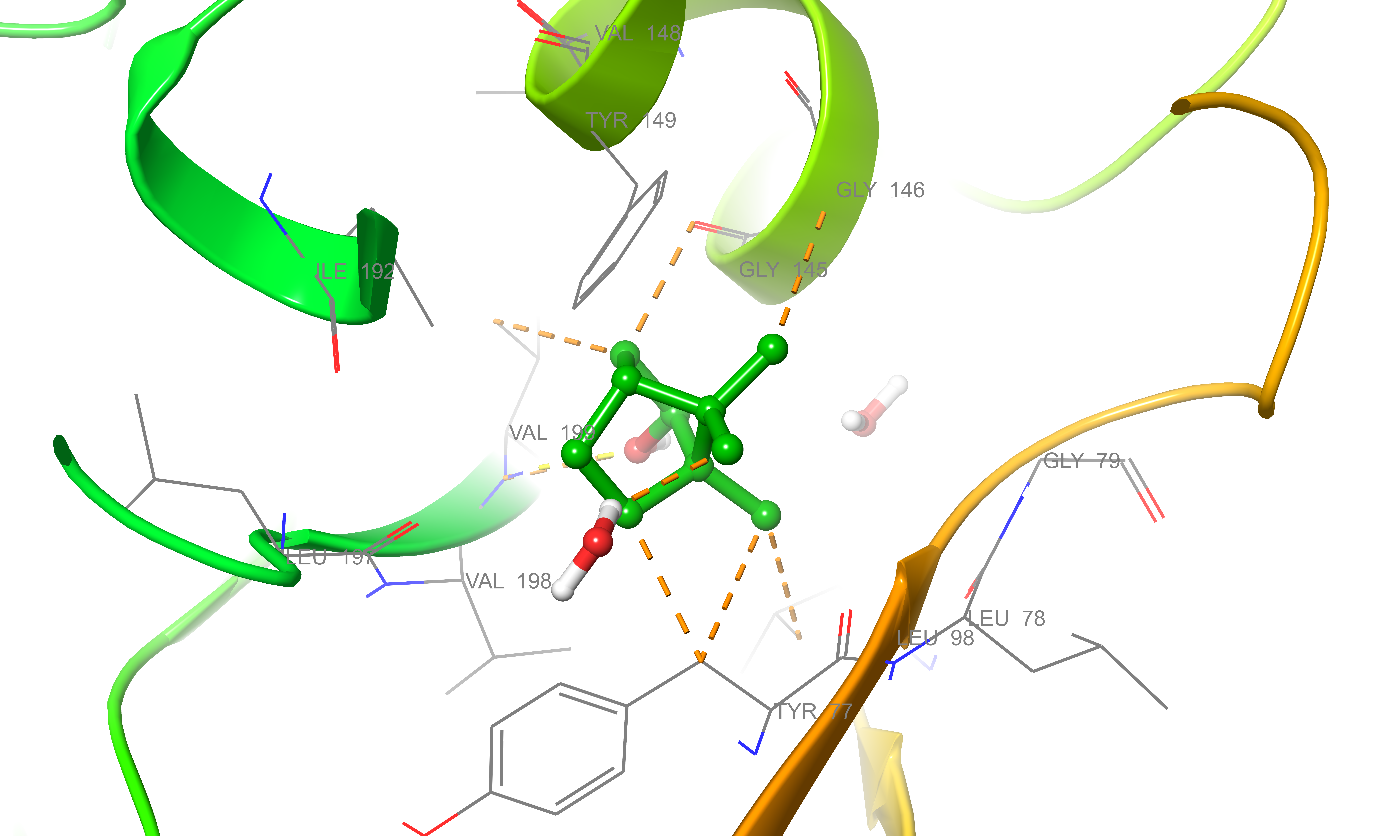


**Figure 16S.** 3D interaction diagram with 1HSK for **8**.

**
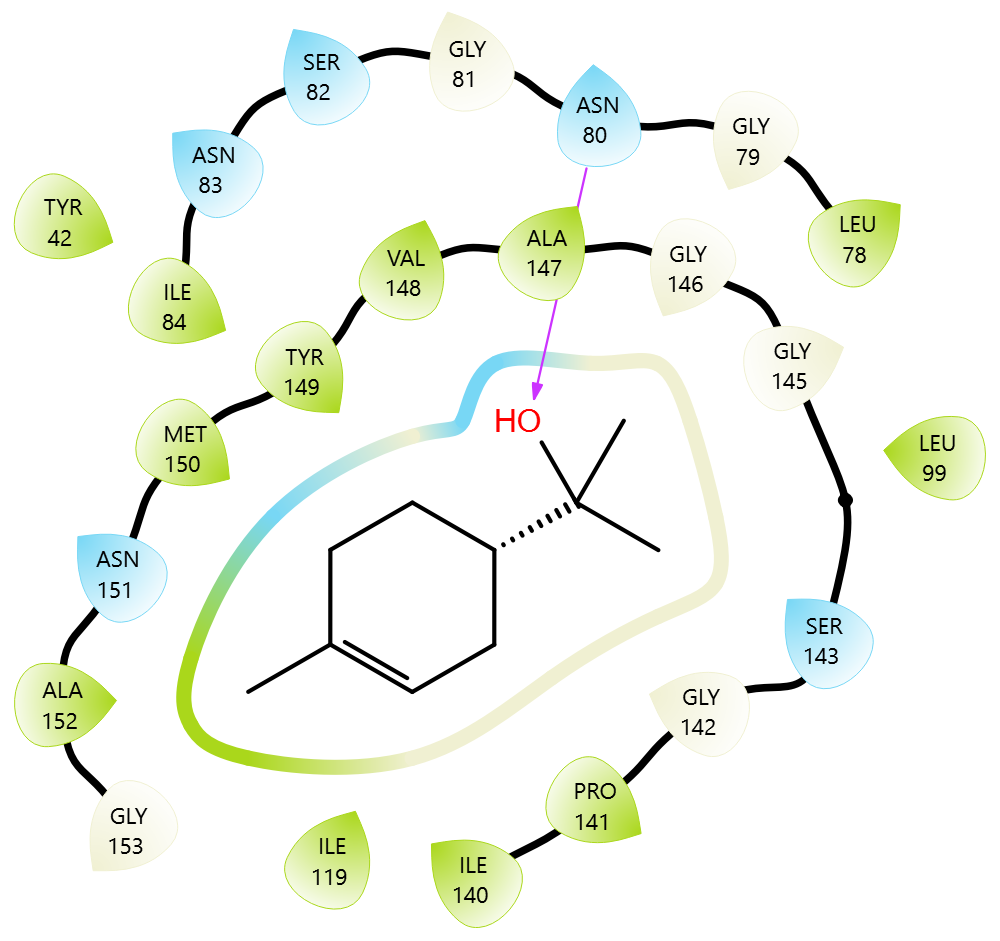
**

**Figure 17S.** 2D interaction diagram with 1HSK for **9**.


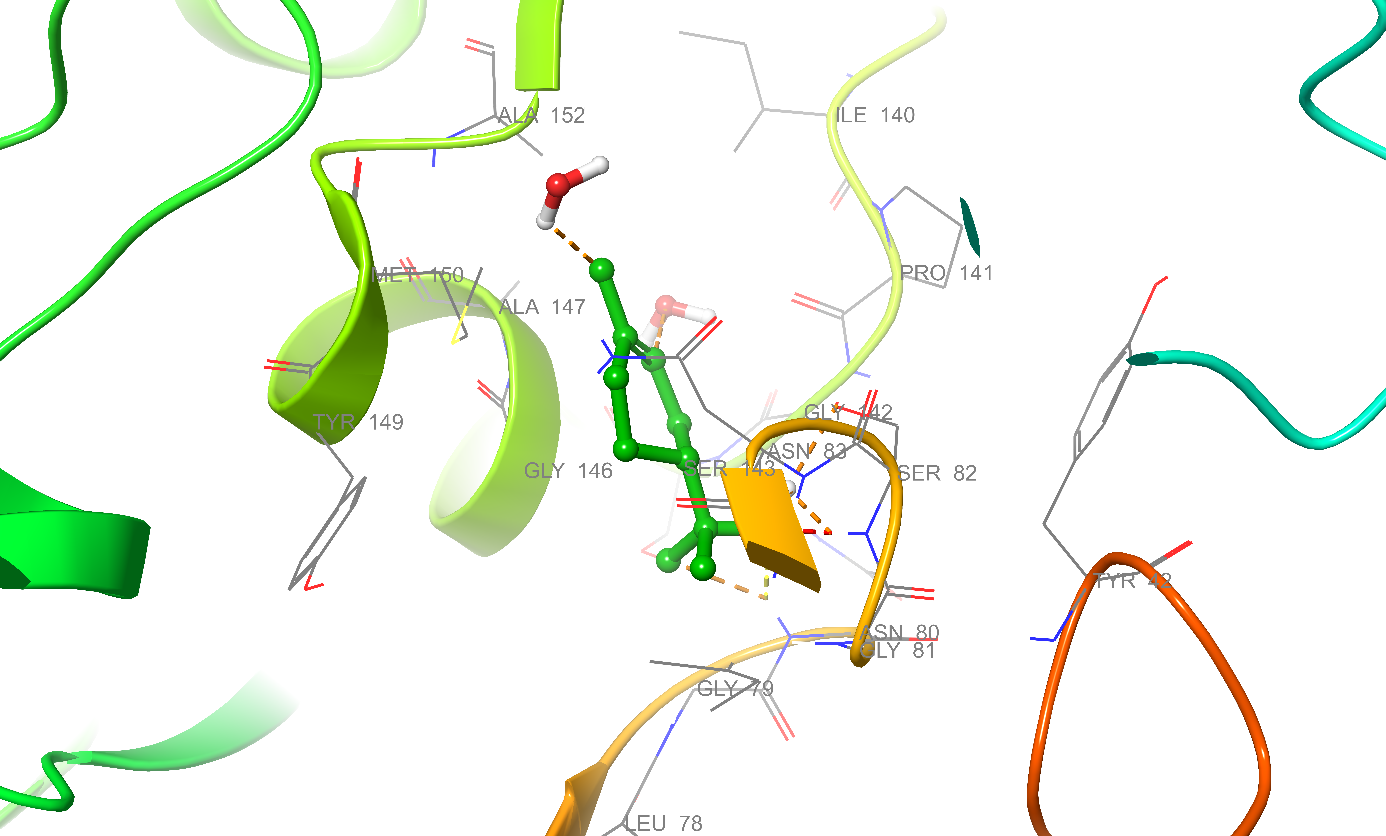


**Figure 18S.** 3D interaction diagram with 1HSK for **9**.

**
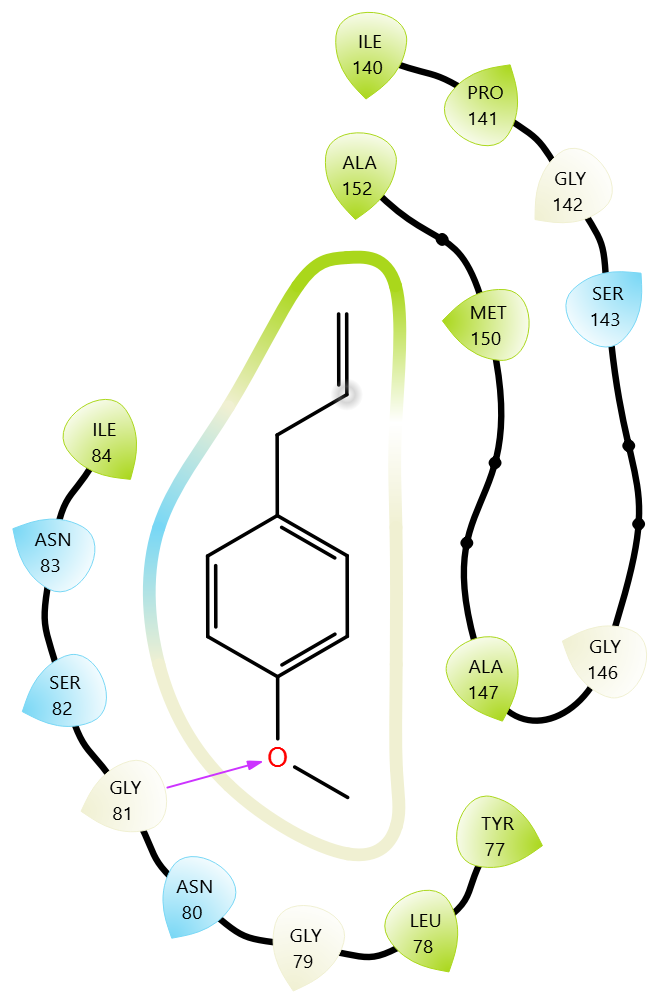
**

**Figure 19S.** 2D interaction diagram with 1HSK for **10**.


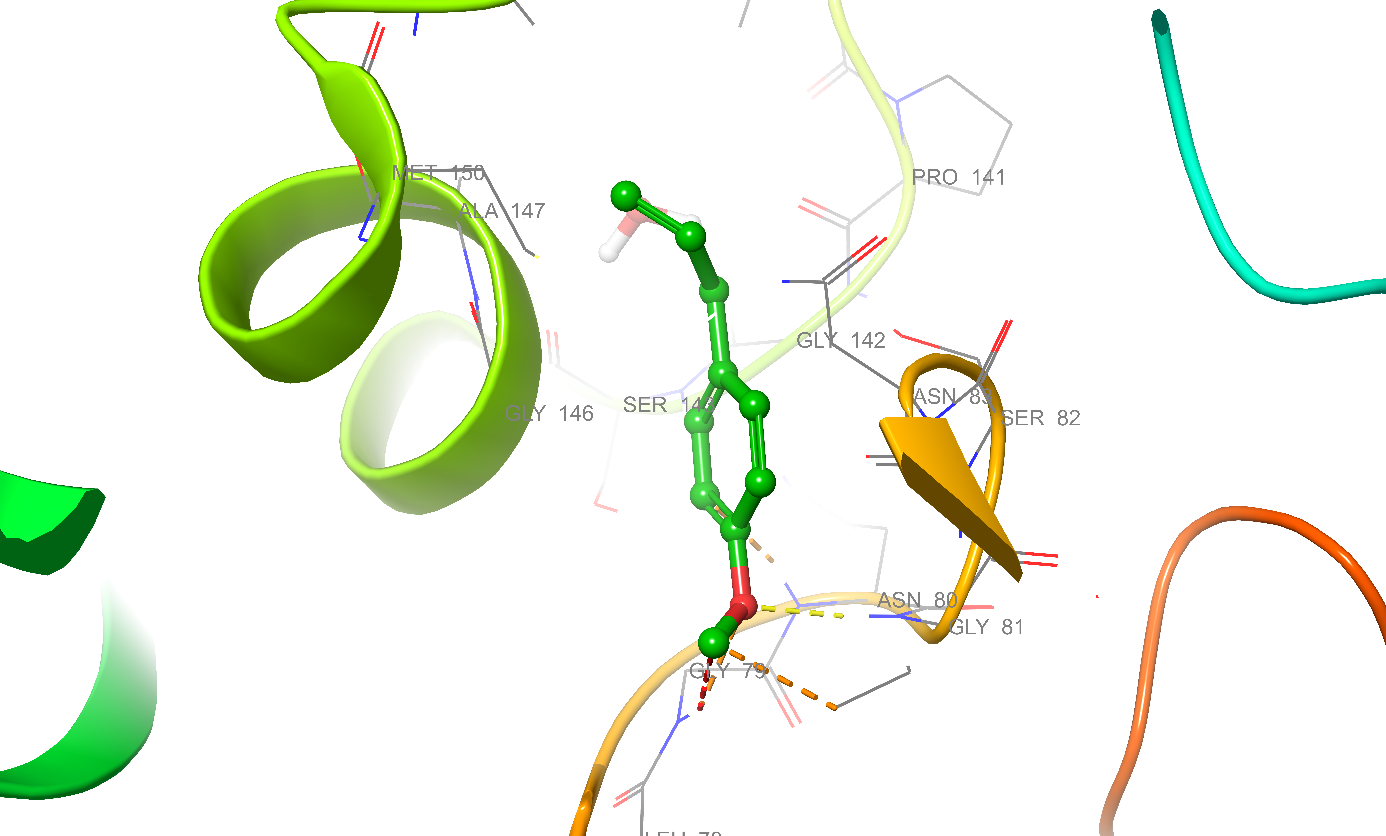


**Figure 20S.** 3D interaction diagram with 1HSK for **10**.

**
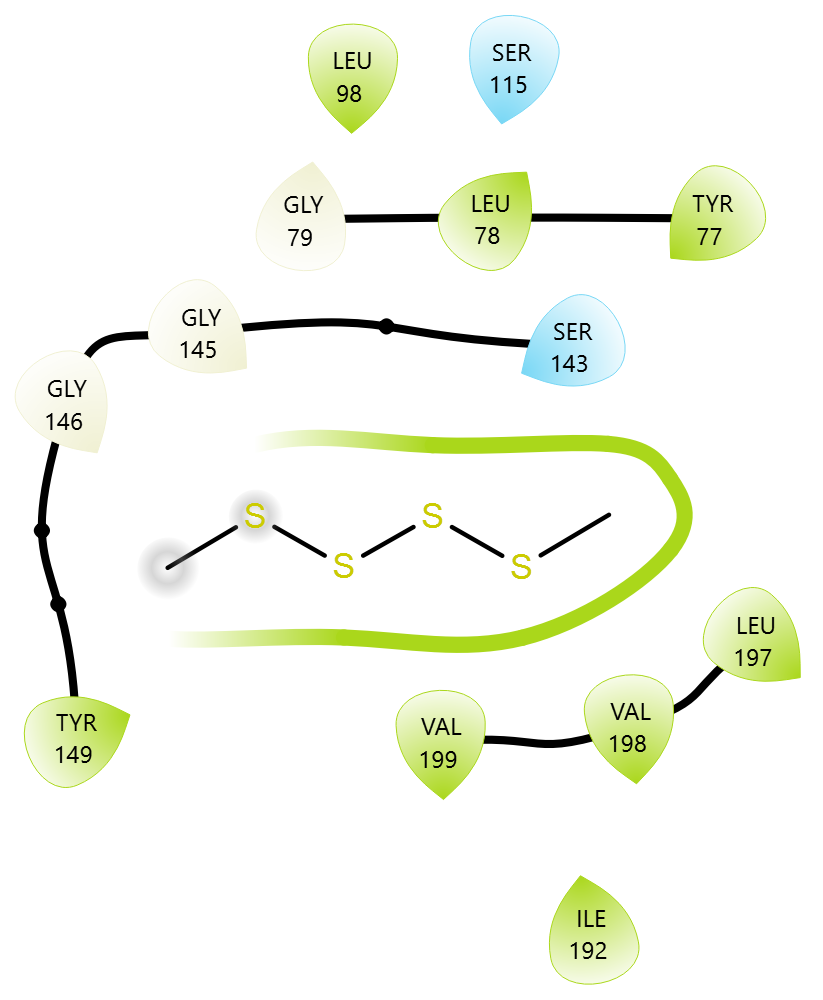
**

**Figure 21S.** 2D interaction diagram with 1HSK for **11**.


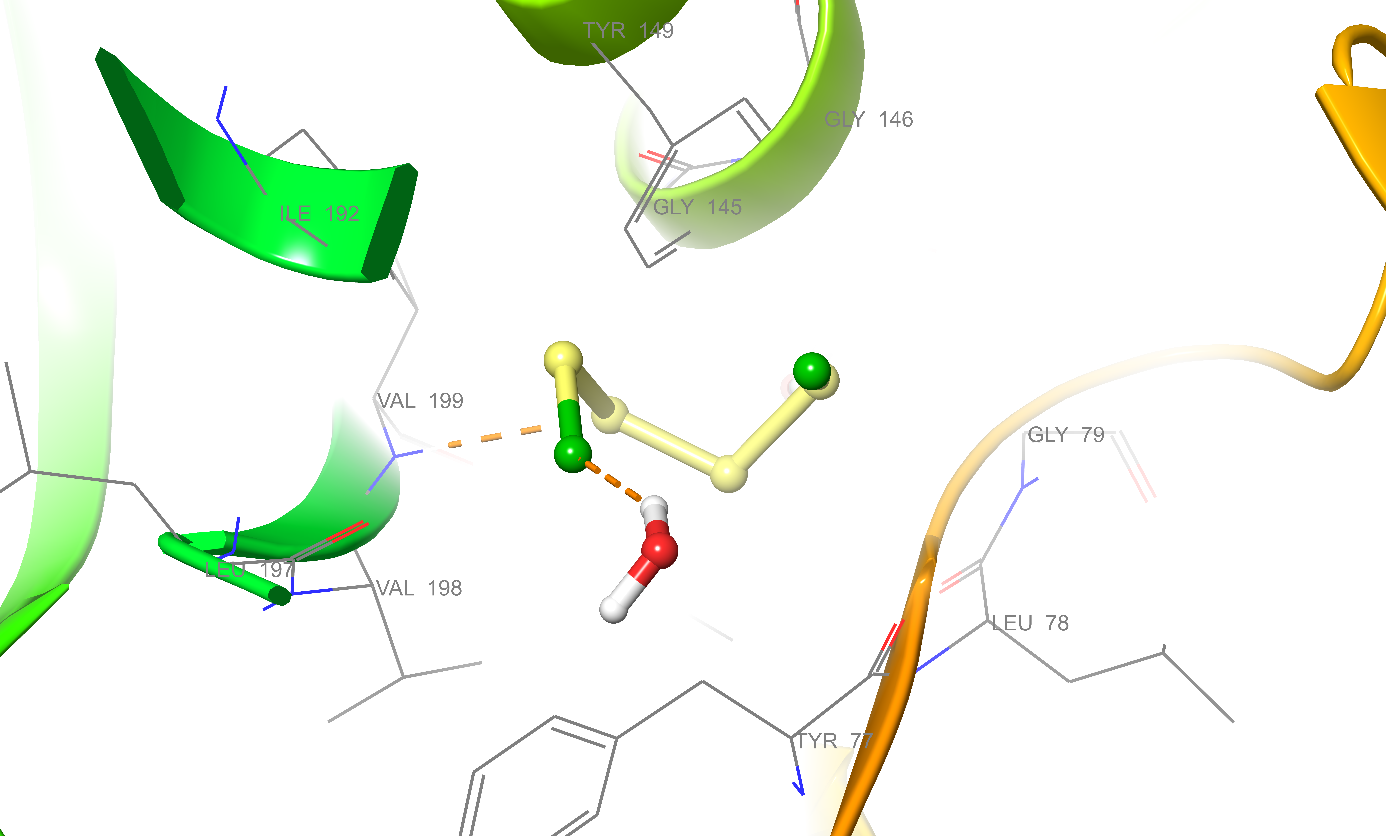


**Figure 22S.** 3D interaction diagram with 1HSK for **11**.

**
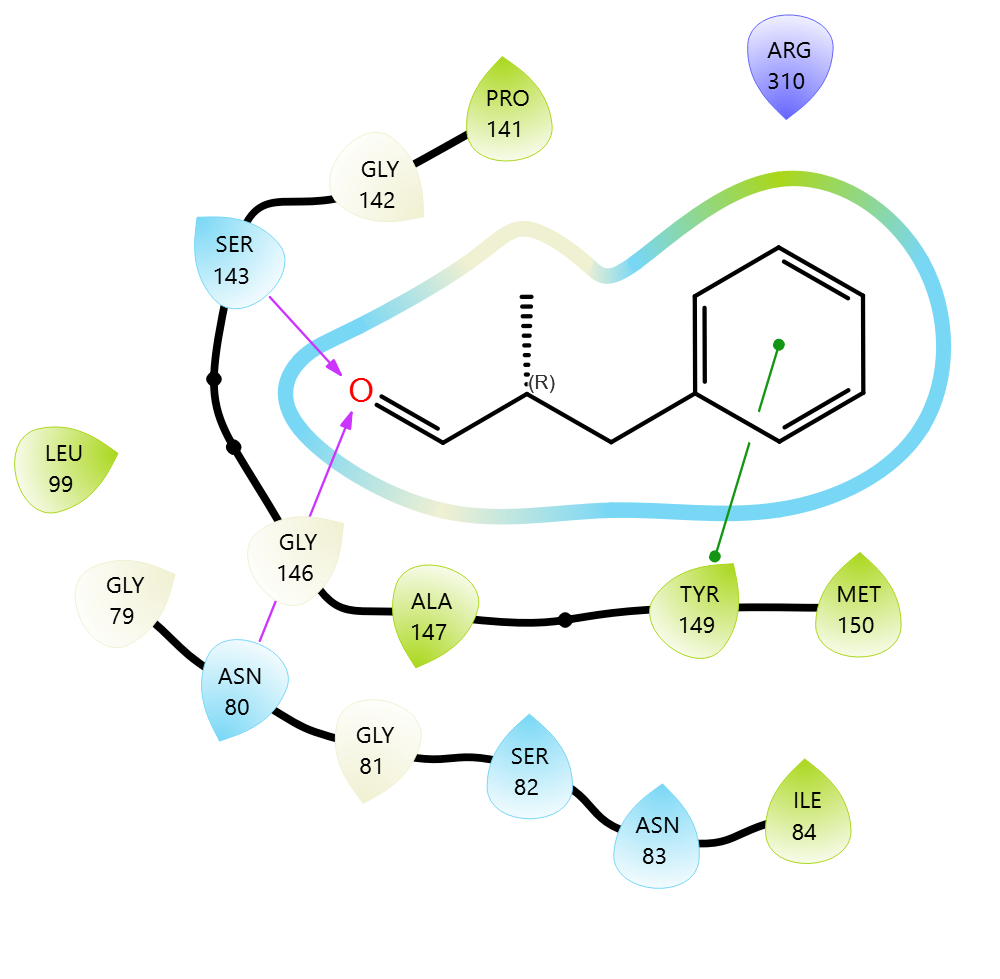
**

**Figure 23S.** 2D interaction diagram with 1HSK for **12**.


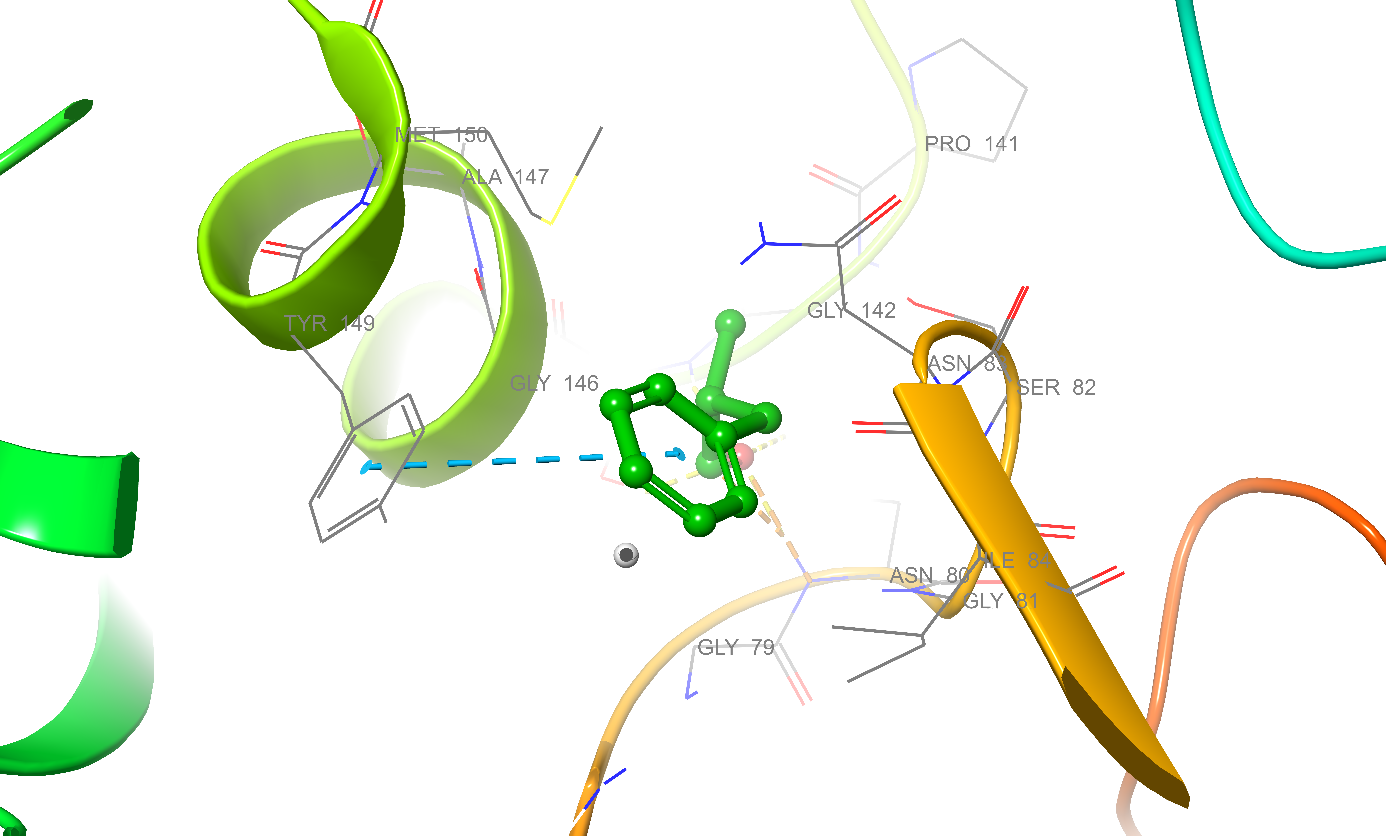


**Figure 24S.** 3D interaction diagram with 1HSK for **12**.

**
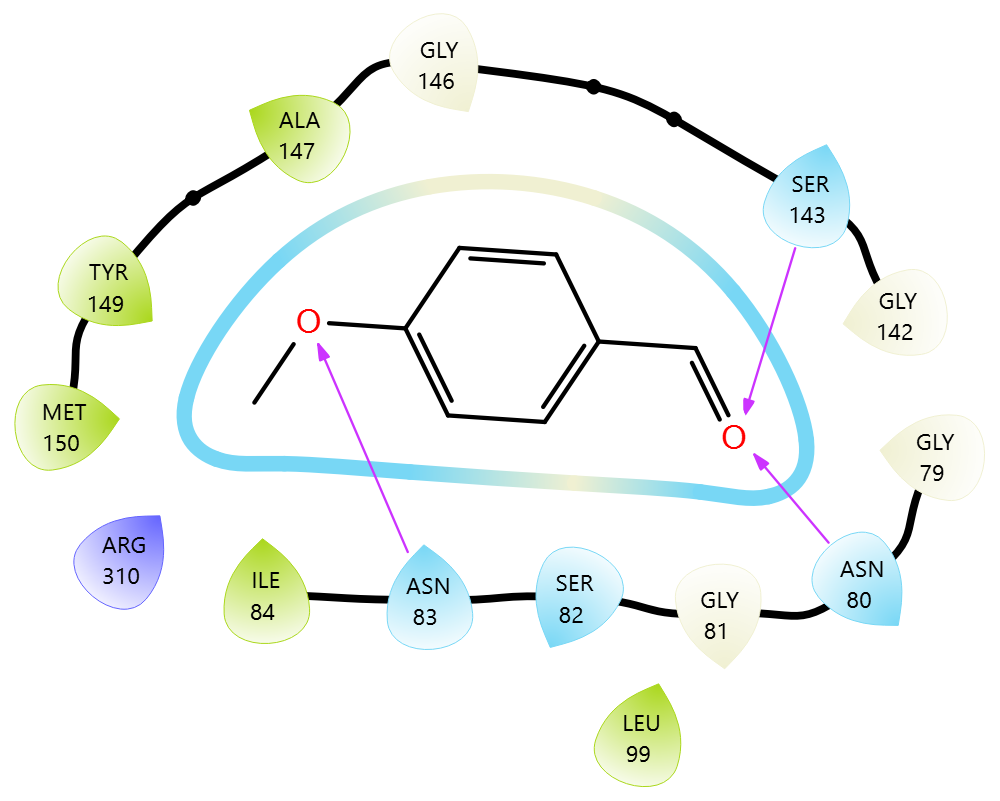
**

**Figure 25S.** 2D interaction diagram with 1HSK for **13**.


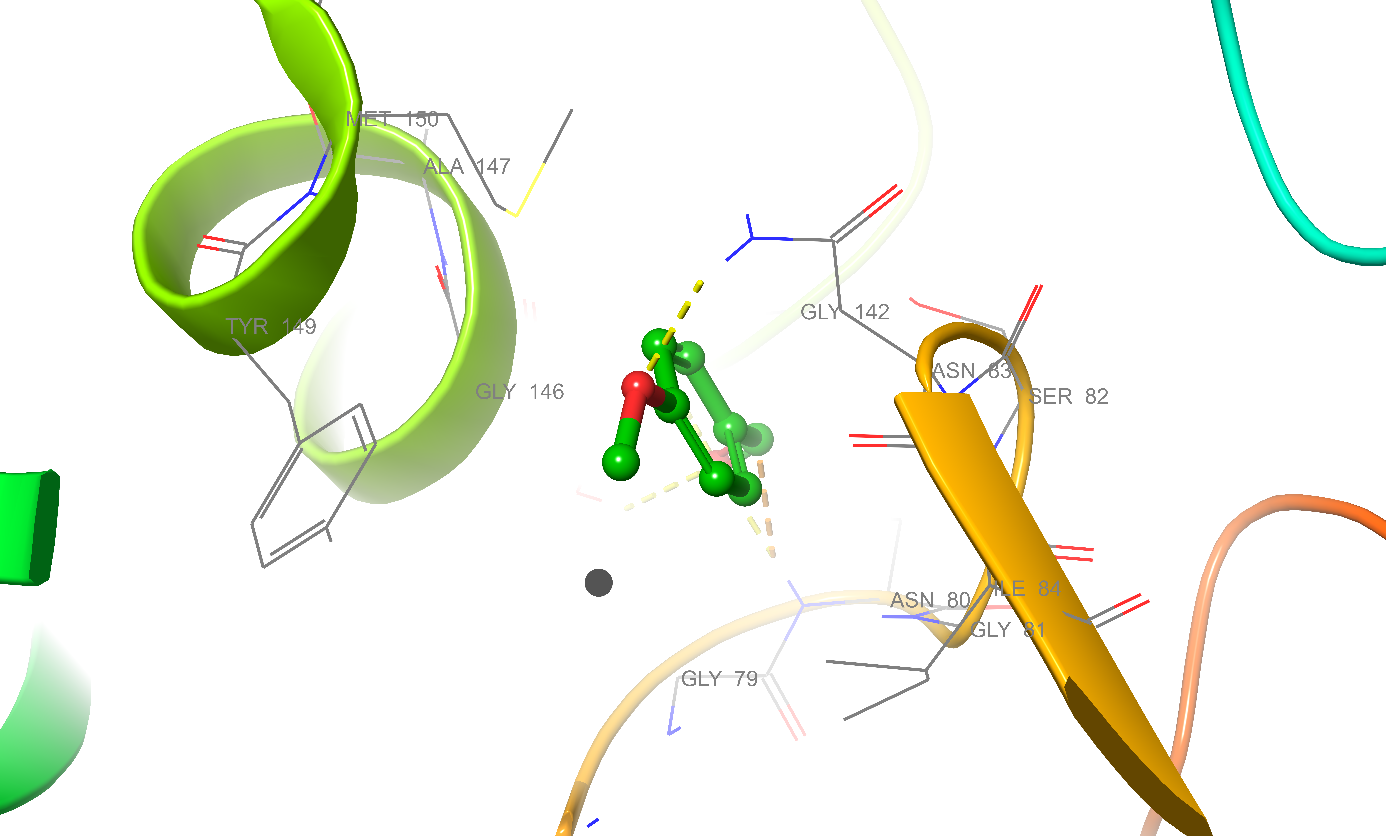


**Figure 26S.** 3D interaction diagram with 1HSK for **13**.

**
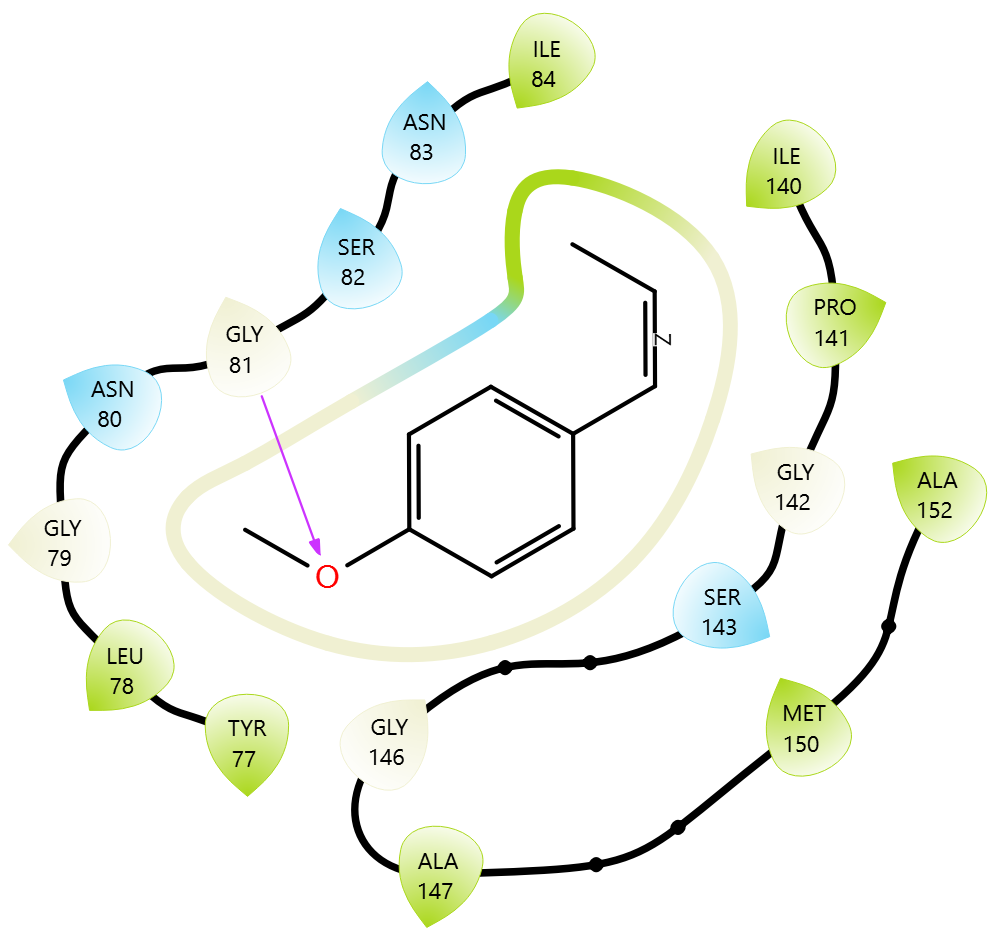
**

**Figure 27S.** 2D interaction diagram with 1HSK for **14**.


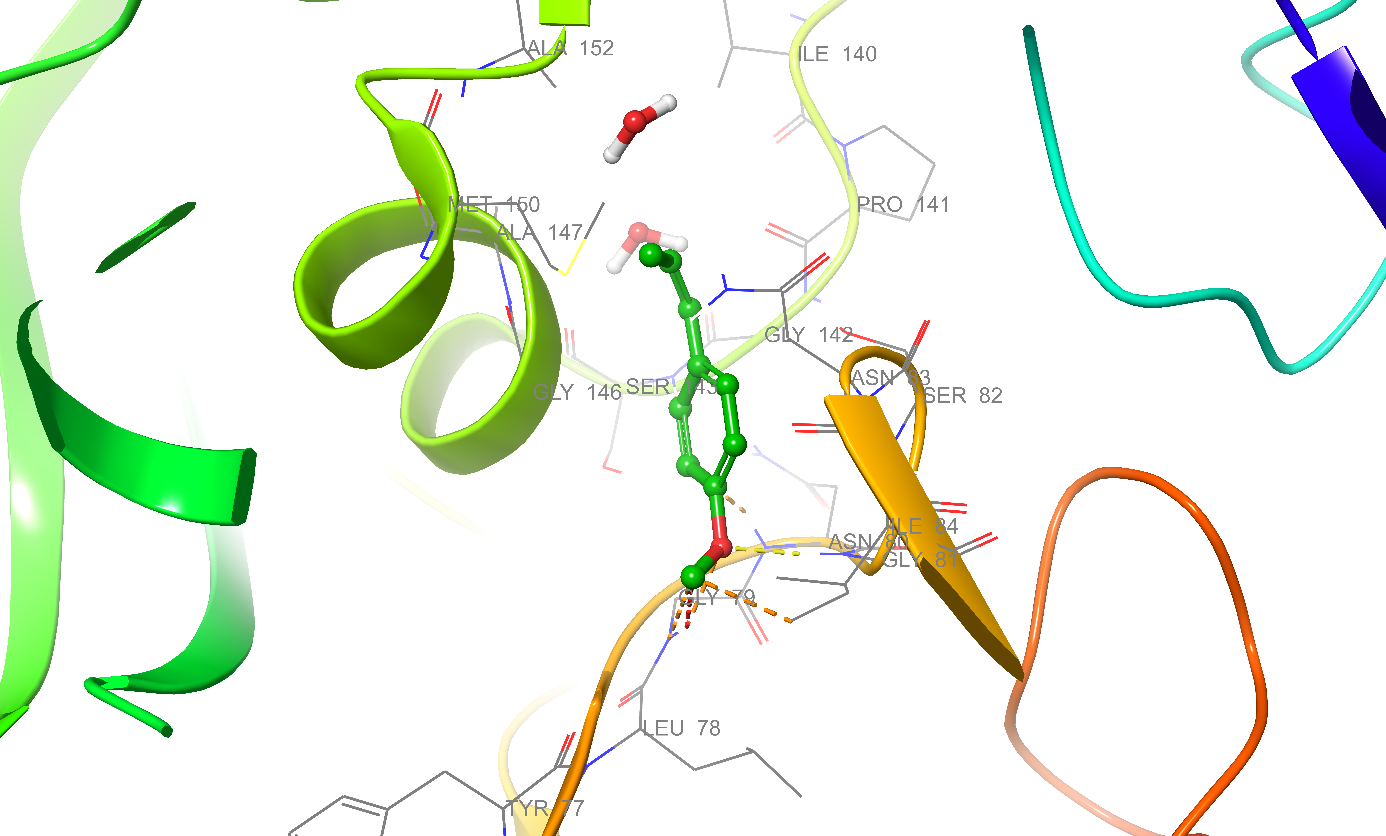


**Figure 28S.** 3D interaction diagram with 1HSK for **14**.

**
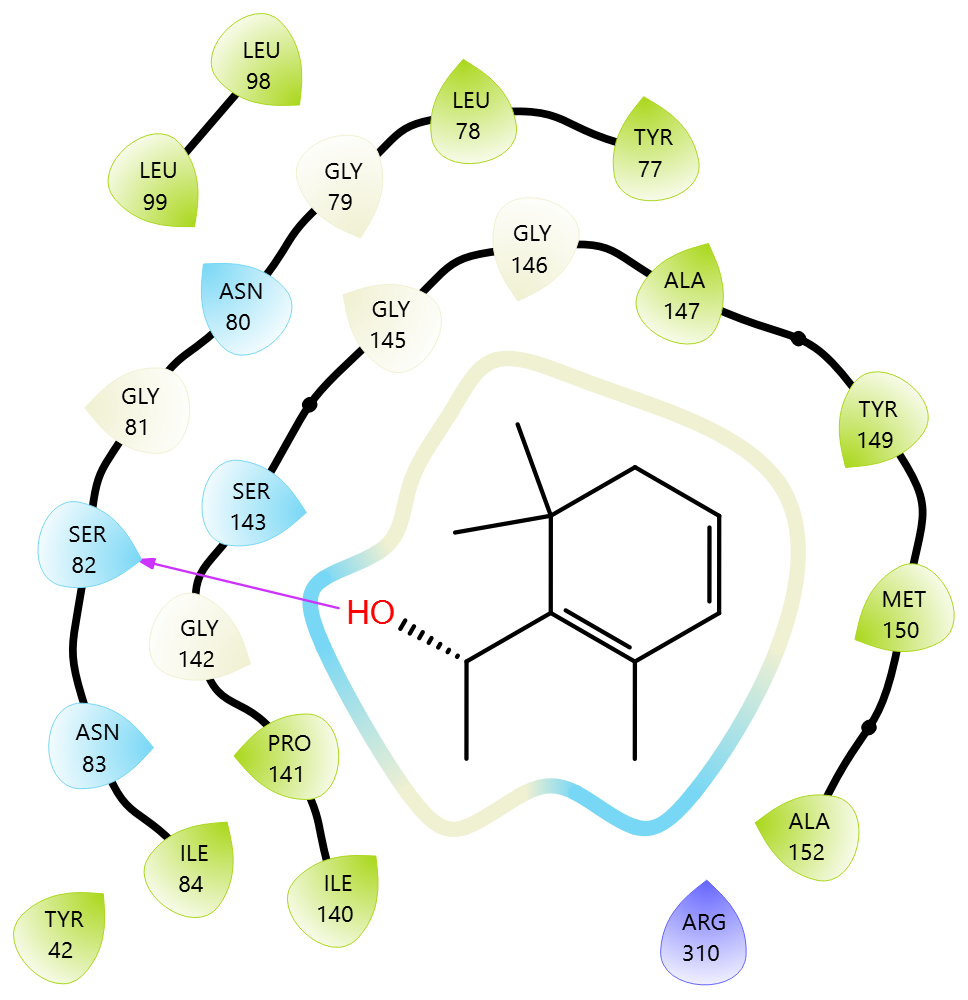
**

**Figure 29S.** 2D interaction diagram with 1HSK for **15**.


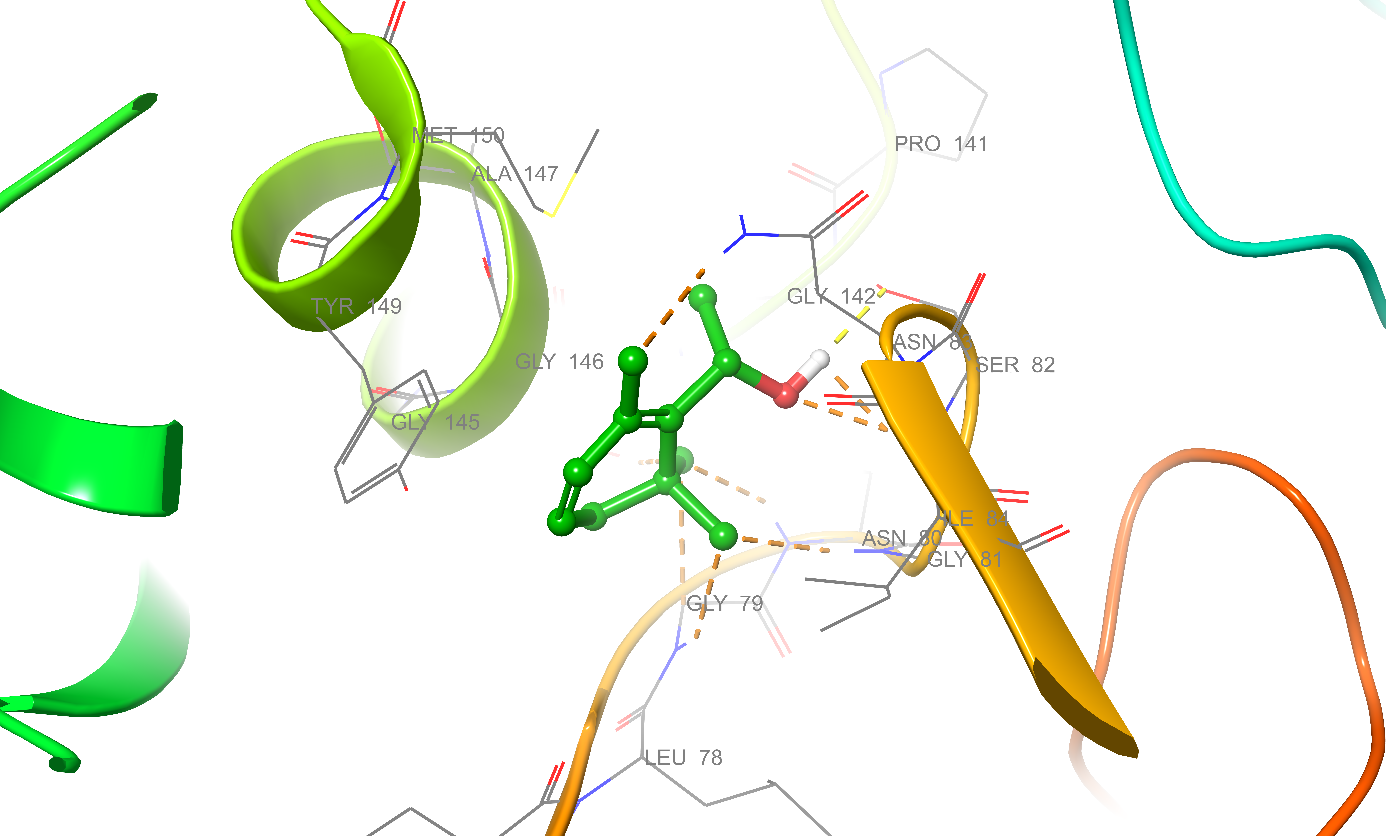


**Figure 30S.** 3D interaction diagram with 1HSK for **15**.

**
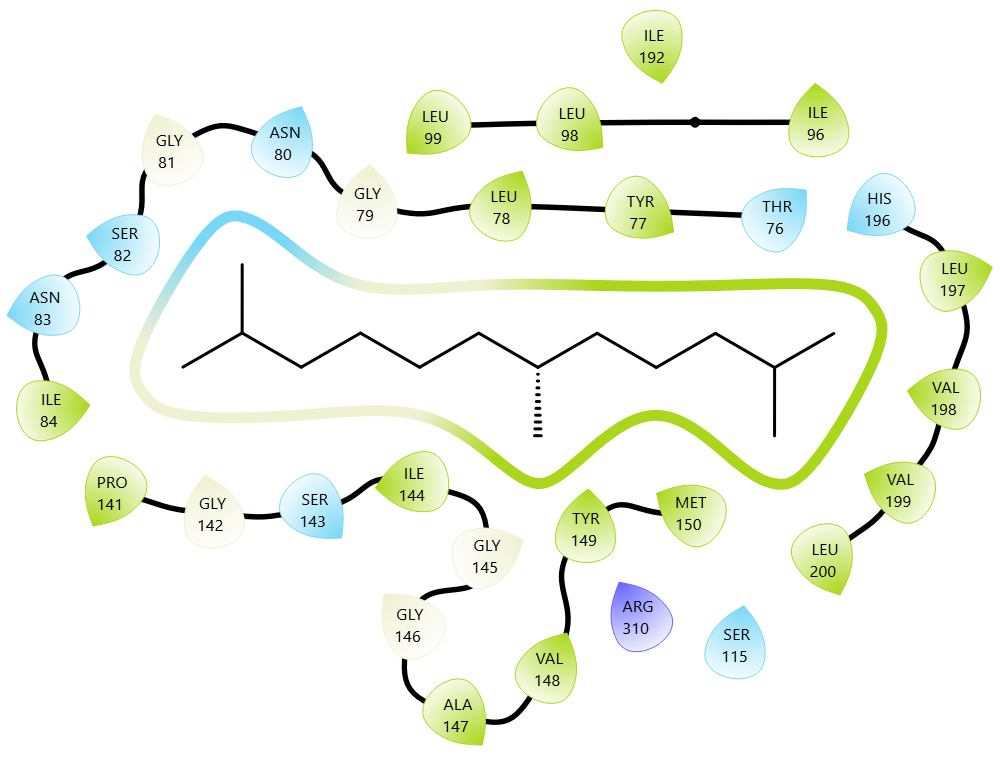
**

**Figure 31S.** 2D interaction diagram with 1HSK for **16**.


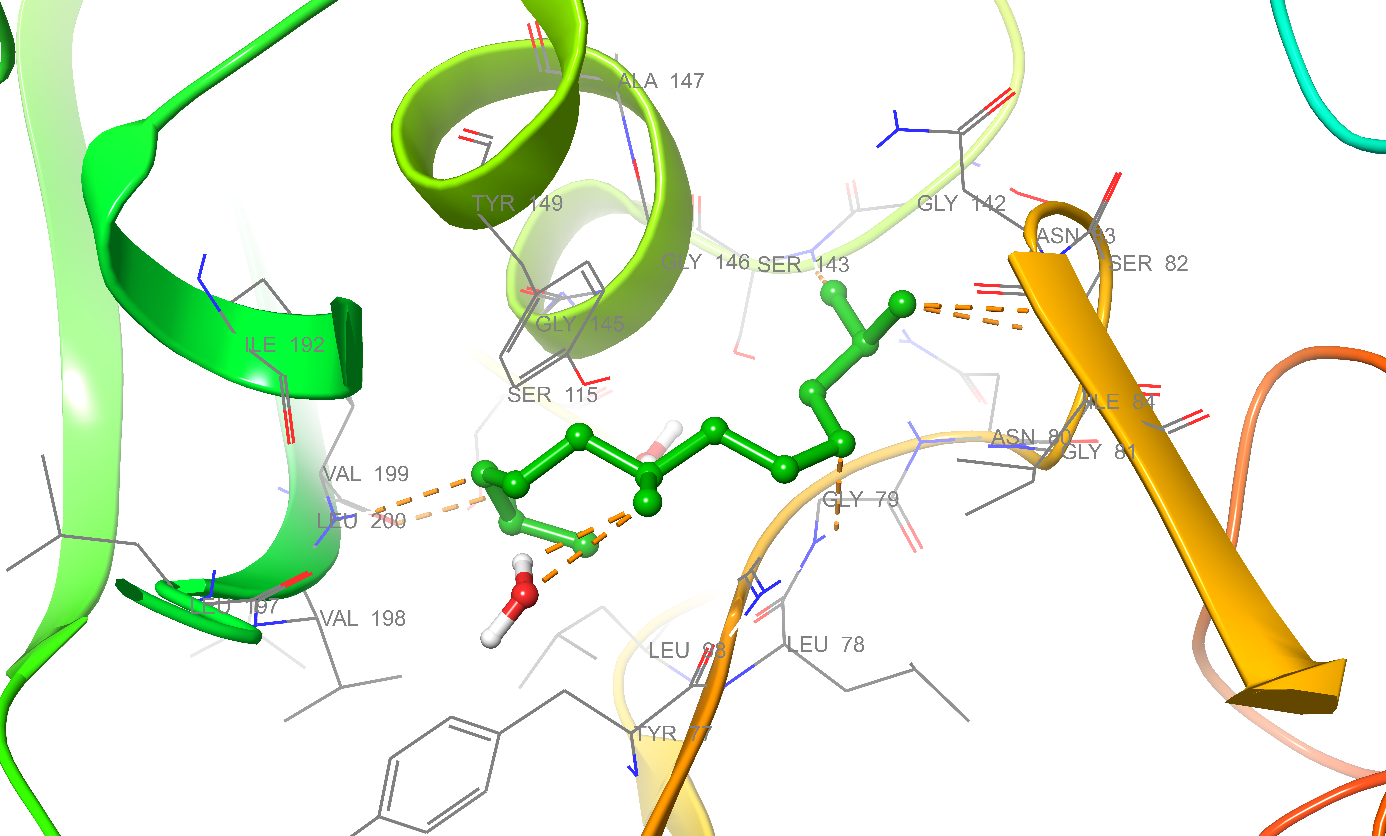


**Figure 32S.** 3D interaction diagram with 1HSK for **16**.

**
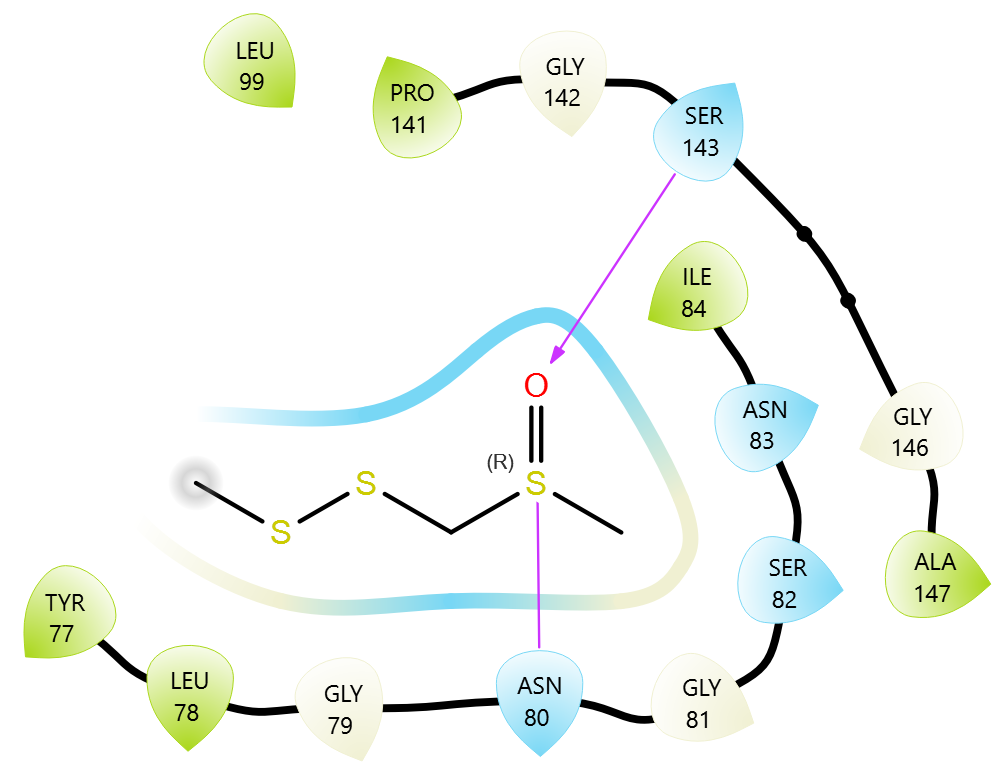
**

**Figure 33S.** 2D interaction diagram with 1HSK for **17**.


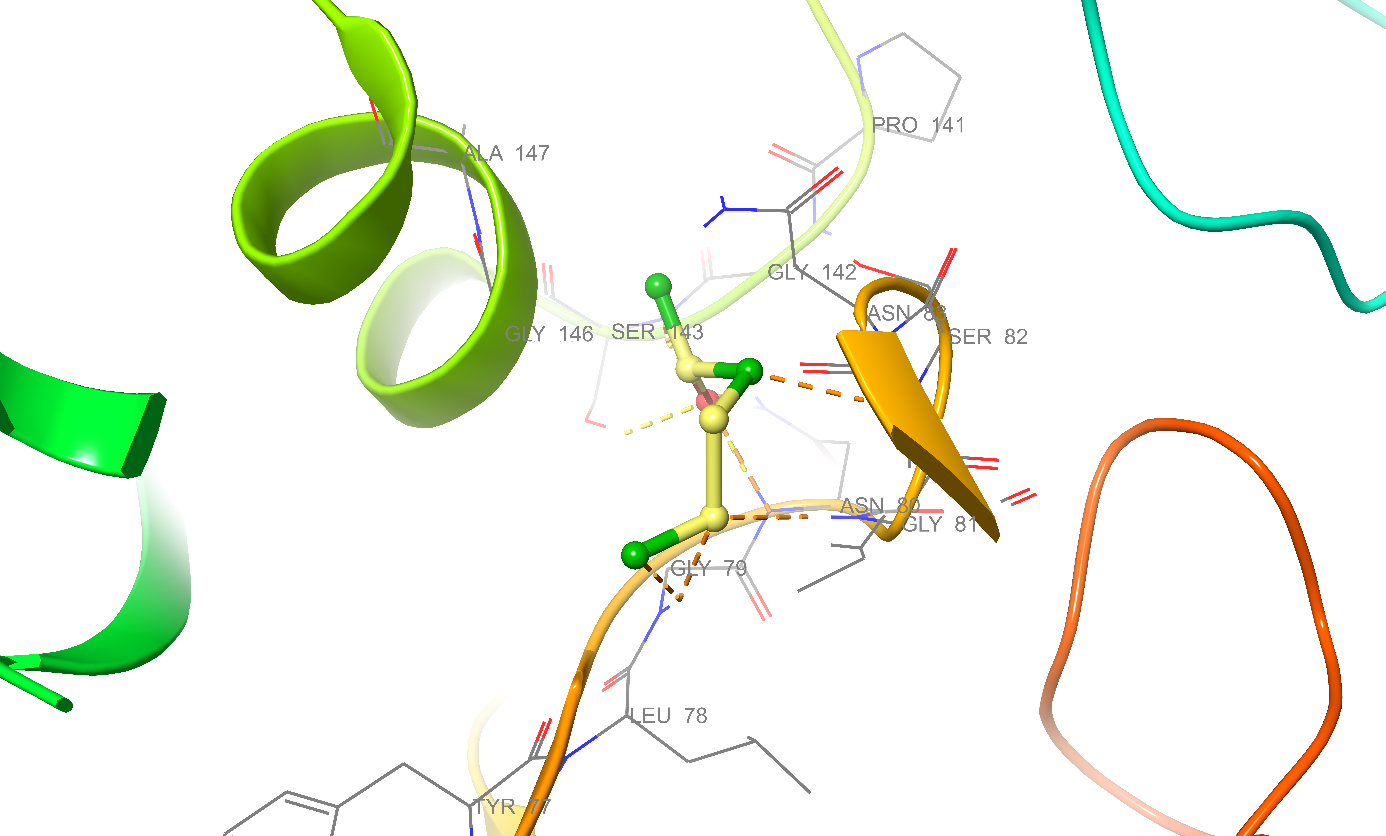


**Figure 34S.** 3D interaction diagram with 1HSK for **17**.

**
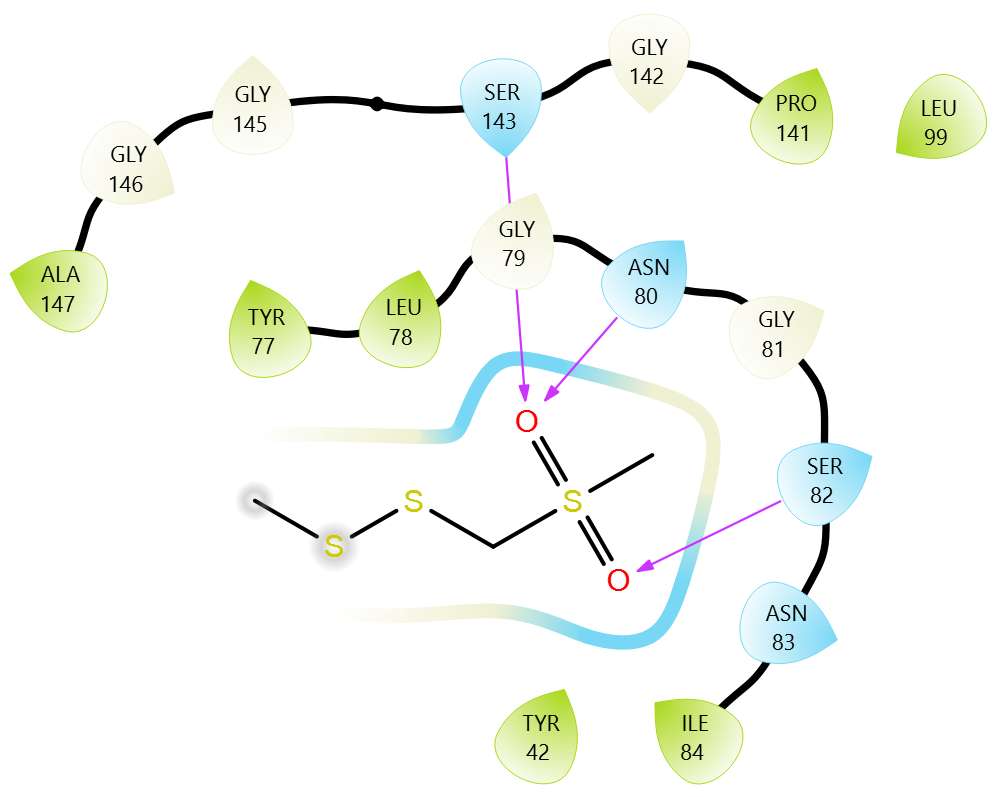
**

**Figure 35S.** 2D interaction diagram with 1HSK for **18**.


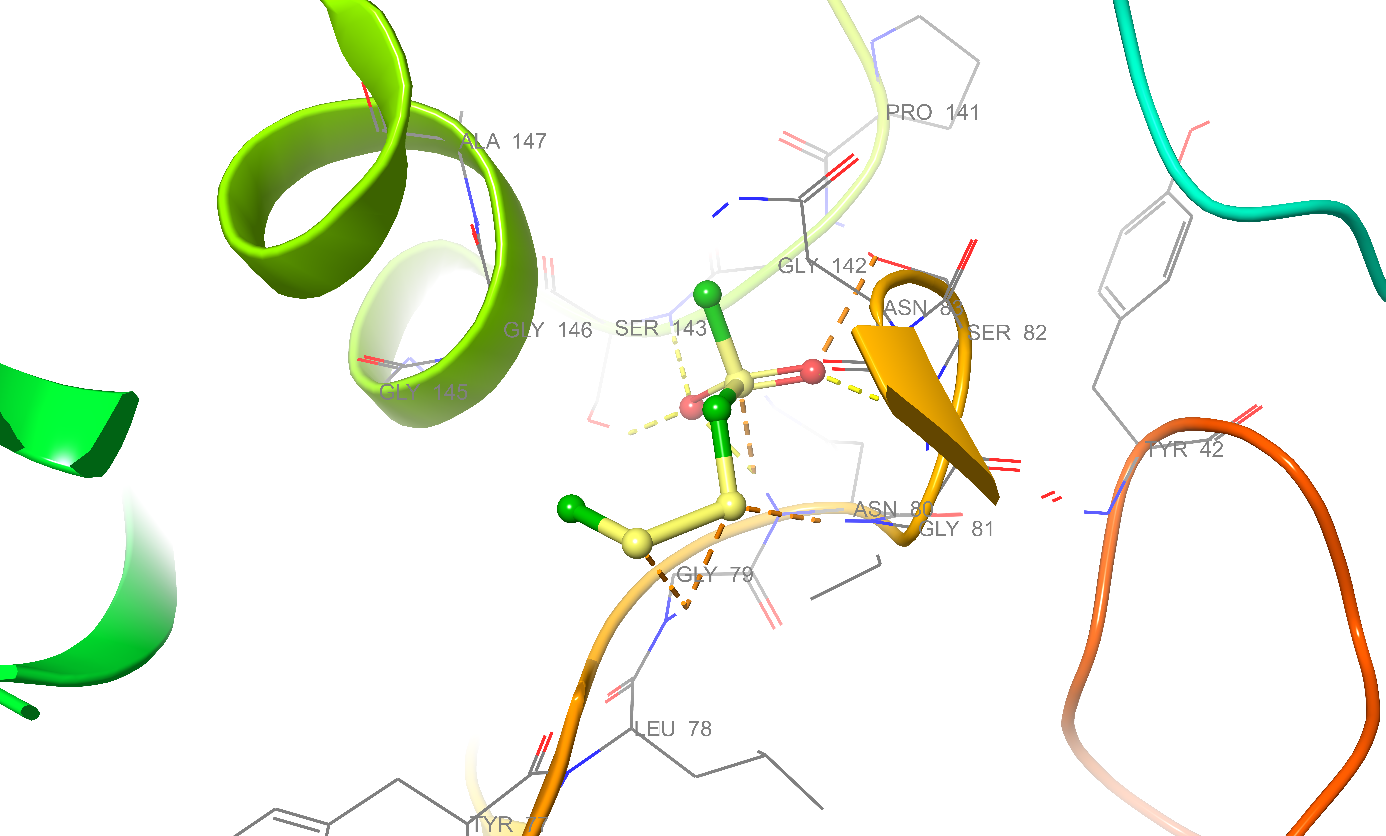


**Figure 36S.** 3D interaction diagram with 1HSK for **18**.

**
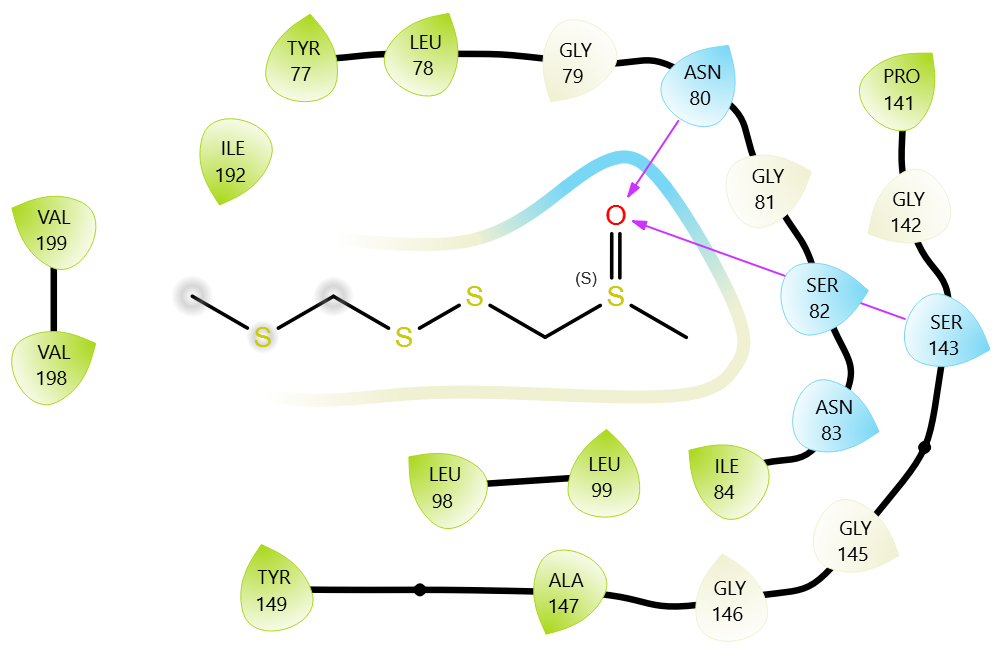
**

**Figure 37S.** 2D interaction diagram with 1HSK for **19**.


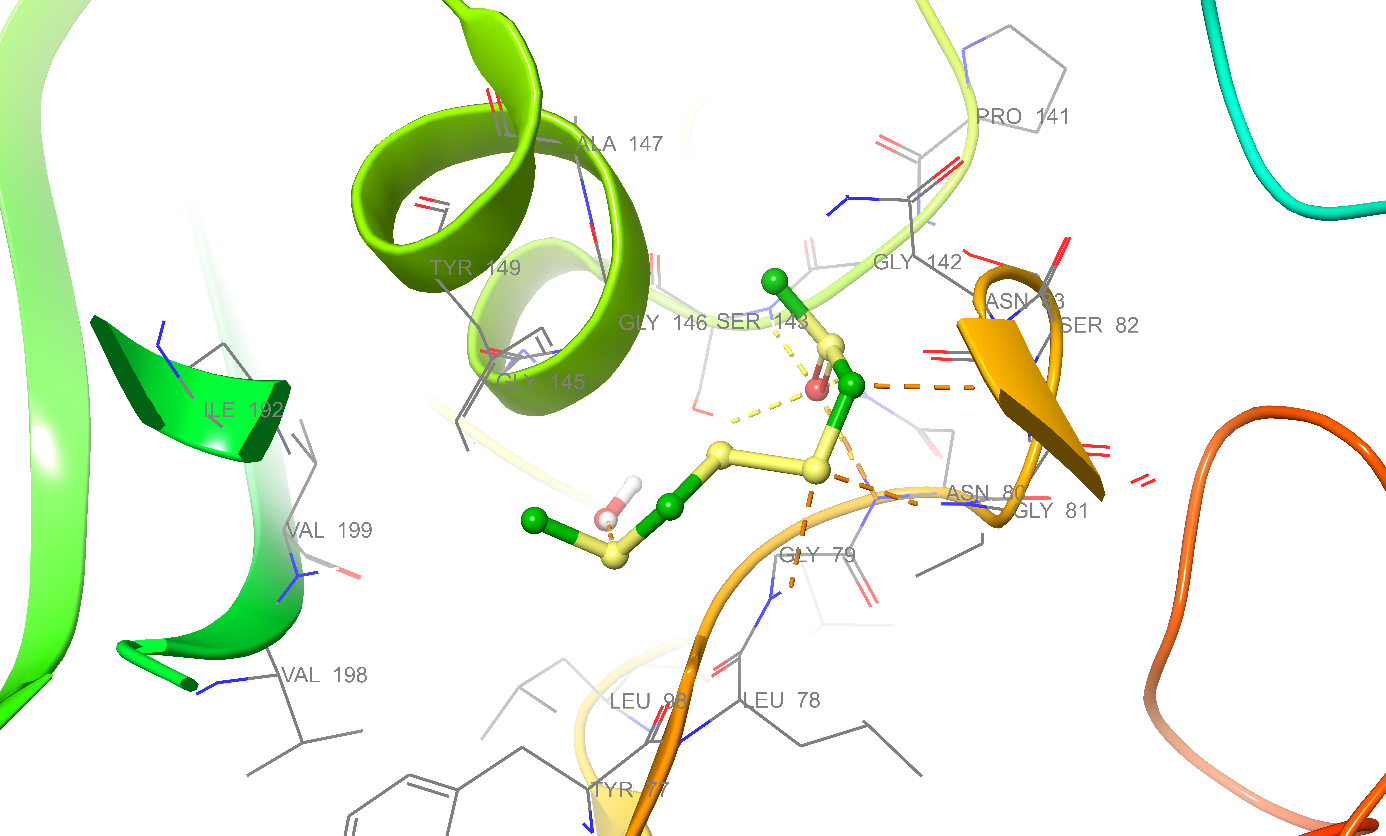


**Figure 38S.** 3D interaction diagram with 1HSK for **19**.

**
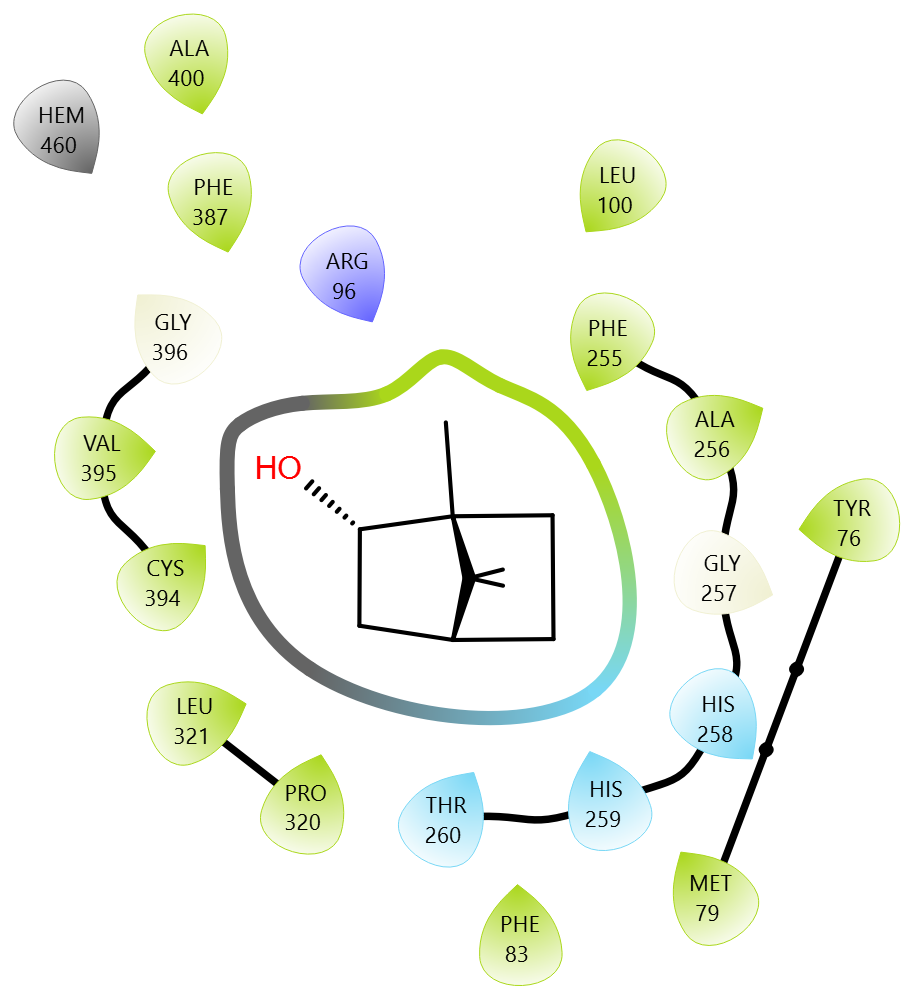
**

**Figure 39S.** 2D interaction diagram with 1EA1 for **8**.


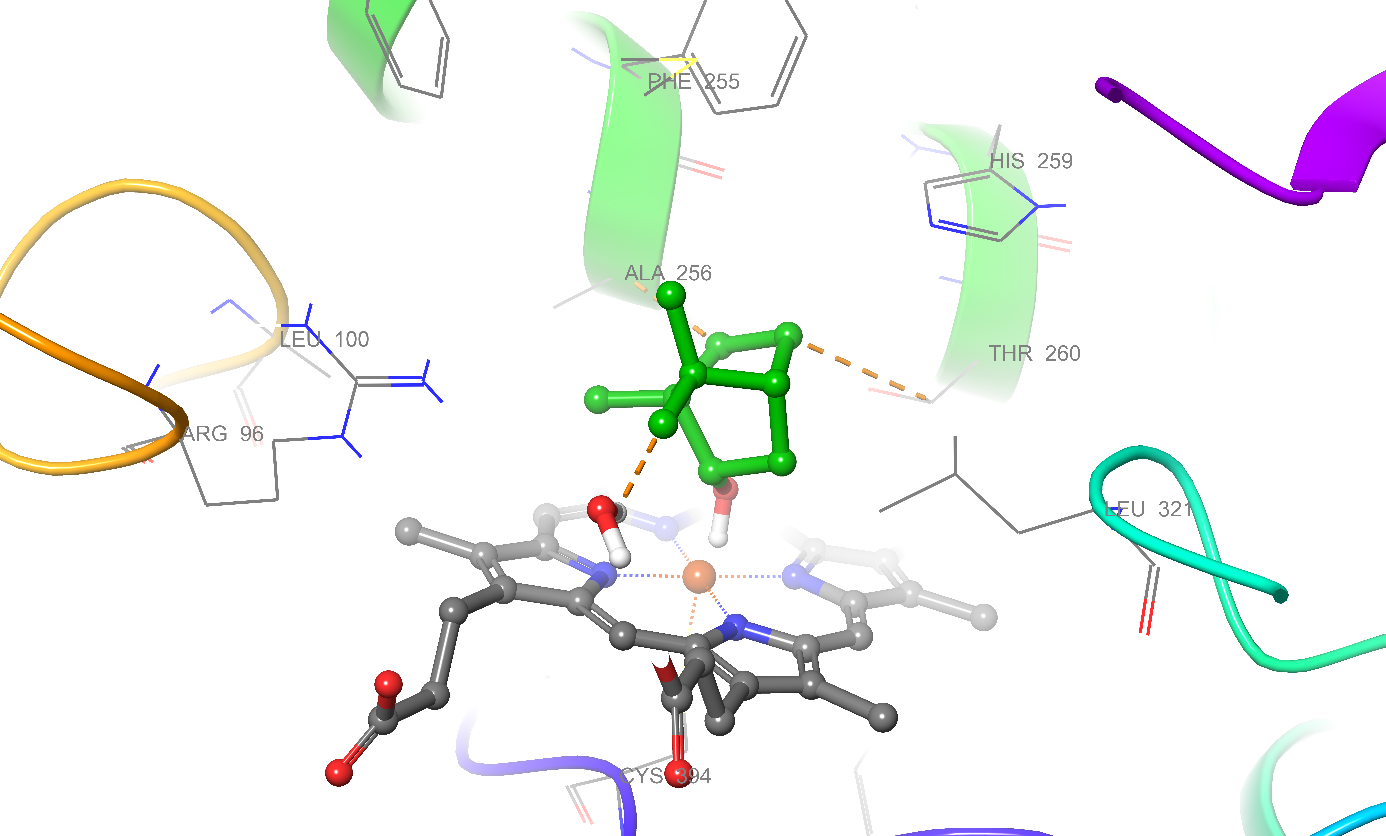


**Figure 40S.** 3D interaction diagram with 1EA1 for **8**.

**
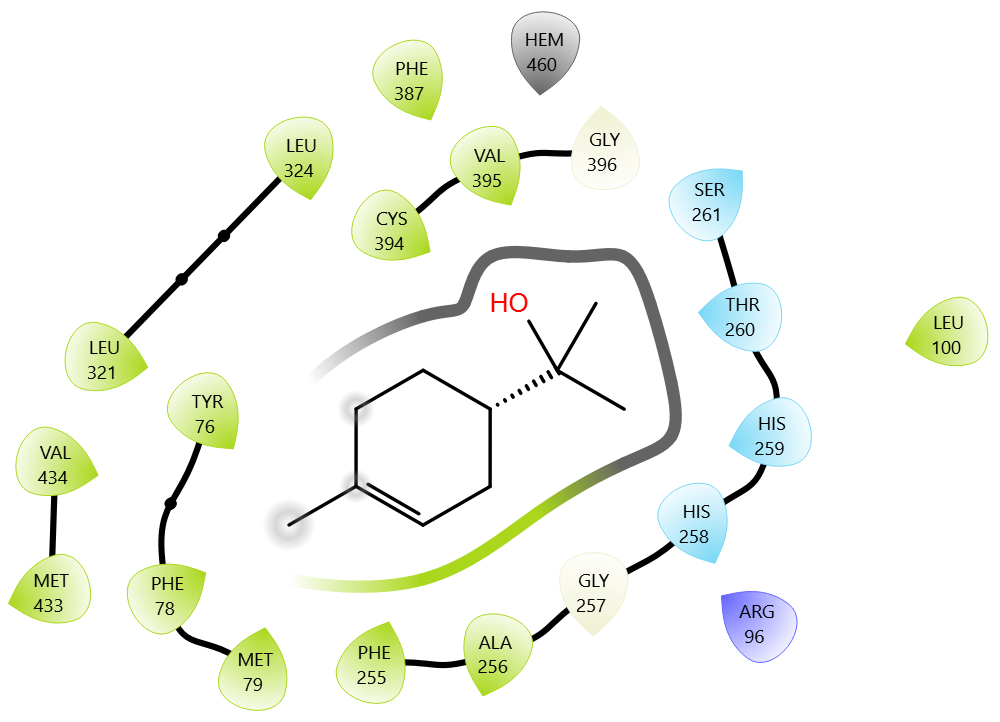
**

**Figure 41S.** 2D interaction diagram with 1EA1 for **9**.


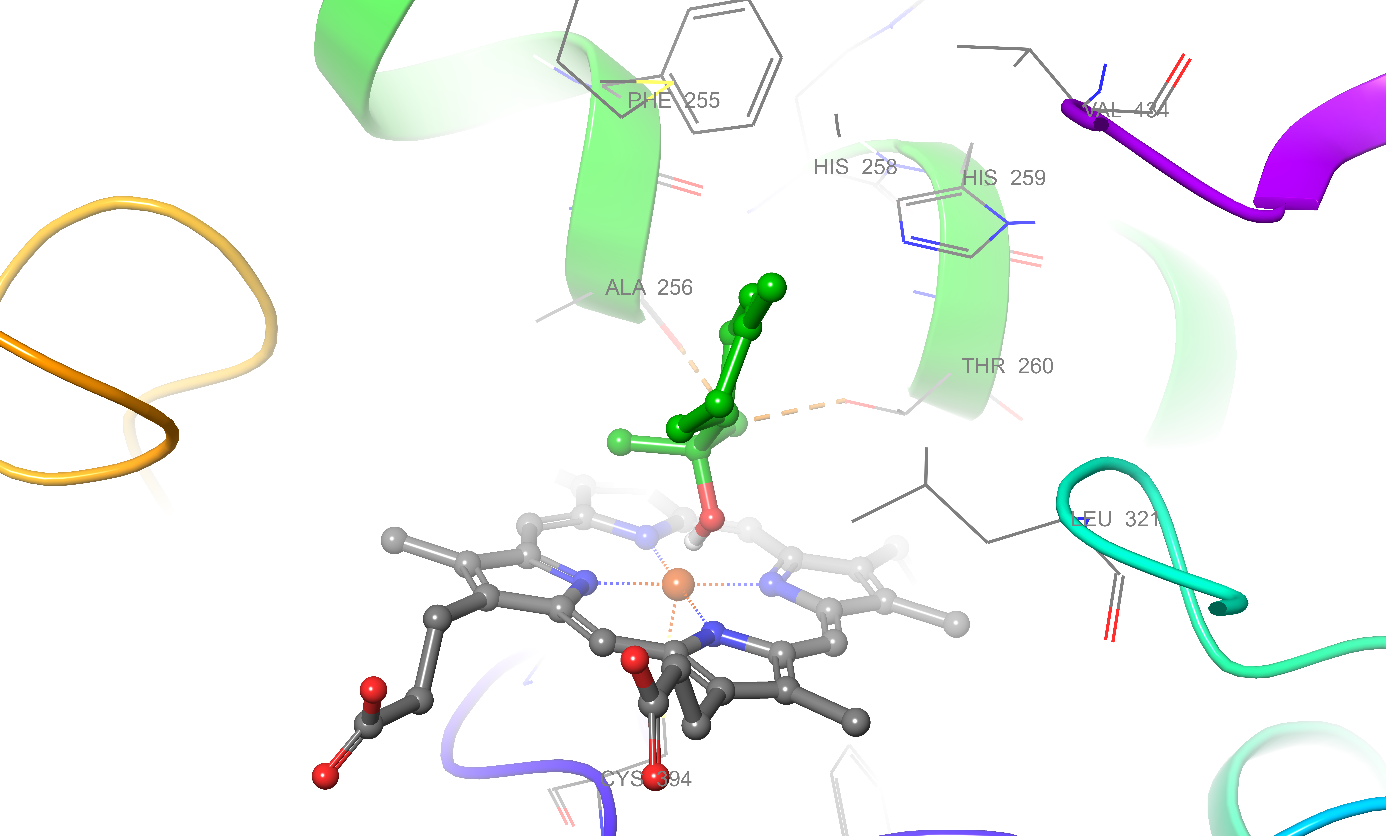


**Figure 42S.** 3D interaction diagram with 1EA1 for **9**.

**
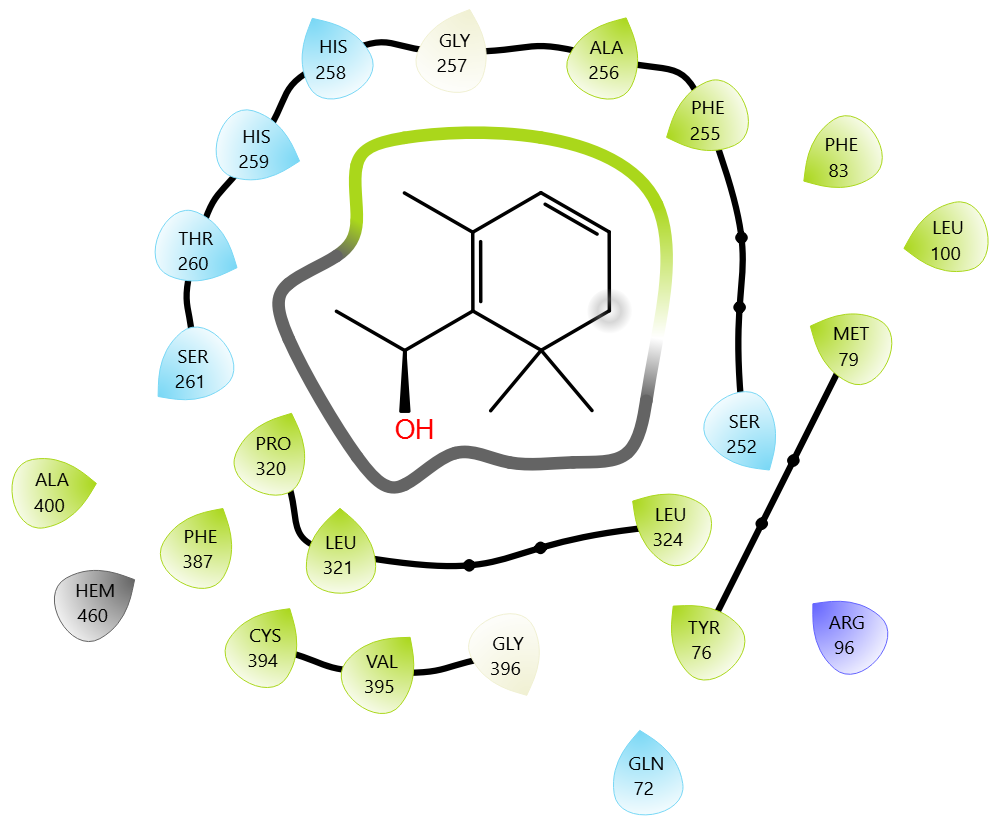
**

**Figure 43S.** 2D interaction diagram with 1EA1 for **15**.


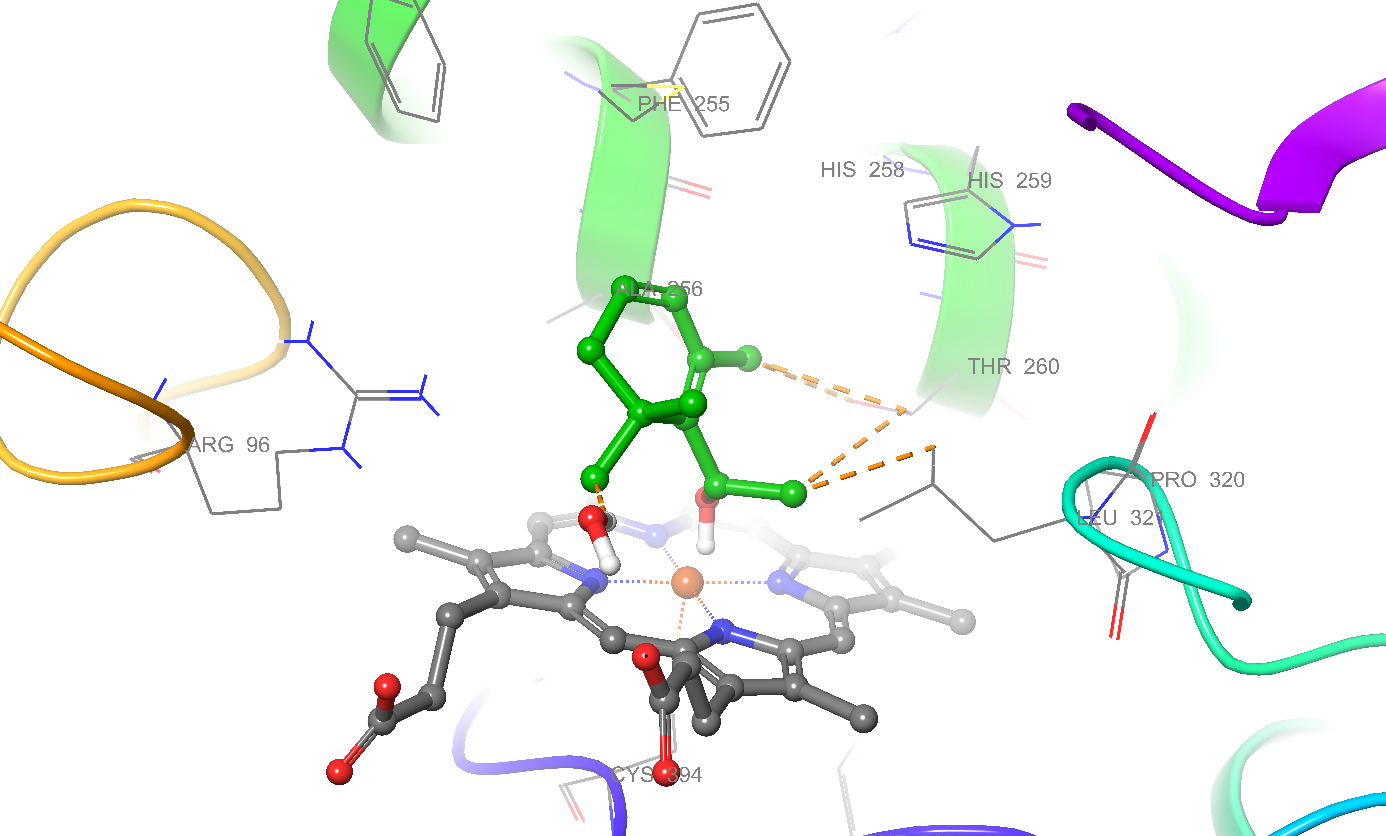


**Figure 44S.** 3D interaction diagram with 1EA1 for **15**.

**
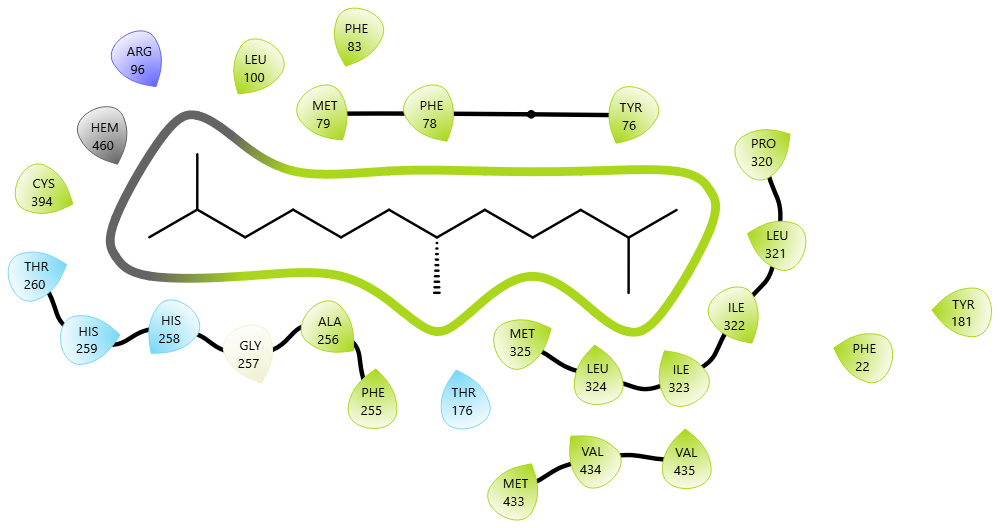
**

**Figure 45S.** 2D interaction diagram with 1EA1 for **16**.


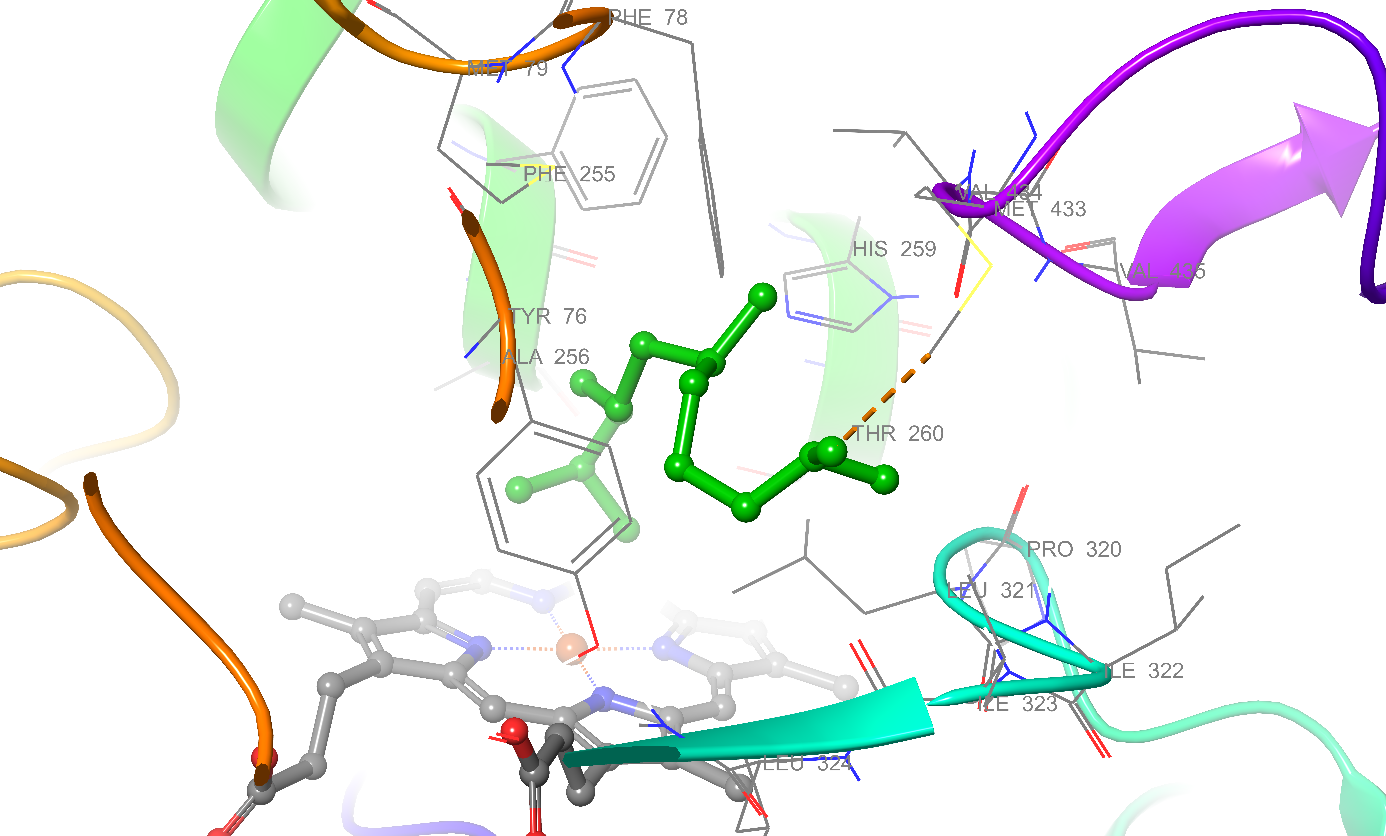


**Figure 46S.** 3D interaction diagram with 1EA1 for **16**.
